# Cyclic tri-adenylate controls a CARF-TM effector in type II Panoptes anti-phage systems

**DOI:** 10.1371/journal.pbio.3003934

**Published:** 2026-09-15

**Authors:** Sabine Grüschow, Peter Wotherspoon, Emma Hilton-Balfe, Shirley Graham, Malcolm F. White

**Affiliations:** School of Biology, University of St Andrews, St Andrews, United Kingdom; Monash University, AUSTRALIA

## Abstract

Cyclic nucleotide second messengers are used in all domains of life to amplify viral infection signals and activate cellular defences. In prokaryotes, CBASS (cyclic nucleotide**-**based antiphage signalling system) and type III CRISPR-Cas systems generate a range of cyclic nucleotides which bind and allosterically activate effector proteins to mount an anti-viral response. Viruses have evolved counter measures to antagonise these signalling pathways in the form of cyclic nucleotide sponges and phosphodiesterases that sequester or degrade these molecules to subvert immunity. Recently, the Panoptes system was shown to function as a guard against these viral tactics. The type I Panoptes polymerase, mCpol, generates cyclic dinucleotides as decoy molecules that, when sequestered by phage proteins, results in the activation of the membrane-permeabilising effector 2TMβ to halt the phage infection cycle. Here, we investigate the type II Panoptes system, demonstrating that it generates cyclic tri-adenylate (cA_3_) to maintain a CRISPR-associated Rossmann fold-transmembrane (CARF-TM) effector in an inactive, dimeric state. When cA_3_ is sequestered or degraded, the CARF protein undergoes conformational changes. In vivo, the absence of cA_3_ results in membrane disruption and growth arrest. Type II Panoptes provides defence against phages that express the cA_3_-degrading enzyme Acb1; phage escapers introduce mutations into the *acb1* gene to avoid triggering the Panoptes system. These findings expand our understanding of the guard systems that constitute a fascinating component of the bacterial immune system.

## Abstract 2

Phages can evade bacterial immunity by disrupting antiviral signals such as cyclic tri-adenylate (cA3), triggering the Panoptes defense systems. This study shows that Type II Panoptes detects cA3 loss and activates a CARF-TM effector to disrupt membranes and stop infection.

## Introduction

In recent years, a large number of antiviral defence systems have been discovered in prokaryotes, leading to the concept of the bacterial immune system [1,2]. One major approach, used in both prokaryotes and eukaryotes, is the generation of nucleotide second messengers by specialised nucleotide cyclase enzymes activated by viral infection [3,4]. Notable examples include the cGAS/STING pathway in metazoa which signals via cGAMP [5] and its bacterial equivalent CBASS (cyclic nucleotide-based antiphage signalling system) [6], Pycsar (Pyrimidine cyclase system for antiphage resistance) which generates cyclic pyrimidines [7] and the type III CRISPR-Cas system signalling via production of cyclic oligoadenylate (cOA) species [8,9]. This approach has the advantage of achieving amplification of the primary signal of infection—for example, the type III CRISPR system detects 1 viral mRNA molecule and generates over 1,000 cyclic nucleotides in response [10]. Viruses, in turn, target the cyclic nucleotides to neutralise cellular defences using specialised phosphodiesterases that degrade cGAMP in eukaryotes [11] and bacteria [12], ring nucleases that target cOA species to nullify CRISPR-based immunity [13] and sponge proteins that bind cyclic nucleotide second messengers [14,15]. Sponge proteins are particularly adept at sequestering a range of cyclic nucleotides used by immune defence systems and are widely encoded by phage [16,17].

Type III CRISPR systems use their catalytic Cas10 subunit, which has two Palm polymerase domains, to generate cOA species ranging from 3 to 6 AMP subunits [8,9]. These activate a diverse range of effector proteins, many of which include a CRISPR-associated Rossmann Fold (CARF) sensor domain, to provide an immune response [18,19]. The recent discovery of variants that make the signalling molecule SAM-AMP by conjugating S-adenosyl methionine and ATP [20] underscores the flexibility of the polymerase to accept a range of building blocks for signal generation. In 2015, Burroughs and colleagues described a minimal CRISPR-related polymerase (mCpol), distantly related to Cas10, which was sometimes associated with CARF domain proteins characteristic of type III CRISPR effectors [21]. In some cases, the mCpol cyclase is fused to a Csx1-family ribonuclease effector protein, potentially representing a single-protein antiviral defence system that functions via cyclic nucleotide signalling [22]. In other cases, 2 gene operons include mCpol next to a gene encoding a predicted membrane-bound effector [22]. mCpol has been suggested as the ancestral protein that gave rise to Cas10 and the evolution of the class 1 CRISPR systems [22].

**Fig 1:**
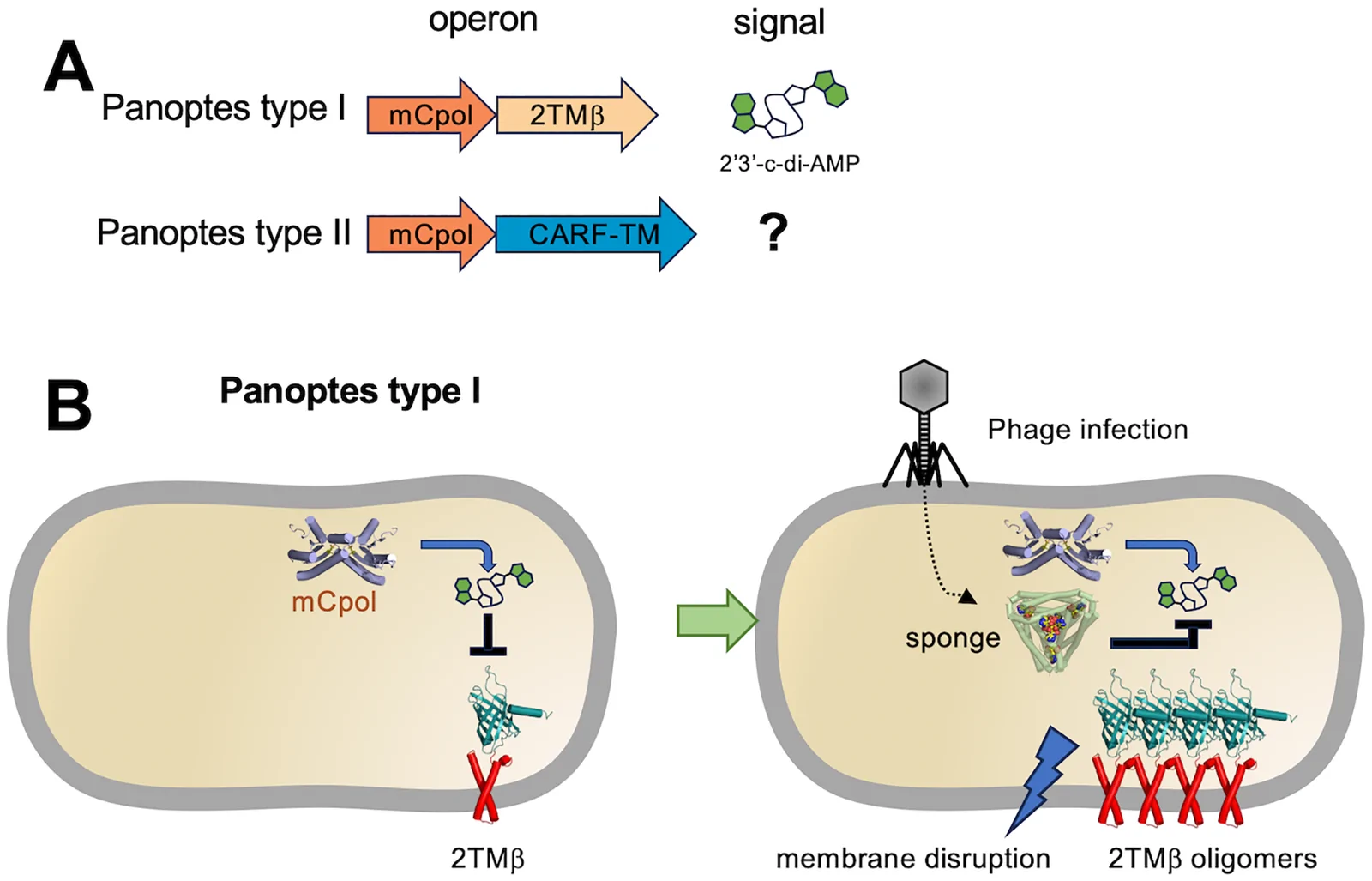
Organisation and mechanism of the Panoptes System. **A.** Operon organisation and major cyclic nucleotide signal for representative type I and II Panoptes systems. **B.** In type I Panoptes systems, mCpol is constitutively active, generating signalling molecules that inactivate the cognate effector, 2TMβ. When an infecting phage expresses a sponge protein, the cyclic oligonucleotide pool is depleted. The Panoptes effector oligomerises, leading to membrane disruption and growth arrest or cell death.

Recently, two research groups independently demonstrated that one type of mCpol-containing system, which they named “Panoptes” (henceforth Panoptes type I, Fig 1A), generates cyclic dinucleotides constitutively, maintaining an associated 2TMβ (2 *trans-*membrane helix, β-strand rich) effector in an inactive state [23,24]. This contrasts with CBASS, and other defence systems relying on cyclic oligonucleotide signalling, which require an active phage infection to trigger cyclic nucleotide synthesis. When a phage expressing an anti-defence sponge protein such as Acb2 infects cells encoding Panoptes type I, the cyclic dinucleotide is depleted, resulting in activation of the 2TMβ-effector, leading to growth arrest and thwarting the phage infection (Fig 1B). Panoptes is thus an example of a guard system that may often work in conjunction with CBASS defence to prevent phage from utilising a cyclic nucleotide depletion strategy.

Although biochemical studies have focussed on Panoptes with a 2TMβ effector, about 40% of systems (hereafter Panoptes type II) are associated with an alternative CARF-family effector with a transmembrane domain (CARF-TM) [24]. Here, we demonstrate that the type II Panoptes mCpol generates a cyclic trinucleotide (cA_3_) product which is bound by the associated CARF-TM effector. The system provides immunity against plasmids and phage expressing antidefence proteins that bind or degrade cA_3_. The absence or removal of cA_3_ from CARF-TM results in structural changes that ultimately lead to outer membrane (OM) permeabilization and render cells incapable of forming colonies. These data demonstrate another facet of Panoptes-mediated immunity and the potential to guard type III CRISPR-Cas as well as CBASS defence systems.

## Results

### Type II Panoptes signals via cyclic tri-adenylate (cA_3_)

**Fig 2:**
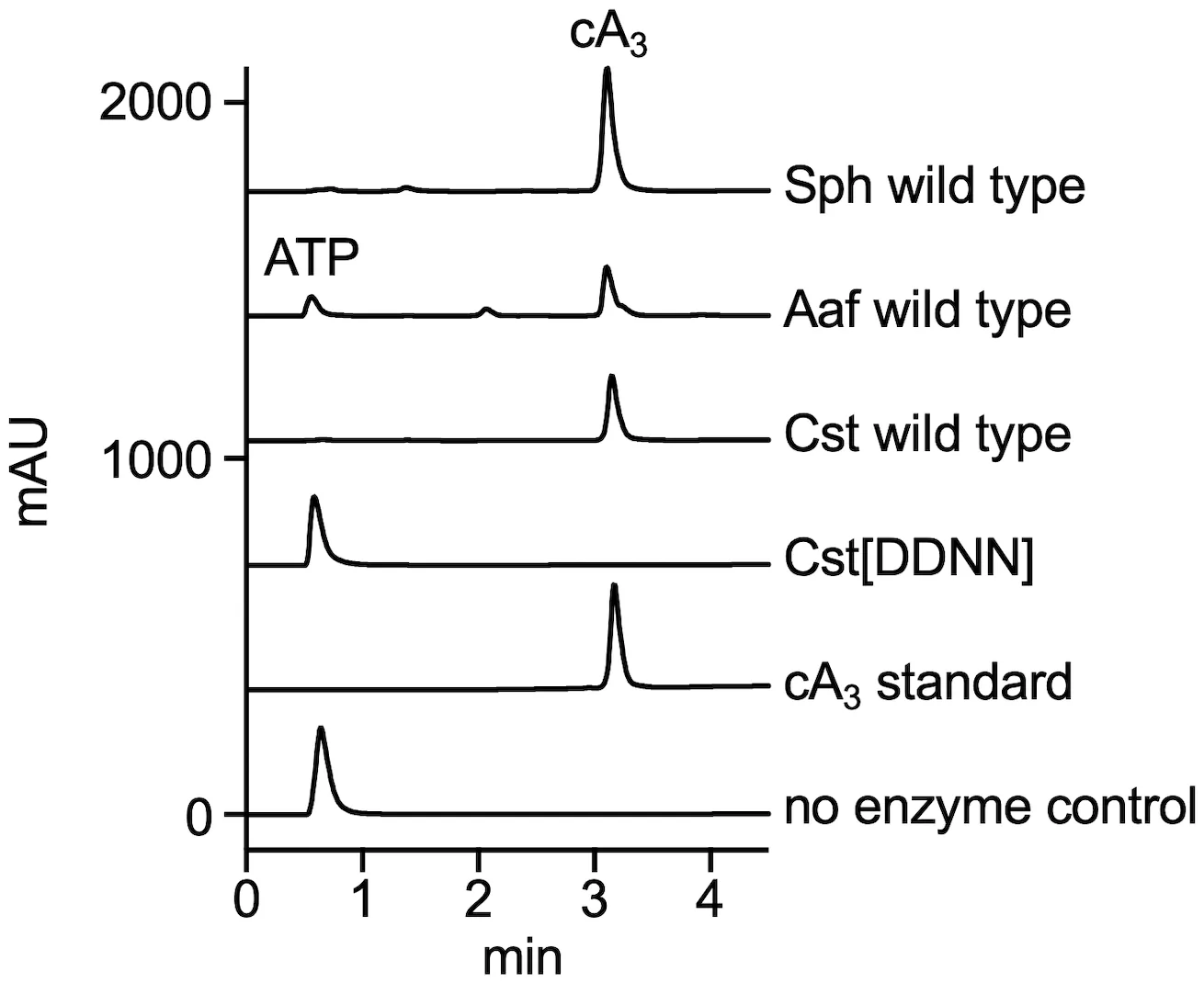
Type II mCpols synthesise 3′3′3′-cA_3_. HPLC analysis of Cst, Sph and Aaf mCpol reaction products, showing production of cA_3_ in the presence of ATP and Mg^2+^. The Cst mCpol[D63N, D64N] variant (Cst[DDNN]) was catalytically inactive. The data underlying this Figure can be found in S1 Data.

While the characterised type I Panoptes systems synthesise cyclic dinucleotides and utilise a 2TMβ-family effector, type II systems with a CARF-TM effector remain unstudied. We designed synthetic genes encoding mCpol and CARF-TM from the cyanobacterium *Cylindrospermum stagnale* PCC7417 (Cst) for expression in *E. coli* and purified the recombinant proteins. The Cst mCpol protein was tested for nucleotide cyclase activity and shown to synthesise 3′3′3′-cA_3_ in the presence of ATP and Mg^2+^ (Fig 2). The presence of other NTPs did not alter the reaction product. Two further mCpol enzymes associated with a CARF-TM effector, from *Sphingomonas* (Sph) and *Ahniella affigens* (Aaf), also synthesised 3′3′3′-cA_3_ as shown by HPLC analysis and co-injection with synthetic 3′3′3′-cA_3_ (S1 Fig). The turnover number for all three mCpol enzymes was approximately 1 h^−1^ in vitro (S2 Fig), making mCpol significantly slower than Cas10 from Type III CRISPR systems [25]. Although in vitro reaction rates should be treated with caution, these data suggest mCpol may generate relatively low concentrations of cA_3_ in cells. This could be consistent with a function as a decoy molecule for phage sponges and phosphodiesterases, providing sensitivity to changes in cA_3_ concentration in vivo. Residues D11 and D63 of Cst mCpol correspond to the canonical metal-binding residues of Cas10 and Palm family polymerases more generally. We mutated D63 and the neighbouring D64 to generate Cst mCpol[D63N/D64N]. This variant was completely inactive, as expected (Fig 2).

### Type II Panoptes is active in vivo and responds to cA_3_ levels in cells

**Fig 3:**
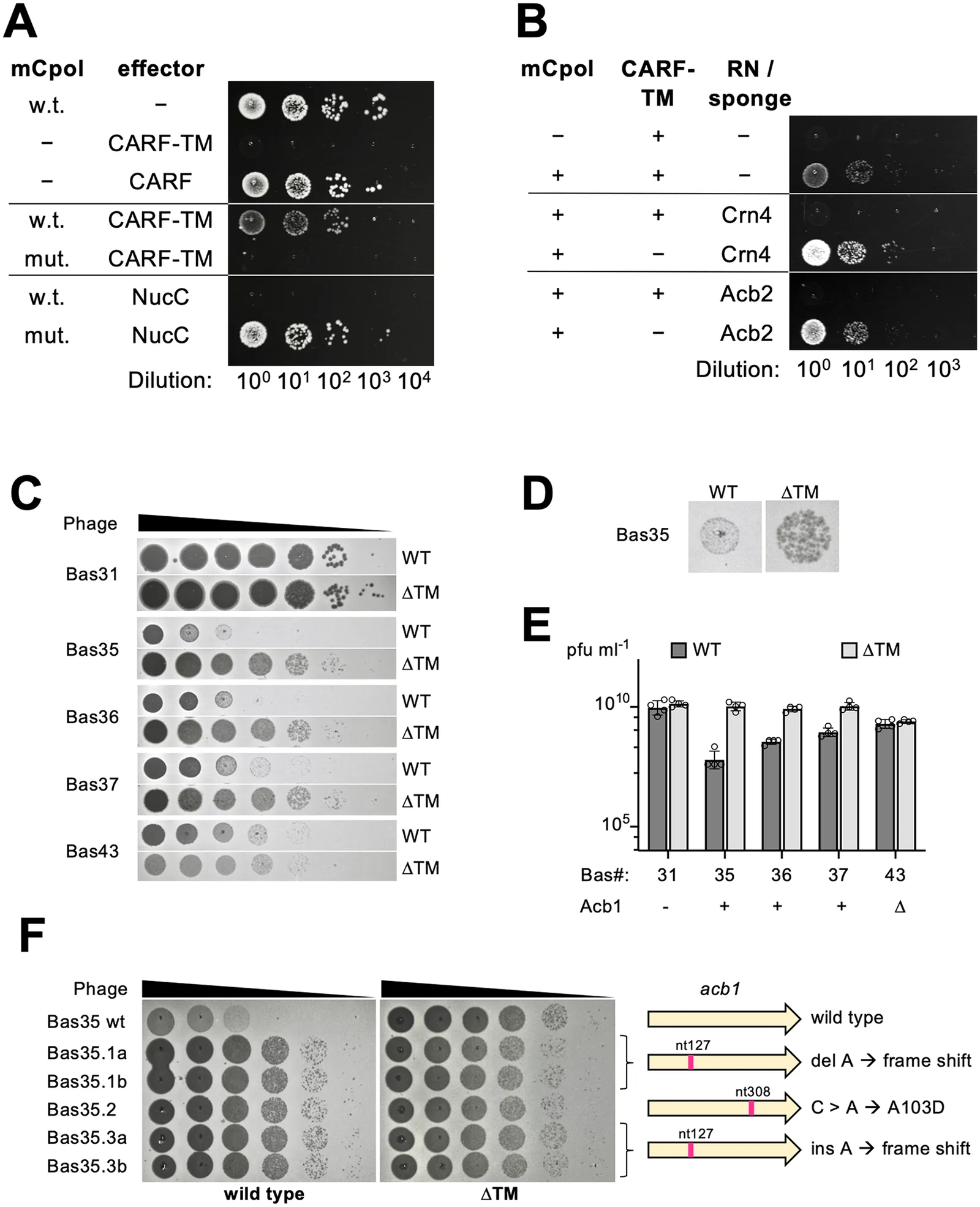
Panoptes type II is functional in vivo. **A.** Plasmid challenge assay using the Cst Panoptes system in *E. coli*. Cells expressing the wild-type or inactive variant mCpol were transformed with a plasmid that expresses the CARF-TM, CARF or NucC effectors. The cA_3_-activated nuclease NucC prevented cell growth only in the presence of mCpol, whereas CARF-TM did so in its absence. The transmembrane domain of CARF-TM was required for activity. (w.t.: wild type; mut.: catalytically inactive mCpol[D63N, D64N]). **B.** Plasmid challenge assay as in **(A)**, where a second gene is introduced on the plasmid encoding the effector. The presence of a cA_3_-degrading ring nuclease (Crn4) or a phage sponge protein (Acb2) activated CARF-TM activity in the presence of mCpol, leading to toxicity. **C.** Efficiency of plaquing (EOP) assay using the Aaf Panoptes system (mCpol and either CARF-TM (WT) or CARF (ΔTM) in *E. coli*. Bas31 (T5 family) was insensitive to mCpol. Phages Bas35-37 were sensitive while closely related Bas43, which carries a nonsense mutation in the *acb1* gene, escapes defence. **D.** Close-up of plaques for Bas35, showing reduction in plaque size when Panoptes is active. **E.** Quantification of EOP for replicates of the data shown in (C). Four biological replicates were analysed and plaques counted. Mean plaque numbers and standard deviations are shown along with individual data points. The data underlying this panel can be found in S1 Data. **F.** EOP assay of Bas35 and escaper variants (1a, 1b, 2, 3a, 3b) against wild type and inactive (ΔTM) Aaf type II Panoptes. The observed mutations are mapped onto their position in the *acb1* gene. 1a,b and 3a,b represent biological replicates purified from a single plaque and sequenced independently.

To test the activity of Panoptes type II in vivo, we performed transformation assays, where the effector was introduced to a strain containing the mCpol cyclase. A serial dilution of the transformation mixture was then plated onto selective medium and both cyclase and effector expression were induced. The plates were incubated overnight and inspected for colony growth. If the cyclase and effector combination give rise to a growth defect or kill the host cell, fewer colonies are observed compared to controls. We used Cst mCpol protein as the cyclase with either its cognate CARF-TM effector or NucC, a previously characterised dsDNA-endonuclease associated with CBASS and type III CRISPR defence [26,27]. NucC is activated by cA_3_ and was included as a control to detect cA_3_ production. In this experiment, mCpol was expressed under control of the T7 promoter (IPTG inducible), while NucC or CARF-TM were expressed under control of the arabinose-inducible pBAD promoter. As expected, expression of NucC with the catalytically inactive mCpol resulted in efficient transformation, as NucC is inactive in the absence of cA_3_ (Fig 3A). In contrast, expression of the CARF-TM effector alone resulted in toxicity and therefore no viable colonies in the absence of mCpol. When the NucC plasmid was transformed into cells expressing Cst mCpol, no transformants were observed, consistent with activation of the nuclease by cA_3_. In contrast, the toxicity of the CARF-TM effector was moderated with co-expressed mCpol. Truncation of the CARF-TM effector to remove the predicted transmembrane domain completely abolished activity, demonstrating that the transmembrane (TM) domain is essential. Expression of the inactive D63N, D64N variant of Cst mCpol did not alleviate the toxicity of CARF-TM, confirming the requirement for cA_3_ synthesis.

We also performed the plasmid challenge assay using the previously characterised *Vibrio metoecus* (Vme) type III CRISPR-Cas Cmr complex instead of the type II Panoptes mCpol as the cyclase component (S3 Fig). When activated, the Vme Cmr complex produces 3′3′3-cA_3_ with a trace of cA_4_, and its cognate effector is the NucC nuclease [26]. The Vme Cmr complex was able to fulfil the same role as Cst mCpol, reducing the growth defect imparted by CARF-TM. This confirmed that 3′3′3′-cA_3_ was the relevant signalling molecule for type II Panoptes.

Overall, these data establish Panoptes type II as a defence system that signals via cA_3_, using a signalling logic similar to Panoptes type I. To explore this further, we challenged the defence system with two different proteins that are known to bind or degrade cA_3_: the phage sponge protein Acb2 [14] and the cA_3_-degrading ring nuclease Crn4 [28]. Expression of either of these proteins from a plasmid resulted in the activation of Panoptes type II, reflected in cell toxicity when the CARF-TM effector was induced by arabinose (Fig 3B). This mimics the situation where a phage utilising a sponge or ring nuclease to subvert CBASS or type III CRISPR defence would activate Panoptes type II defence.

To extend this analysis, we turned to the BASEL collection of *E. coli* phages [29]. Some phages in this collection encode the anti-CBASS phosphodiesterase Acb1 [30], which degrades a range of cyclic di- and tri-nucleotides including cA_3_. Doherty and colleagues demonstrated that the *acb1* gene is disrupted in phage Bas43, but intact in closely related phages such as Bas35-37 of the Tevenvirinae family [23]. They showed that disruption of *acb1* correlated with resistance to the type I Panoptes system. We recapitulated this analysis using the type II Panoptes system, observing a clear resistance phenotype for Bas43, where the cA_3_-degrading Acb1 protein is non-functional (Fig 3C). Bas35-37, with functional Acb1, were all sensitive to type II Panoptes defence, resulting in fewer and smaller plaques (Fig 3D and 3E), consistent with the data obtained from plasmid challenge experiments. In the course of these experiments, we isolated a number of escaper mutants for phage Bas35 which generated larger plaques in the presence of active type II Panoptes defence. Sequencing of the PCR-amplified *acb1* region in these escapers revealed single-nucleotide mutations predicted to deactivate the *acb1* gene (Fig 3F). Although the complete phage genomes were not sequenced, these mutations in *acb1* are likely to be responsible for the observed phenotypes.

In sum, the in vivo data all point to a key role for cA_3_ depletion in the activation of type II Panoptes. Phage challenged by Panoptes defence escape by deactivating the genes encoding cA_3_-depletion proteins. This, of course, could render them sensitive to CBASS and type III CRISPR defence.

### The CARF-TM effector binds cA_3_

**Fig 4:**
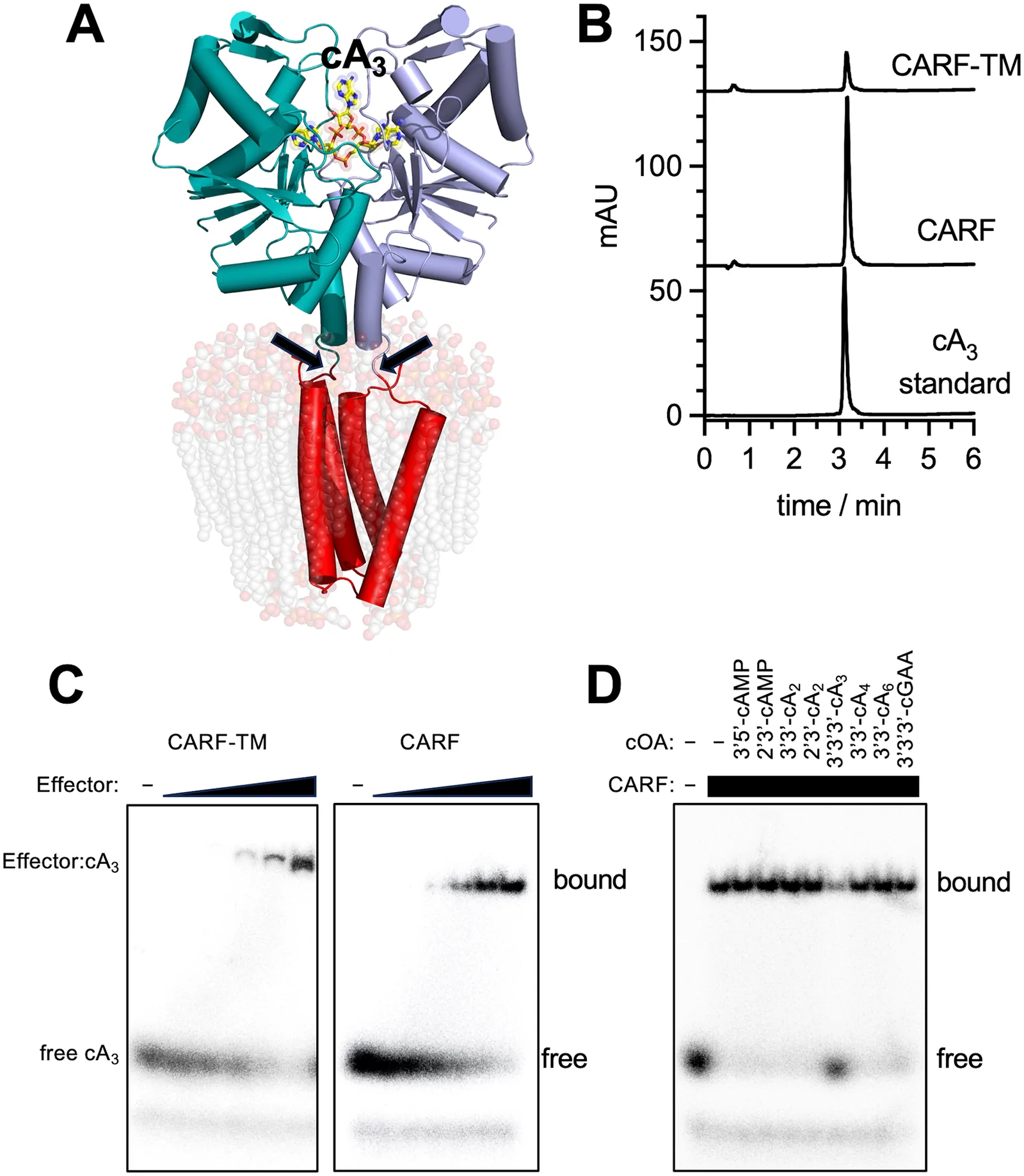
CARF-TM model structure and cA_3_ binding. **A.** Alphafold3 model of CARF-TM in complex with cA_3_, including POPG molecules to simulate the membrane bilayer. The position mutated to generate the truncated CARF protein is shown by black arrows. **B**. HPLC analysis of the supernatant of heat-denatured CARF-TM and CARF confirmed the presence of cA_3_ in the purified proteins. The data underlying this panel can be found in S1 Data. **C.** Radiolabelled cA_3_ (100 nM) was incubated with increasing CARF-TM (125 nM–2 µM dimer) or CARF (200 nM–7 µM dimer) and analysed by EMSA. **D.** The CARF: [^32^P]-cA_3_ complex was challenged with a 100-fold excess of a range of cyclic nucleotides, as indicated. Only 3′3′3′-cA_3_ was able to displace the radiolabelled bound ligand.

Modelling with Alphafold3 [31] strongly supported a dimeric structure for the CARF-TM effector (Fig 4A), consistent with the dimeric organisation of many other CRISPR-associated CARF effectors [22]. cA_3_ was modelled bound at the interface between the two subunits. To express and purify recombinant CARF-TM, we had to co-express the protein with mCpol, generating cA_3_ to keep the effector in an inactive state. Under these conditions, CARF-TM with a non-cleavable N-terminal polyhistidine tag could be purified to homogeneity via immobilised metal affinity and size exclusion chromatography in the presence of the detergent DDM (S4A and S4B Fig). We also purified a truncated form of the effector (CARF), where the TM domain was replaced with a non-cleavable C-terminal hexahistidine tag. The truncated protein also required the presence of the cyclase for efficient purification. We observed two bands in the final purified sample for full-length CARF-TM and for the truncated CARF protein (S4C and S4D Fig); both were confirmed by mass spectrometry to be the effector. An elevated absorbance at 260 nm for both the full-length and truncated proteins was suggestive of the presence of nucleic acid. We extracted nucleotides from both proteins and analysed them by HPLC, confirming the presence of cA_3_ in roughly equimolar amounts to the protein dimer (Fig 4B).

These observations demonstrated that cA_3_ remains bound to both the full-length and truncated effector during purification, suggesting a low dissociation constant for cA_3_ binding. To explore ligand specificity, we investigated binding of ^32^P-radiolabelled cA_3_ by electrophoretic mobility shift assay (EMSA). At high concentrations of CARF-TM or CARF domain (approximately 1 µM dimer), a single retarded species consistent with cA_3_ binding became visible (Fig 4C). This high apparent dissociation constant likely reflects the fact that both proteins were already bound to unlabelled cA_3_, requiring displacement by competition with the radioactive species. The retarded radioactive cA_3_ moiety was abolished when a 100-fold molar excess of cold 3′3′3′-cA_3_ was added, but binding was unaffected by a range of other cyclic nucleotide species (Fig 4D). Overall, these data demonstrate that both the CARF-TM effector and its truncated, soluble CARF domain bind cA_3_ specifically and with a high enough affinity to persist during protein purification. However, binding is not irreversible and is subject to competition by free cA_3_ in vitro.

### The soluble CARF variant multimerises when cA_3_ is not bound

We next investigated whether the CARF effector underwent any conformational changes upon loss of cA_3_, mimicking the effect of a cA_3_-scavenging phage protein. Although we could purify small quantities of the full-length CARF-TM effector bound to cA_3_, plasmid challenge experiments indicated that the presence of N- or C-terminal tags abolished function in vivo (S5 Fig). We therefore focussed on the truncated CARF version of the effector for further in vitro studies, cognisant of the limitations inherent in this approach. To deplete cA_3_ from the CARF protein, we incubated the purified protein with increasing concentrations of the ring nuclease Crn4 [32]. HPLC analysis of the nucleotide species present following Crn4 incubation confirmed that a significant proportion of the cA_3_ present was converted to smaller products, confirming that the ring nuclease can compete with the CARF-TM effector for cA_3_ ligands in vitro (S6 Fig). This is consistent with the competition observed by free cA_3_ in the EMSA experiments and with the in vivo data that demonstrate Panoptes activation by Crn4 (Fig 3).

**Fig 5:**
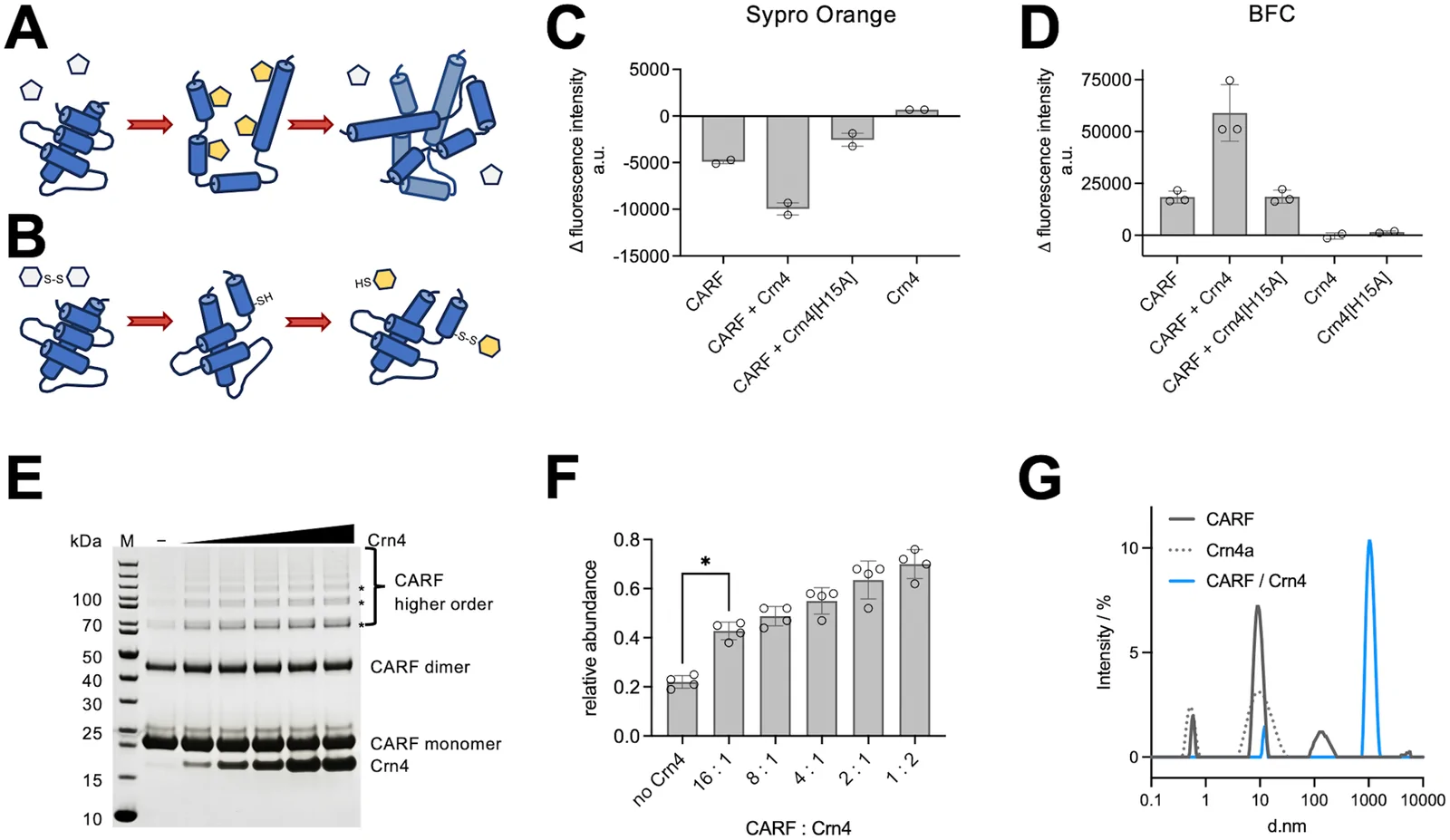
Removal of cA_3_ results in oligomerisation of the CARF protein. **A.** Cartoon of Sypro Orange binding to exposed apolar patches upon protein unfolding followed by occlusion of hydrophobic binding sites through protein aggregation. **B**. The BFC dimer becomes fluorescent upon cleavage of its disulfide bridge by cysteine residues. **C, D.** Difference in fluorescence intensity after 50 min incubation of CARF with Crn4 wild type or catalytically inactive H15A variant. The greatest change in fluorescence was observed for CARF with wild-type Crn4. Removal of cA_3_ appears to remove available hydrophobic residues (C, Sypro Orange, mean and range of duplicates shown) but expose Cys residues (D, BFC, mean and standard deviation of triplicates shown). **E, F**. Chemical cross-linking of CARF in the presence of increasing amounts of Crn4. CARF and Crn4 were incubated for 30 min before separating on SDS-PAGE. CARF oligomers labelled with an asterisk in E were quantified by densitometry relative to the CARF dimer band. Statistical analysis in F was performed with Prism 10 (Graphpad) using the Mann–Whitney test (two-tailed). *: *p* value 0.0286. **G.** DLS analysis of CARF after incubation with Crn4. The data underlying Fig 5C, 5D, 5F and 5G can be found in S1 Data.

To examine the effect of cA_3_ removal on the effector, we probed structural changes using the fluorogenic dyes Sypro Orange and BODIPY FL L-cystine (BFC). Both dyes are typically used with thermal shift assays; however, we conducted isothermal assays following changes in fluorescence after addition of Crn4 to the cA_3_:CARF complex. The fluorescence intensity of Sypro Orange increases upon binding to hydrophobic regions, which are normally buried in a folded protein but become exposed when the protein denatures and in turn become hidden when an unfolded protein aggregates (Fig 5A). The fluorescence of BFC in its disulfide dimer form is quenched. Cleavage by free thiols, such as exposed cysteine residues, releases the monomeric, fluorescent dye (Fig 5B). Unfolding and/or increased protein chain mobility can expose buried cysteine residues leading to an increase in fluorescence. We observed a decrease in Sypro Orange fluorescence intensity upon Crn4 treatment, consistent with burial of hydrophobic patches upon ligand removal (Fig 5C). For the BFC dye, isothermal incubation of the CARF protein with Crn4 resulted in a significantly larger increase in fluorescence than in the absence of Crn4 or with the catalytically inactive Crn4 [H15A] variant [28] (Fig 5D).

The effect of cA_3_ removal on the quaternary structure of the CARF protein was also tested using chemical cross-linking and SDS-PAGE (Fig 5E). In the absence of Crn4, the CARF protein was observed primarily as monomeric and dimeric after crosslinking with very minor amounts of larger species. With increasing amounts of Crn4, progressively higher amounts of large (>dimeric) cross-linked species were observed (Fig 5F). This assay benefited from the unusual amino acid composition of Crn4, which lacks lysine residues and thus cannot cross-link [32]. We also investigated the removal of cA_3_ from the CARF domain by dynamic light scattering (DLS). Notably, a precipitate formed when Crn4 was incubated with the CARF domain. As the samples were centrifuged, the precipitate was removed prior to DLS analysis. An approximately 100-fold increase in size of the remaining soluble CARF protein was detected upon cA_3_-depletion (Fig 5G).

Together, these observations suggested that cA_3_ removal results in structural perturbations of the CARF dimer, leading to unfolding or aggregation in vitro. The absence of the TM domain in these studies is a significant caveat, however. Given the difficulty in studying the full-length CARF-TM protein in vitro, we turned to in vivo analyses.

### The CARF-TM effector causes growth arrest in the absence of cA_3_

**Fig 6:**
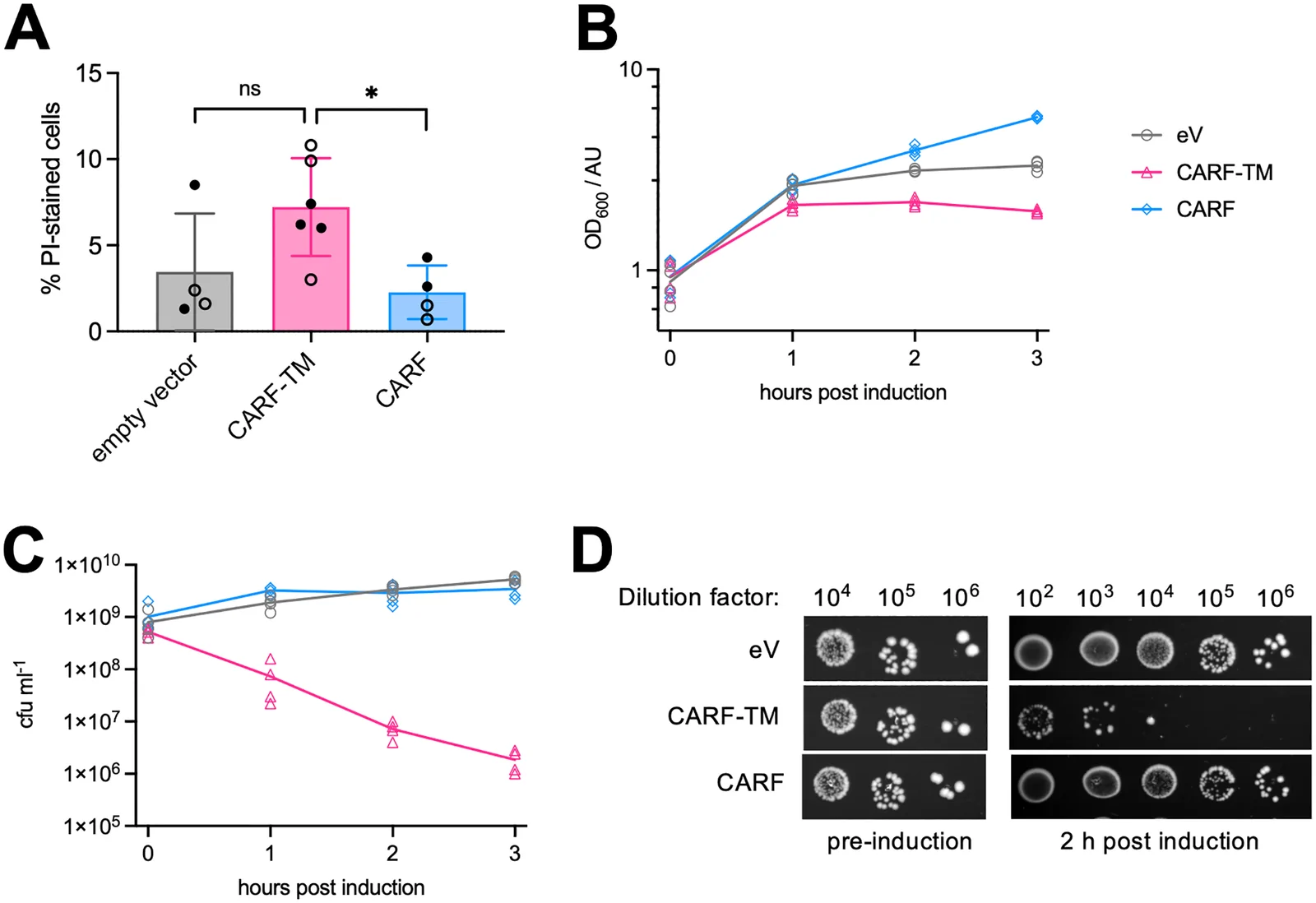
The CARF-TM effector causes growth arrest in the absence of cA_3_. **A**. Live/dead staining of cells expressing Cst CARF-TM, CARF or empty vector. Samples from 1 h (open circles) or 2 h (closed circles) after induction of the effector protein were analysed by confocal fluorescence microscopy. There was no significant difference in the percentage of PI-stained cells between strains expressing CARF-TM or those lacking the effector (*p* value 0.11, two-tailed Mann–Whitney test); however, cells expressing the truncated effector CARF had a lower percentage of PI-staining than those expressing full length CARF-TM (*p* value 0.019). Data show the mean and standard deviation from three independent experiments. More than 1,000 cells were counted in total for each strain. **B.** Optical density of cultures after induction of protein expression. **C, D**. Cells expressing CARF-TM lost their ability to proliferate as determined by microdilution assay. Data for panels B, C show the individual values of four biological replicates as symbols with a line representing the mean. One representative example of the microdilution assay is shown in D. eV: empty vector. The data underlying Fig 6A, 6B and 6C can be found in S1 Data.

We set out to monitor changes in cell viability upon expression of CARF-TM using microscopy. The full-length, untagged CARF-TM was expressed from the T7 promoter in pEHisV5TEV [25], allowing induction of expression using IPTG. We omitted the *mcpol* gene from these strains, so that the effector would be in the active *apo* form when expressed. *E. coli* harbouring CARF-TM, truncated CARF or the empty vector were grown to mid-log phase before induction with 0.1 mM IPTG. Cells collected 1–2 h after induction were stained with Syto9 and propidium iodide (PI) [33]. Both are nucleic acid staining fluorescent dyes; Syto9 is a membrane-permeable dye that will stain live and dead cells, whereas PI can only enter cells with significantly damaged membranes. PI has a higher affinity for nucleic acids than Syto9 and tends to displace Syto9 from the nucleic acid given a high enough concentration. We determined the percentage of PI-dyed cells using fluorescent confocal microscopy (Figs 6A and S7). We observed minor differences between CARF-TM-expressing (7.2% PI-stained) or non-expressing cells (3.5% PI-stained for empty vector, 2.3% for CARF). This suggested that CARF-TM is not a fast-acting bactericidal agent when expressed in *E. coli*, but rather induces a growth arrest phenotype.

To follow on from the live/dead staining, we determined the cell density and culturability of the three strains for the first 3 h after IPTG induction. The optical density initially increased for all three strains, levelling off for the CARF-TM-expressing strain after 2 h, consistent with growth arrest upon effector expression (Fig 6B). The growth of cells with the empty vector control slowed significantly on IPTG addition—a phenomenon that was consistently observed and which may relate to the burden IPTG places on cells in a context-dependent manner [34]. We therefore investigated the equivalent growth curves using the Aaf Panoptes system under the control of an arabinose promoter, confirming that Aaf CARF-TM, but not truncated CARF, induced growth arrest in the absence of mCpol, with no growth defect in the presence of mCpol (S8 Fig).

Microdilution and colony counting assays for the Cst effector demonstrated that whilst the strains with empty vector or expressing the CARF domain yielded a similar number of colony-forming units (cfu), those for the CARF-TM-expressing strain dropped by 2–3 orders of magnitude (Fig 6C and 6D). Combined with the data from PI staining, this suggests cells experience growth arrest when the CARF-TM effector is activated. While the cells don’t appear to lyse, as observed for type I Panoptes [23], they may still be functionally “dead” to all intents and purposes.

### Expression of apo CARF-TM may alter cell permeability in *E. coli*

**Fig 7:**
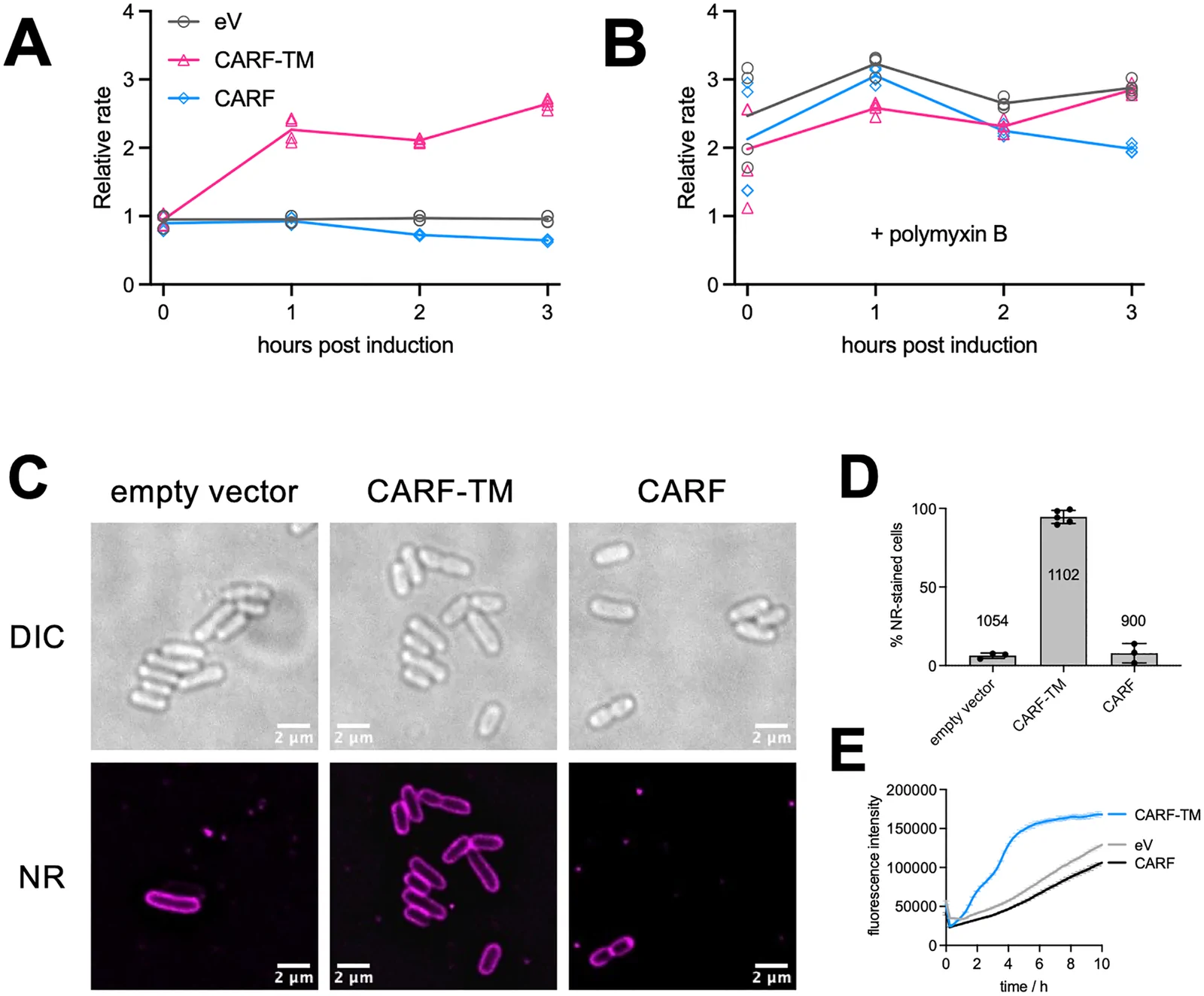
Expression of apo CARF-TM perturbs membrane permeability. **A, B.** Initial rates of resazurin reduction in the absence (A) and presence (B) of polymyxin B in cells expressing the Cst CARF-TM or CARF effectors, relative to empty vector (eV). Data show individual values of four biological replicates with a line connecting the means. **C.** Differential interference contrast (DIC) and fluorescence microscopy of *E.* coli cells expressing CARF-TM or CARF. Cells were collected one hour after addition of IPTG to induce expression of the effector protein and stained with Nile Red (NR). Membranes of *E. coli* expressing CARF-TM were readily stained with Nile Red, whereas most cells expressing the truncated CARF domain or carrying the empty vector remained unstained. The data underlying this panel can be found in DOI: https://doi.org/10.17630/ac5e296c-fff0-4c63-b218-f13d08adb865. **D.** Percentage of NR-stained cells between 1 and 2 h post-induction. The total number of cells for each strain is indicated in the graph. Data are from at least two independent experiments. **E.** NR-fluorescence is increased upon expression of CARF-TM relative to CARF or empty vector in liquid culture. The data underlying Fig 7A, 7B, 7D and 7E can be found in S1 Data.

We next assessed the effect of Cst CARF-TM on metabolic changes in response to altered cell permeability by monitoring the cellular reduction of resazurin to resorufin [35] (Fig 7). Before induction, the fluorescent signal was indistinguishable between the three strains (CARF-TM, CARF and eV). By 1 h after induction, CARF-TM-containing cells showed a faster rate of resorufin production compared to control cells (Fig 7A). This could indicate an increase in NAD(P)H levels. However, we observed that the addition of the antibiotic polymyxin B, which permeabilises the outer membrane (OM) of gram-negative cells [36,37], yielded very similar results, though with a faster timescale (Fig 7B). Indeed, there was no additive effect for CARF-TM expression and polymyxin B exposure. These data may indicate that the observed increase in resazurin reduction on both CARF-TM expression and polymyxin B treatment may be a consequence of increases in cell permeability, rather than a simple increase in intracellular NAD(P)H levels.

To explore this further, we investigated cell staining using the lipophilic dye Nile Red (NR) [38]. Cells expressing the apo CARF-TM effector were readily stained by NR (Fig 7C and 7D). Under the same conditions, only a small fraction of cells expressing the CARF domain or containing the empty vector were stained with NR (Fig 7C and 7D). When growing in liquid culture in the presence of NR, cells expressing CARF-TM gave rise to significantly enhanced NR fluorescence over time (Fig 7E). These data support a model where expression of apo CARF-TM in the inner membrane of *E. coli* results in permeabilization of the OM and growth arrest, but not rapid cell death. It should be noted; however, that effects observed on membrane permeabilization are indirect, with the significant caveat that the system is being studied in a heterologous host.

## Discussion

Here, we have shown that type II Panoptes systems with CARF-TM effectors, which constitute around 40% of total instances [24], signal using cyclic triadenylate (cA_3_). Like type I Panoptes, the type II system can provide effective antiphage defence independently when phages interrupt cyclic nucleotide signalling. This defence is easily circumvented by phages; however, simply by deleting or disrupting anti-defence genes such as *acb1*. Direct demonstration of a guard function for Panoptes has not yet been demonstrated, but is an attractive proposition when considering the range of defence systems, including CBASS and CRISPR, that respond to phage infection by synthesising cyclic nucleotides to activate effectors.

To work as a guard system, Panoptes must generate a cyclic nucleotide that is not used by the other defence systems present, but which is targeted by phage for sequestration or degradation. Type II Panoptes could co-exist and cooperate with the majority of type III CRISPR systems, which signal via cA_4_ or cA_6_ [19], and with the majority of CBASS systems, which utilise a wide range of cyclic di- or tri-nucleotides [39]. A comprehensive analysis of the distribution and gene linkage of mCpol revealed that about 50% of Panoptes operons are found in genomes that also encode CBASS defence, occurring in the same gene neighbourhood in half of these instances [24]. A significant enrichment of type III CRISPR systems in Panoptes-containing genomes was also noted [24]. The *C. stagnale* genome, harbouring a type II Panoptes system, also encodes a type II CBASS system with a Cap4 effector closely related to enzymes that are activated by 2′3′3′-cA_3_ and 3′3′3′-cAAG [40,41]. Likewise, the Sph Panoptes system is situated adjacent to a type II CBASS system with a Cap4 effector. Both of these Panoptes systems could thus be using 3′3′3′-cA_3_ as a decoy for the CBASS signalling molecules. On the other hand, the Cst genome also encodes two type III CRISPR systems which appear to function via cA_4_ signalling, with Csx1 and Can2 effectors [19] whilst the Aaf genome [42] lacks any CBASS systems but does have type III CRISPR. In these cases, the deployment of a cA_3_ decoy molecule to guard type III CRISPR defence could prove effective.

The use of cA_3_ as a guard molecule in turn suggests that phage sequester or degrade this molecule as a side effect of their efforts to circumvent CBASS or CRISPR defence. Some phage-encoded Acb2 sponge proteins bind cA_3_ [14], while the phosphodiesterases Acb1 [30] and Crn4 [28] degrade cA_3_ in vitro. We confirmed functional activation of the type II Panoptes system by both Acb2 and Crn4 in vivo in a plasmid challenge assay (Fig 3). Furthermore, phage from the BASEL collection that express Acb1 (e.g., Bas35) are subject to restriction by Panoptes type II, while a closely related phage (Bas43) that has disrupted the *acb1* gene escapes both type I and II Panoptes defence ([23] and this study). The latter observations highlight the quandary faced by phage, where expression of a non-specific cyclic nucleotide depletor like Acb1 or Acb2 opens the door to Panoptes defence. We observed phage Bas35 mutating the *acb1* gene to escape from Panoptes, but this would render the phage more susceptible to CBASS or CRISPR defence. An alternative strategy for phages would be to utilise a sponge or phosphodiesterase highly specific for only one cyclic nucleotide species. This could avoid Panoptes activation, but would provide protection against a much narrower range of cyclic-nucleotide generating defence systems.

To function as a guard system, Panoptes must be sensitive to changes in the concentration of the cyclic nucleotide, in this case cA_3_, which keeps the effector inactive. The low turnover rate shared by three type II mCpols, at around 1 h^−1^ in vitro, suggests a low cellular concentration of cyclic nucleotide in vivo. Dissociation constants for cyclic nucleotide ligands by CARF family proteins are typically in the nanomolar range [43–45], and consistent with this we observe co-purification of cA_3_ with the CARF-TM protein. Nonetheless, bound cA_3_ exists in equilibrium with the free species, allowing competition with bulk cA_3_ in vitro and with sequestering/degrading proteins in vitro and in vivo. In such a scenario, rather few molecules of cA_3_ in the cell could be sufficient to keep the Panoptes effector in an inactive state, while providing a sensitive readout of phage interference.

Activation of type I Panoptes with a 2TMβ effector results in inner membrane (IM) collapse and cell death [23]. For type II Panoptes, all our observations are suggestive of permeabilization of the OM upon expression of CARF-TM in apo form (mimicking the situation where a phage infection has disrupted cA_3_ signalling) and growth arrest, but not cell lysis. It is likely that the CARF-TM protein is situated in the IM with the CARF domain facing the cytoplasm where cA_3_ is synthesised. The inner and outer membranes are of course functionally linked [46], as OM phospholipids, lipopolysaccharides and outer membrane proteins (OMPs) [47] are synthesised in the cytoplasm and transported through the IM to the OM [48]. Changes in any of the three OM components have the capacity to cause OM disruptions [49]. Disruption or depolarisation of the IM can thus have knock-on effects on the OM. Further investigation will be required to differentiate causality and indirect effects, ideally using a cognate host and genomic expression of the Panoptes system from native promoters.

Rapid advances in recent years have resulted in the identification of hundreds of prokaryotic antiviral defence systems [1,2]. The “average” bacterium encodes over 7 different systems, but it has not been clear whether they often function synergistically to provide antiviral immunity. Guard systems such as Panoptes, and the Hailong system that operates via a decoy ssDNA molecule [50], are an intriguing facet of bacterial anti-phage immunity, providing the opportunity for synergistic interactions between different defence components [24]. Undoubtedly, further examples will be uncovered in the coming years.

## Materials and methods

### Cloning

Enzymes were purchased from Thermo Scientific or New England Biolabs and used according to manufacturer’s instructions. Oligonucleotides and codon-optimised synthetic genes were obtained from Integrated DNA Technologies (Coralville, Iowa, USA). All constructs were verified by sequencing (Eurofins Genomics, DE).

The mCpol-encoding genes from *Cylindrospermum stagnale* PCC 7417 (*Cst*; WP_015209627.1), *Sphingomonas* sp. (Sph; WP_230771556.1), and *Ahniella affigens* D13 (*Aaf*; WP_106889607.1) were cloned into pEHisV5TEV [25] for expression with a cleavable N-terminal His_8_-tag. Active site residue variants were obtained by primer-directed mutagenesis [51]. Cst CARF-TM (WP_015209626.1) and Cst mCpol were cloned into a modified pRSFDuet-based plasmid by restriction assembly to yield an N-terminal His6-TEV-CARF-TM and an untagged Cst mCpol. The expression construct for soluble *Cst* CARF was obtained by PCR amplification of the CARF domain, followed by restriction digest and ligation into 5′-NdeI and 3′-XhoI sites of pEV5hisTEV [25]. This allows expression of *Cst* CARF with a native N-terminus and the LEHHHHHH affinity tag following residue T207 of *Cst* CARF. The *Cst* mCpol gene was cloned into the 5′-NdeI, 3′-XhoI sites of pRAT [52] to allow co-expression of the native *Cst* mCpol from an arabinose-inducible promoter with IPTG-inducible *Cst* CARF-CHis.

For plasmid challenge assays, *Cst* mCpol was cloned into the 5′-NdeI, 3′-XhoI sites of pACE (Geneva Biotech, Genève, CH) and *Cst* CARF-TM was inserted into MCS1 of pRATDuet [13] to allow induction with IPTG and l-arabinose, respectively. *Cst* CARF was obtained by primer-directed mutagenesis to introduce a stop codon after residue T207 of the native sequence. The ring nuclease Crn4 from *Actinomyces procaprae* [28] and the cyclic nucleotide sponge Acb2 from *Pseudomonas* phage PaMx33 [14] were each cloned into MCS2 of pRATDuet alongside *Cst* CARF-TM in MCS1. pRATDuet-NucC has been previously described [53].

The *Aaf* type II Panoptes system (WP_106889607.1 and WP_106889608.1) was used for phage assays. Synthetic versions of the wild-type *Aaf*-mCpol-CARFTM locus and *Aaf*-CARFTM alone were ligated into pRFSara. This plasmid was derived from pRSFDuet-1 (Novagen, Merck-Millipore) by replacing the *lacI* and T7 promoter region with the *araC* and pBAD promoter region from pBADHisTEV [54] whilst removing multiple-cloning-site 1 [52]. The transmembrane domain of CARF-TM was removed by mutating the L212 codon to a stop codon.

Untagged *Cst* CARF-TM and CARF constructs used in microscopy and in vivo fluorescence studies were obtained through restriction digest and ligation. Full-length *Cst* CARF-TM was ligated into the 5′-NdeI, 3′-XhoI sites of pEHisV5TEV, which removed the N-terminal affinity tag, to give pE-*Cst* CARF-TM. The truncated *Cst* CARF was obtained by primer-directed mutagenesis to introduce a stop codon after residue T207 of the native sequence to give pE-*Cst* CARF.

### Protein purification

#### mCpol.

*Cst*, *Sph* and *Aaf* mCpol were expressed in C43 (DE3) *E. coli*. Two litres of culture were induced with 0.4 mM isopropyl-β-D-1-thiogalactoside (IPTG) at an OD_600_ of ~0.8 and grown overnight at 16 °C. Cells were harvested (4,000 rpm, 10 min, 4 °C, Avanti JXN-26, Beckman Coulter, JLA-8.1 rotor) and resuspended in lysis buffer (50 mM Tris-HCl pH 7.5, 0.5 M NaCl, 10 mM imidazole,10% glycerol, lysozyme, protease inhibitors (cOmplete EDTA-free protease inhibitor, Roche) and lysed by sonicating six times 1 min on ice with 1 min rest intervals (Soniprep 150, MSE). Lysates were clarified by ultracentrifugation (40,000 rpm, 30 min, 4 °C Optima L-90K, 70Ti rotor) and filtered. Proteins were purified with an immobilised metal affinity chromatography (IMAC) column (HisTrapFF crude, Cytiva), washed with 20 column volumes (CV) of buffer containing 50 mM Tris-HCl pH 7.5, 0.5 M NaCl, 30 mM imidazole and 10% glycerol, followed by a step elution with buffer containing 50 mM Tris-HCl pH 7.5, 0.5 M NaCl, 0.5 M imidazole and 10% glycerol on an NGC Chromatography System (Biorad). Protein-containing fractions were concentrated and the affinity tag was removed by incubation of protein with Tobacco Etch Virus (TEV) protease (10:1) overnight at room temperature. Cleaved proteins were isolated from TEV by repeating the IMAC step and the unbound fraction collected. Size exclusion chromatography (Superdex 200 16/60, Cytiva) was used to further purify the proteins, with proteins eluted isocratically with buffer containing 20 mM Tris-HCl pH 7.5, 250 mM NaCl. The proteins were concentrated using a centrifugal concentrator (Amicon Ultra-15, Merck), aliquoted and stored frozen at −70 °C. Examples of purified proteins are shown in S9 Fig.

#### Cst CARF-TM and CARF soluble domain.

The Cst CARF-TM protein was transformed into competent *E. coli* BL21(DE3)pLysS cells. The protein was expressed by growing cells to an OD_600_ of 0.6–0.8 in LB broth at 37 °C and inducing with 0.5 mM IPTG subsequent to a 4 °C cold shock for 20 min. Expression was then allowed to continue overnight at 16 °C. Cells were harvested by centrifugation at 6,240*g* for 15 min. Cells were resuspended in a buffer of 50 mM HEPES pH 7.3, 300 mM NaCl and supplemented with cOmplete EDTA-free protease inhibitor (Roche). Cells were lysed by 3 passes through a continuous flow cell disruptor (Constant Systems) at 30 kpsi. Insoluble cellular material was pelleted by centrifugation at 20,000*g* for 15 min, the resulting supernatant was centrifuged at 110,000*g* for 45 min to pellet membrane material. Membranes were homogenised in a solution of 50 mM HEPES pH 7.3, 300 mM NaCl, 20 mM Imidazole, 20 mM DDM and 0.5 mM Tris-(2-Carboxyethyl)phosphine (TCEP), then allowed to solubilise for 4 h at 4 °C. Insoluble material was subsequently removed by centrifugation at 110,000*g* for 45 min. Cst CARF-TM was purified by TALON affinity chromatography using a wash of 20–70 mM imidazole followed by elution at 500 mM imidazole. Size exclusion chromatography (SEC, Superdex 200, 10/300, Cytiva) in a buffer of 50 mM HEPES pH 7.3, 150 mM NaCl, 0.5mM DDM, 0.5 mM TCEP was carried out as an additional purification step.

The *Cst* CARF construct was co-transformed in *E. coli* C43(DE3) alongside untagged *Cst* mCpol in pRAT [52] and a single colony used to inoculate LB medium containing 50 µg/ml kanamycin and 12 µg/ml tetracycline for overnight growth at 37 °C. The overnight culture was diluted 20-fold into fresh LB medium containing antibiotics and incubated at 37 °C with shaking until the OD_600_ reached 0.6–0.8. *Cst* CARF production was induced with 0.1 mM IPTG and mCpol with 0.1% l-arabinose. Incubation was continued at 28 °C for 4 h, when cells were collected and the pellet stored at −20 °C. Cells were resuspended in lysis buffer (50 mM Tris, 1 M NaCl, 20 mM imidazole, 10% glycerol, pH 7.7), lysed by sonication and the lysate was cleared by centrifugation (Ti70 rotor, 40,000 rpm, 45 min, 4 °C). *Cst* CARF was pulled down by IMAC (HisTrap crude, Cytiva), washed with 4% elution buffer (lysis buffer containing 0.5 M imidazole) and eluted in a gradient to 100% elution buffer. Protein-containing fractions were pooled, concentrated using an Amicon Ultra-15 (Merck) spin filter with 10 kDa MWCO, and further purified by SEC (HiLoad 16/600 Superdex 200 gp, Cytiva) in 20 mM HEPES, 250 mM NaCl, 10% glycerol, pH 7.5. Protein-containing fractions were pooled, concentrated as before, flash-frozen and stored at −70 °C. Protein concentrations were determined by UV quantitation (NanoDrop 2000, Thermo Scientific) using calculated extinction coefficients (Benchling Biology Software, 2024).

#### Crn4 ring nuclease.

Production and purification of Crn4 (Crn4a, WP_136192672, from *Actinomyces procaprae*) has been described previously [28].

### mCpol activity

Nucleotide cyclase activity was assayed in 12.5 mM Tris-HCl, pH 8.0, 125 mM NaCl, 10 mM MgCl_2_ containing 500 µM ATP or ATP analogue and 5–25 µM (dimer) *Cst* mCpol wild type or mutant. The reaction was incubated for 3 h at 37 °C, unless stated otherwise. Enzymes were removed by ultracentrifugation (3 kDa MWCO) or by heat deactivation (95 °C for 5 min) followed by centrifugation (16,000*g*, 10 min, 4 °C). Reaction products were analysed by HPLC (Dionex UltiMate 3000 equipped with single-wavelength UV detector, Thermo Scientific) as follows. Compounds from *Cst* mCpol reactions were separated on a Synergi Fusion RP column (2.5 µm, 2 × 50 mm, Phenomenex) at 0.3 ml min^−1^ and 40 °C column temperature with a linear gradient of 2%–50% methanol against 20 mM ammonium bicarbonate over 4.8 and 0.2 min gradient delay. Elution was monitored by UV at 260 nm. Data were analysed using Chromeleon and visualised in Prism 10 (GraphPad). Cyclic nucleotides were identified by comparison to and co-injection with synthetic standards (Biolog, DE) or by mass spectrometry.

Selected samples were analysed by liquid chromatography-mass spectrometry on an LCQ Fleet mass spectrometer coupled to an Ultimate 3000 UHPLC module (Thermo Scientific) using the same liquid chromatography conditions as above. Electrospray ionisation was monitored in positive ion mode without fragmentation. Data were analysed with Excalibur Qual Browser software and visualised in Prism 10.

### cA_3_ production rate

cA_3_ production by mCpol enzymes was determined using the EnzChek Pyrophosphate Assay kit (Invitrogen, Fisher Scientific). Reactions contained 12.5 mM Tris-HCl, pH 8.0, 100 mM NaCl, 10 mM MgCl_2_, 0.2 mM 2-amino-6-mercapto-7-methylpurine ribonucleoside (MESG), 1 U ml^−1^ purine nucleoside phosphorylase, 0.1 U ml^−1^ pyrophosphatase, 0.5 mM ATP (Fermentas, Fisher Scientific) and 2.5–5 µM mCpol dimer. Background hydrolysis was determined by omitting mCpol. Pyrophosphate (1–10 µM) in the reaction mixture without ATP and mCpol were used to obtain the standard curve. The reaction was started by the addition of ATP or pyrophosphate standards and the absorption at 360 nm was monitored at 37 °C using a FluoStar Omega (BMG Labtech) plate reader. Data analysis was performed in Prism 10 (Graphpad). Rates were determined by linear regression, corrected for enzyme concentration with the assumption that for each cA_3_ molecule, three molecules of pyrophosphate are generated, and that non-production (pyro)phosphate release is negligible. The reported rate was the mean from three independent experiments.

### Dynamic light scattering

DLS measurements were performed with the Zetasizer Nano S90 (Malvern) instrument. Samples contained 60–100 µM protein dimer in 20 mM Tris-HCl, 75 mM NaCl, 10 mM MgCl_2_ and 0.1% DDM as required. After centrifugation at 12,000*g* for 10 min at room temperature, the sample was centrifuged and loaded into a quartz cuvette (ZMV1012). Measurements were performed at 25 °C with three measurements of 13 runs.

### Electrophoretic mobility shift assay

[α-^32^P]-Radiolabelled cA_3_ was prepared using 45 µM *Cst* mCpol dimer in 12.5 mM Tris-HCl, pH 8.0, 100 mM NaCl, 10 mM MgCl_2_ and 300 µM [^32^P]-ATP (0.15 MBq). The reaction was incubated at 37 °C for 3 h, protein was removed by phenol-chloroform extraction and excess phenol was removed by an additional chloroform extraction step. A typical EMSA assay contained 100 nM [α-^32^P]-cA_3_ and 0–4 µM His_6_-CARF-TM monomer in 30 mM HEPES, pH 7.5, 90 mM NaCl, 10 mM MgCl_2_, 10% glycerol, 0.4 mM DDM or 100 nM [α-^32^P]-cA_3_ and 0–14 µM CARF-CHis_6_ monomer in 12.5 mM Tris-HCl, pH 8.0, 200 mM NaCl, 10 mM MgCl_2_, 10% glycerol, unless stated otherwise. The mixture was incubated at room temperature for 30 min before addition of G-250 Native Gel Sample Loading Buffer (Invitrogen) to 0.005% final concentration. Samples were immediately loaded onto a pre-run 6% acrylamide gel (29:1 acrylamide:bis-acrylamide). Radiolabelled material was separated for 2 h at 200 V in 1× TBE buffer and visualised by exposure to a phosphor storage screen and phosphorimaging (Cytiva).

### Protein crosslinking

The cross-linking reaction was carried out in 20 mM HEPES, pH 7.5, 250 mM NaCl, 10% glycerol with 20 µM *Cst* CARF dimer in the absence or presence of 2.5–40 µM Crn4. The reaction was incubated at 37 °C for 20 min. The BS-3 (bis(sulfosuccinimidyl)suberate-d_0_, Thermo Scientific) crosslinker was then added to 0.2 mM final concentration and the mixture was incubated with gentle agitation for 30 min at 20 °C. SDS-PAGE sample loading buffer was added to each reaction and the samples were heated to 95 °C for 2 min before analysis by SDS-PAGE.

### Fluorescence assays

Sypro Orange was purchased as a 5,000× stock in DMSO (Invitrogen) and used at 5X final concentration in 20 mM HEPES, 250 mM NaCl, 10% glycerol, pH 7.5 with 9 µM CARF dimer and 3 µM Crn4 dimer wild type or [H15A] variant. The reaction was preincubated with fluorescence monitoring (ex/em 485/580 nm) at 37 °C in a FluoStar Omega plate reader (BMG Labtech) for 15 min before addition of Crn4. BODIPY FL L-Cystine (BFC, Invitrogen, Fisher Scientific) was dissolved in DMSO and used at 1 µM final concentration in the same buffer as for Sypro Orange with 0.5 µM CARF and 1 µM Crn4. The reaction was preincubated for 3 min at 37 °C before addition of Crn4 with fluorescence monitoring at ex/em 485/520 nm. Data were analysed using Graphpad Prism 10.

### Plasmid challenge assay

The plasmid challenge assay was performed as described in [53] with the following modifications. Competent cells were prepared from individual colonies of *E. coli* C43(DE3) transformed with pACE-*Cst* mCpol, pACE-*Cst* mCpol[D63N/D64N] or pACE empty vector. pRATDuet, pRATDuet-*Cst* CARF-TM, pRATDuet-*Cst* CARF, pRATDuet-*Cst* CARF-TM-Crn4 or pRATDuet-*Cst* CARF-TM-Acb2 were used for transformation. Selective conditions were LB agar containing 100 µg ml^−1^ ampicillin for the acceptor cells only and containing ampicillin and 12 µg ml^−1^ tetracycline for uninduced transformants. *Cst* mCpol and *Cst* CARF-TM ± Crn4 or Acb2 expression was induced with 100 µM IPTG and 0.0125% or 0.05% l-arabinose, respectively. Plates were incubated overnight at 37 °C.

### Phage assays and *Aaf*-Panoptes growth curves

PRSFara constructs containing *Aaf*-CARFTM, truncated *Aaf*-CARF[L212*], wild-type *Aaf*-Panoptes (mCpol-CARFTM), or *Aaf*-Panoptes-ΔTM (mCpol-CARF[L212*]) were transformed into *E. coli* MG1655 alongside an empty vector. Individual colonies were used as biological replicates.

For growth curves, the strains were cultured in LB medium containing 50 µg ml^−1^ kanamycin at 37 °C. Overnight cultures were diluted 100-fold into fresh medium and grown to an OD_600_ of 1.0–1.5.

The cultures were then diluted in triplicate to a final OD_600_ of 0.1 in clear, F-bottom 96-well plates (Greiner) with l-arabinose at a final concentration of 50 µM (CARFTM, CARF) or 0.5 mM (Panoptes) and a total volume of 200 µl. Growth was monitored by reading the absorbance at 600 nm (2× 2 matrix scan, 2 mm width, no pathlength correction) every 20 min and double-orbital shaking at 200 rpm for 15 s before each cycle using a FluoStar Omega (BMG Labtech) plate reader.

For phage assays, Panoptes strains were cultured in LB medium containing 50 µg ml^−1^ kanamycin, 0.5 mM l-arabinose, 10 mM MgCl_2_ and 5 mM CaCl_2_. Strains, grown at 37 °C to early exponential phase, were diluted into soft LB agar, kept at 42 °C, to an OD_600_ of 0.1; the inoculated soft agar (7 ml) was immediately poured onto 20 ml LB agar in 10 × 10 cm square Petri dishes. Phages Bas31, Bas35-37 and Bas43 from the BASEL collection [29] were serially diluted 10-fold into SM buffer (50 mM Tris-HCl, 100 mM NaCl, 10 mM MgSO_4_, pH 7.5); 2.5 µl of each dilution was spotted onto inoculated LB agar plates in duplicate and the plates incubated overnight at 28 °C. The efficiency of plating (EOP) was determined by counting the number of plaques at the lowest dilution factor that provided discernible individual plaques. Where plaque size was too small, the last spot that gave a clear zone in the bacterial lawn was taken to be 100 plaques. The concentration of plaque-forming units was calculated taking dilution factor and applied volume into account.

Escaper phages were isolated by three passages of microdilution assays against MG1655/ Aaf type II Panoptes. Culture conditions were as described above. Three plaques were selected for isolation; plaques 1 and 3 were isolated and sequenced as biological duplicates, giving rise to 1a,b and 3a,b. As expected, the duplicates carried identical mutations of the *acb1* sequence. The *acb1* region was PCR-amplified using MyTaq Red (Bioline) and primers 5′-ggaagcagaaattgcaatagca and 5′-cacatattacagaagctggattgc. PCR products were purified using the Monarch PCR and DNA Clean-up kit (New England Biolabs) according to the manufacturer’s recommendation. PCR primers were used for sequencing by Eurofins Genomics. The sequences of the acb1 gene product from escapers 1a, 1b, 2, 3a and 3b are provided in file S2 Data.

### Microscopy

*E. coli* C43(DE3) was transformed with empty vector, pE-*Cst* CARF-TM or pE-*Cst* CARF. Single colonies were used to inoculate LB containing 50 µg ml^−1^ kanamycin. After culturing overnight at 37 °C with shaking, the cultures were diluted 100-fold into fresh selective medium. Cells were grown at 37 °C to an OD_600_ of 0.5–0.8 and induced with 0.1 mM IPTG. Incubation was continued at 37 °C. Samples (0.5 ml) were taken before induction or 1 h after induction. Cells were collected by centrifugation at room temperature (2 min at 1,500*g*) and the pellet was washed once with PBS buffer. The pellet was resuspended in PBS buffer to an approximate OD_600_ of 5–10. Nile Red (Merck) was added from a 2 mg ml^−1^ stock solution in DMSO to a final concentration of 20 µg ml^−1^ and incubated in a warm environment for 30–45 min. Syto 9 (Invitrogen) and propidium iodide (Merck) were added to 2.5 µM and 20 µg ml^−1^ final concentrations, respectively, and incubated at room temperature for 15–30 min.

Samples (4 µl) were applied to a thin agarose slab (1% agarose in water) on a standard glass microscope slide and allowed to dry for a few minutes before covering with a coverslip. Images were acquired with a fluorescence confocal microscope (DeltaVision Imaging System), connected to a digital camera. The system was configured for both fluorescence and transmitted light DIC imaging. Images were recorded with an Olympus 100×/1.40 oil immersion objective lens, deconvolved using softWoRx Explorer 1.3 and further processed with Fiji image analysis software [55].

### Culturability assay

The same strains as for microscopy were used: *E. coli* C43(DE3) transformed with empty vector, pE-*Cst* CARF-TM or pE-*Cst* CARF. Starter cultures were diluted into fresh LB medium containing 50 µg ml^−1^ kanamycin and incubated at 37 °C with shaking. When the OD_600_ reached 0.8–1.0, cultures were diluted with selective medium to an OD_600_ of 0.5 and induced with 0.1 mM IPTG. Incubation was continued at 37 °C. Samples were taken before induction or 1–3 h after induction. For each sample, the OD_600_ was measured, and a 10-fold serial dilution was prepared that was applied to LB agar plates containing kanamycin but no IPTG. Plates were incubated overnight at 37 °C. Spots with isolated colonies were used to count colony-forming units (cfu), from which the cfu ml^−1^ of the original sample was calculated based on dilution and applied volume (2.5 µl). The results are derived from four biological replicates. Mean and standard deviation were plotted using Prism 10 (Graphpad).

### Resazurin assay

We used the samples obtained from the culturability assay (above). For the resazurin assay, the cell density of each sample was adjusted to OD_600_ 0.5 with LB medium; 45 µl OD_600_-adjusted culture and 45 µl LB or LB containing 20 µg ml^−1^ polymyxin B were mixed in a black, non-binding, PS 96-microtitre plate (F-bottom, Greiner). The plate was incubated at 37 °C in the plate reader (FluoStar Omega, BMG Labtech) with fluorescence monitoring before starting the experiment by addition of 10 µl 50 µg ml^−1^ resazurin in LB. The production of resorufin was followed at 37 °C with excitation and emission wavelengths of 540 and 580 nm, respectively. The results are derived from four biological replicates. Initial rates were determined by linear regression of the first 10 min after resazurin addition. Prism 10 (Graphpad) was used for regression analysis and plotting results.

### Alphafold predictions

Structural predictions of *Cst* CARF-TM were generated using Alphafold3 [31], with model weights provided by Google Deepmind. Structural predictions were made either in the presence or absence of ~50 1-palmitoyl-2-oleoyl-sn-glycero-3-phosphatidylglycerol (POPG) molecules and a cA_3_ ligand. Initial predictions were made for both a dimeric and trimeric conformation; however, pTM and ipTM scores for a trimeric conformation fell below the cutoff for a reliable prediction (0.45 and 0.32, respectively, compared to 0.8 and 0.77 for a dimer).

## Supporting information

S1 Fig HPLC analysis of mCpol reaction product. Aaf (**A**) or Sph (**B**) mCpol in vitro reactions in the presence of Mg^2+^ and ATP or NTP. Coinjection: HPLC samples of the 3′3′3′-cA_3_ standard and mCpol reaction were mixed and reinjected to check for matrix effects. The data underlying this Figure can be found in S1 Data.
(PDF)

S2 Fig Colorimetric pyrophosphate release assays for mCpol activity. Graphs for one representative experiment are shown. **A.** Standard curve. **B.** Progress curves for Sph mCpol. **C.** Progress curves for Cst mCpol. **D.** Standard curve for Aaf mCpol reaction. **E.** Progress curves for Aaf mCpol. Data for (D) and (E) only were blank (no mCpol, no pyrophosphate) corrected. The turnover was calculated based on the assumption that one cA_3_ molecule is produced per three pyrophosphates and that non-productive pyrophosphate release was negligible. The data underlying this Figure can be found in S1 Data.
(PDF)

S3 Fig Plasmid challenge using the type III-B CRISPR system. *E. coli* BL21(DE3) Star was transformed with Cst CARF-TM or the cA_3_-activated CRISPR-associated nuclease NucC. The type IIIB (Cmr) CRISPR effector from *Vibrio metoecus* (Vme), charged with non-targeting crRNA (Cmr[pUC]) or targeting (Cmr[Tet]) crRNA, was used to allow regulated cA_3_ production. A serial dilution of the transformation mixture was applied to selective plates. The cyclase component was under the control of the lactose-inducible T7 promoter, and the effector (CARF-TM or NucC) was under the control of the arabinose-inducible pBAD promoter. Upon induction of protein expression with 0.1% d-lactose and 0.2% l-arabinose, Vme Cmr deactivated Cst CARF-TM (marked by gold arrows).
(PDF)

S4 Fig Purification of Cst CARF-TM and CARF proteins. **A, B**: Chromatogram for size exclusion chromatography and SDS-PAGE analysis of selected fractions for Cst CARF-TM, respectively. **C, D**: Chromatogram for size exclusion chromatography and SDS-PAGE analysis of selected fractions for Cst CARF, respectively. Pooled fractions used for analysis are indicated by an orange box. The chromatography data underlying this Figure can be found in S1 Data.
(PDF)

S5 Fig N- or C-terminal His-tags abolish CARF-TM function in vivo. Expression of untagged CARF-TM in *E. coli* prevented colony growth, but this effect was abolished when an N- or C-terminal polyhistidine tag was present.
(PDF)

S6 Fig HPLC analysis of supernatant from heat-denatured CARF: Crn4 mixture after 30 min incubation at 37 °C. The ring nuclease Crn4 can degrade CARF-bound cA_3_. The molar ratio of CARF to Crn4 is indicated. The data underlying this Figure can be found in S1 Data.
(PDF)

S7 Fig Fluorescence microscopy of Cst CARF-TM in *E. coli* C43(DE3). Culture samples were taken 2 h after induction of protein expression and stained with the DNA stains Syto9 (membrane permeable) and propidium iodide (PI, membrane impermeable). The data underlying this Figure can be found in https://doi.org/10.17630/ac5e296c-fff0-4c63-b218-f13d08adb865.
(PDF)

S8 Fig Growth curves of Aaf CARF-TM and the full Aaf Panoptes system in *E. coli* MG1655. **A.** Upon induction of the Aaf CARF-TM effector under control of the arabinose promoter, a dose-dependent growth inhibition was observed (5 µM or 50 µM arabinose concentration) that was absent when the transmembrane domain was removed (CARF). **B.** When CARF-TM was expressed in the presence of Aaf mCpol, no growth defect was observed with 0.5 mM arabinose induction. Reflecting the situation in the Aaf genome, the *carf-tm* gene does not have its own ribosome binding site in this construct, which ensures translational coupling. ΔTM: mCpol-CARF. The mean and standard deviation from two biological replicates with three technical replicates each is shown. The data underlying this Figure can be found in S1 Data.
(PDF)

S9 Fig SDS-PAGE analysis of mCpols. M: PageRuler unstained (Thermo Scientific); 1: Cst mCpol wild type; 2: Cst mCpol[D63N,D64N]; 3: Sph mCpol wild type; 4: Aaf mCpol wild type.
(PDF)

S1 Data Data underlying Figs 2, 3E, 4, 5C, 5D, 5F, 5G, 6A, 6B, 6C, 7A–7E, S1A, S1B, S2A–S2E, S4A, S4C, S6, S8A, and S8B. (XLSX)

S2 Data Sequencing data for phage escapers in Fig 3F. (ZIP)

S1 Raw Images Uncropped gels, blots and plates including replicates. (PDF)

## Abbreviations

cA_3_: cyclic tri-adenylate
CARF: CRISPR-associated Rossmann Fold
CARF-TM: CRISPR-associated Rossmann fold-transmembrane
CBASS: cyclic nucleotide**-**based antiphage signalling system
cfu: colony-forming units
cOA: cyclic oligoadenylate
CV: column volumes
DIC: differential interference contrast
DLS: dynamic light scattering
EMSA: electrophoretic mobility shift assay
EOP: efficiency of plaquing
IM: inner membrane
mCpol: minimal CRISPR-related polymerase
NR: Nile Red
OM: outer membrane
OMPs: outer membrane proteins
PI: propidium iodide
Pycsar: Pyrimidine cyclase system for antiphage resistance
SEC: size exclusion chromatography
TEV: Tobacco Etch Virus
TM: transmembrane
