# Supplementary material for: Cyclic tri-adenylate controls a CARF-TM effector in type II Panoptes anti-phage systems
Source: PLoS Biol. 2026 Sep 15;24(9):e3003934. doi: 10.1371/journal.pbio.3003934 (PMC13577387; doi:10.1371/journal.pbio.3003934)
Supplement: Supplementary file 1 [file pbio.3003934.s001.pdf]

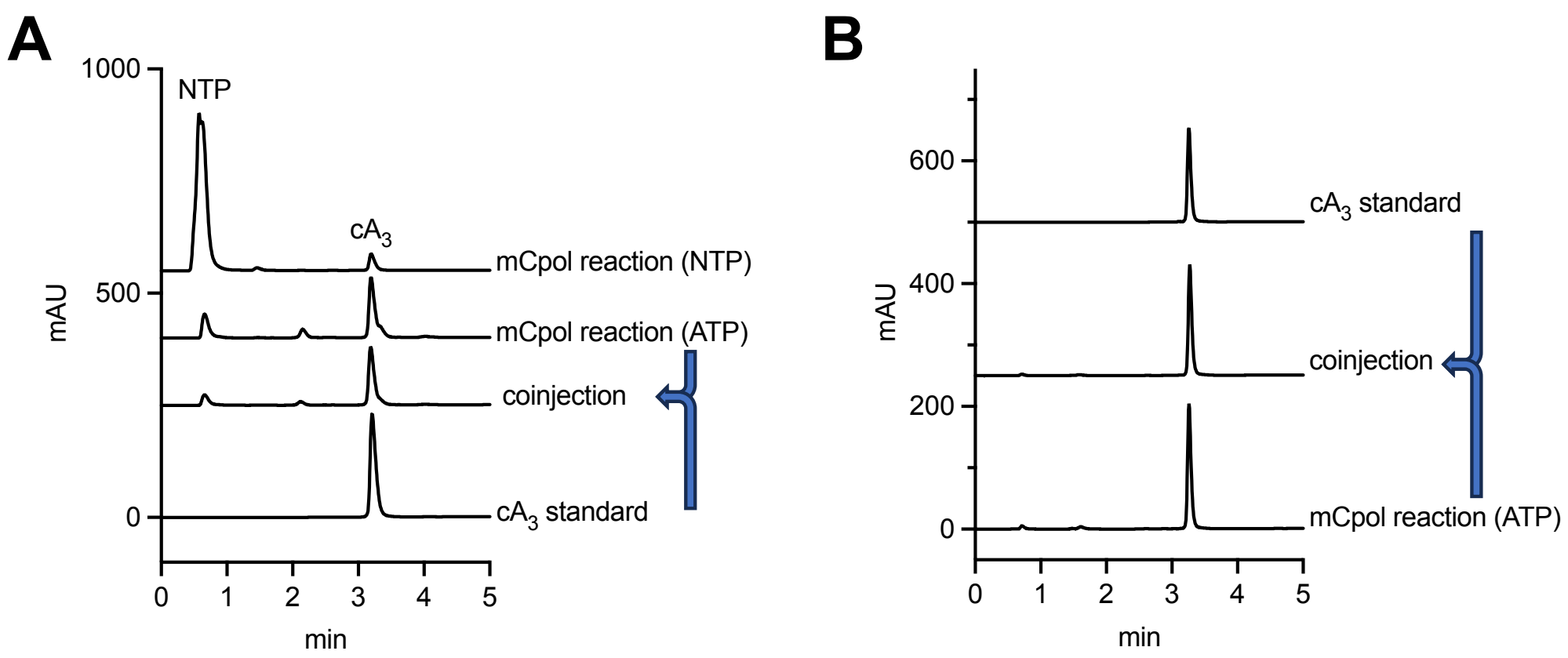

S1 Fig

HPLC analysis of mCpol reaction product. Aaf (**A**) or Sph (**B**) mCpol *in vitro* reactions in the presence of Mg^{2+} and ATP or NTP. Coinjection: HPLC samples of the 3'3'3'-cA_3 standard and mCpol reaction were mixed and reinjected to check for matrix effects.
