# Supplementary material for: Cyclic tri-adenylate controls a CARF-TM effector in type II Panoptes anti-phage systems
Source: PLoS Biol. 2026 Sep 15;24(9):e3003934. doi: 10.1371/journal.pbio.3003934 (PMC13577387; doi:10.1371/journal.pbio.3003934)
Supplement: Supplementary file 2 [file pbio.3003934.s002.pdf]

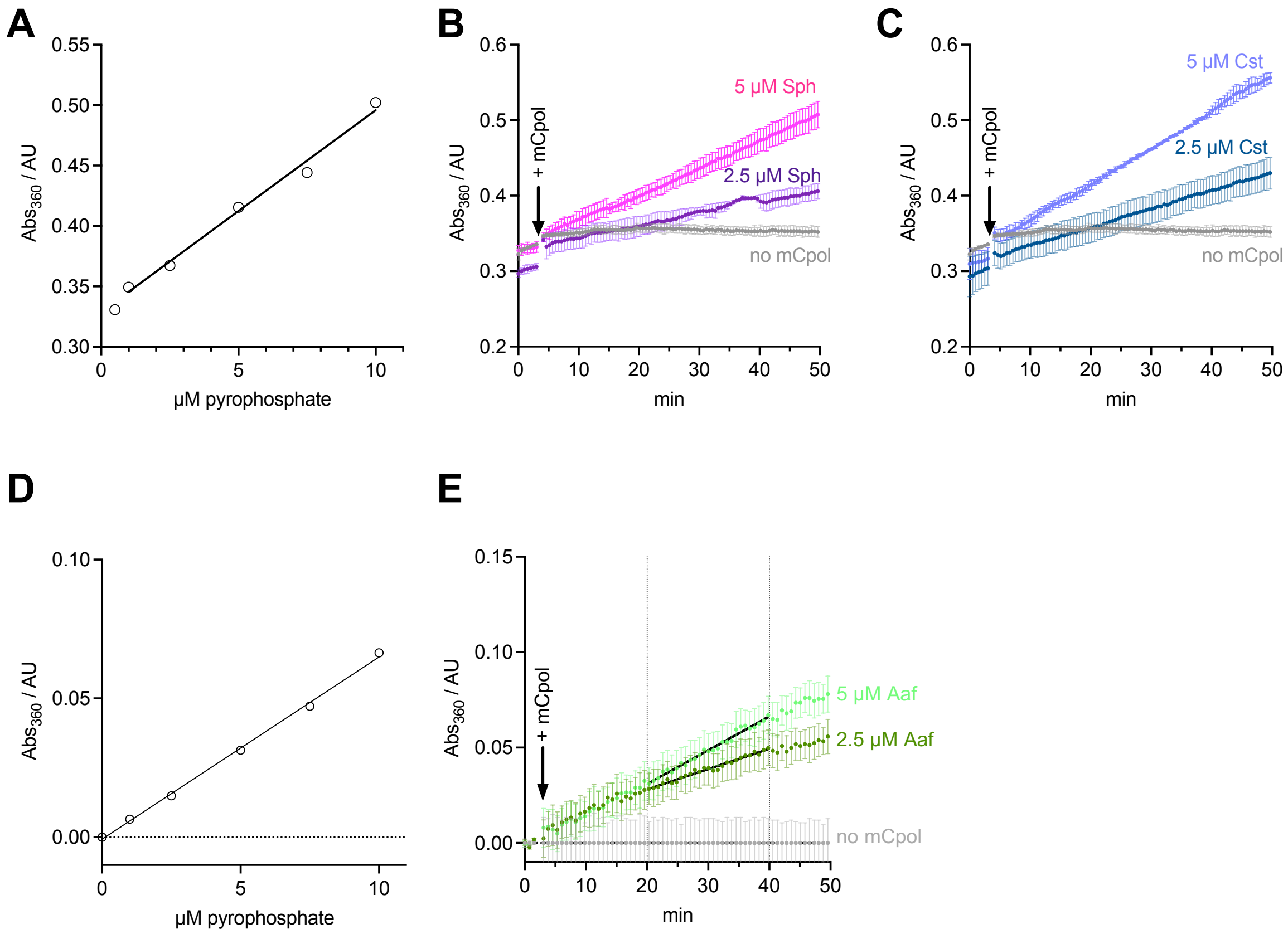

S2 Fig

Colorimetric pyrophosphate release assays for mCpol activity. Graphs for one representative experiment are shown. **A.** Standard curve. **B.** Progress curves for Sph mCpol. **C.** Progress curves for Cst mCpol. **D.** Standard curve for Aaf mCpol reaction. **E.** Progress curves for Aaf mCpol. Data for (**D**) and (**E**) only were blank (no mCpol, no pyrophosphate) corrected. The turnover was calculated based on the assumption that one cA_3 molecule is produced per three pyrophosphates and that non-productive pyrophosphate release was negligible.
