# Supplementary material for: Cyclic tri-adenylate controls a CARF-TM effector in type II Panoptes anti-phage systems
Source: PLoS Biol. 2026 Sep 15;24(9):e3003934. doi: 10.1371/journal.pbio.3003934 (PMC13577387; doi:10.1371/journal.pbio.3003934)
Supplement: Supplementary file 3 [file pbio.3003934.s003.pdf]

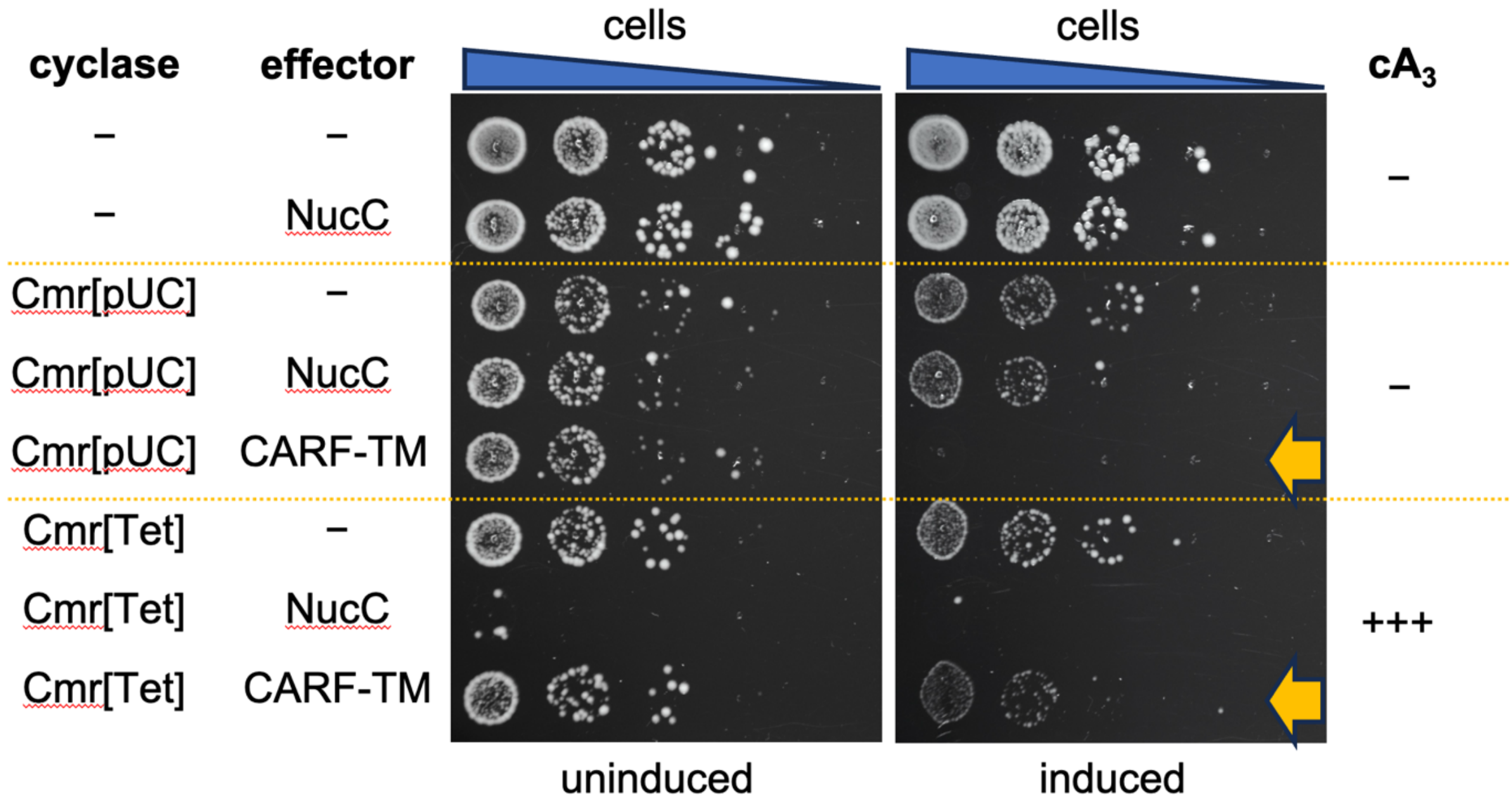

S3 Fig

Plasmid challenge using the type III-B CRISPR system. *E. coli* BL21(DE3) Star was transformed with Cst CARF-TM or the cA_3 activated CRISPR associated nuclease NucC. The type IIIB (Cmr) CRISPR effector from *Vibrio metoecus* (Vme), charged with non-targeting crRNA (Cmr[pUC]) or targeting (Cmr[Tet]) crRNA, was used to allow regulated cA_3 production. A serial dilution of the transformation mixture was applied to selective plates. The cyclase component was under control of the lactose-inducible T7 promoter, and the effector (CARF-TM or NucC) was under control of the arabinose-inducible pBAD promoter. Upon induction of protein expression with 0.1 % D-lactose and 0.2 % L-arabinose, Vme Cmr deactivated Cst CARF-TM (marked by gold arrows).
