# Supplementary material for: Cyclic tri-adenylate controls a CARF-TM effector in type II Panoptes anti-phage systems
Source: PLoS Biol. 2026 Sep 15;24(9):e3003934. doi: 10.1371/journal.pbio.3003934 (PMC13577387; doi:10.1371/journal.pbio.3003934)
Supplement: Supplementary file 4 [file pbio.3003934.s004.pdf]

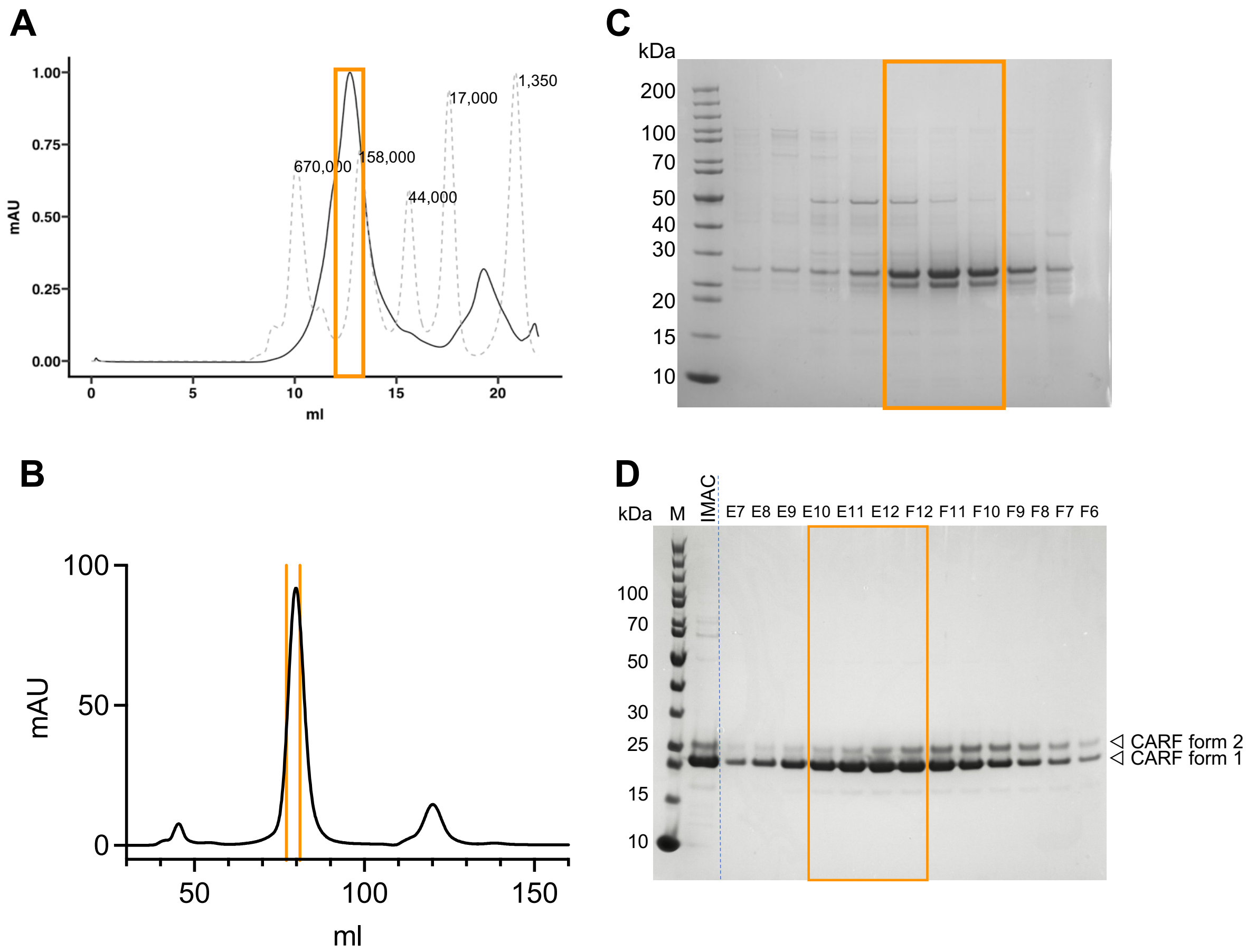

S4 Fig

Purification of Cst CARF-TM and CARF proteins. (A,B) Chromatogram for size exclusion chromatography and SDS-PAGE analysis of selected fractions for Cst CARF-TM. (C, D) Chromatogram for size exclusion chromatography and SDS-PAGE analysis of selected fractions for Cst CARF. Pooled fractions used for analysis are indicated by an orange box.
