# Supplementary material for: Cyclic tri-adenylate controls a CARF-TM effector in type II Panoptes anti-phage systems
Source: PLoS Biol. 2026 Sep 15;24(9):e3003934. doi: 10.1371/journal.pbio.3003934 (PMC13577387; doi:10.1371/journal.pbio.3003934)
Supplement: Supplementary file 5 [file pbio.3003934.s005.pdf]

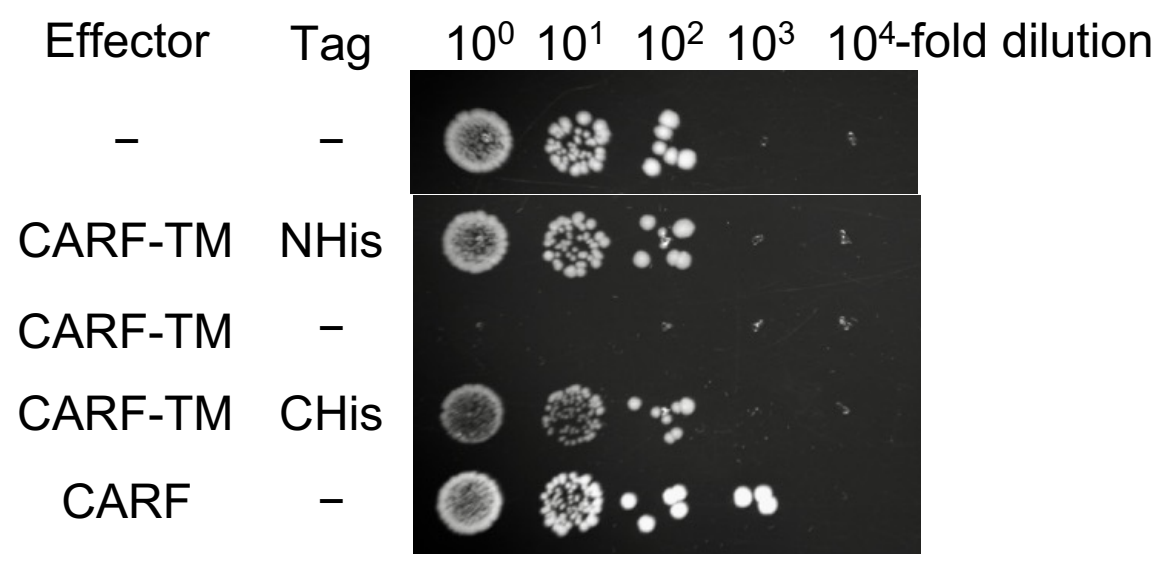

S5 Fig

N- or C-terminal His-tags abolish CARF-TM function *in vivo*. Expression of untagged CARF-TM in *E. coli* prevented colony growth, but this effect was abolished when an N- or C-terminal polyhistidine tag was present.
