# Supplementary material for: Cyclic tri-adenylate controls a CARF-TM effector in type II Panoptes anti-phage systems
Source: PLoS Biol. 2026 Sep 15;24(9):e3003934. doi: 10.1371/journal.pbio.3003934 (PMC13577387; doi:10.1371/journal.pbio.3003934)
Supplement: Supplementary file 6 [file pbio.3003934.s006.pdf]

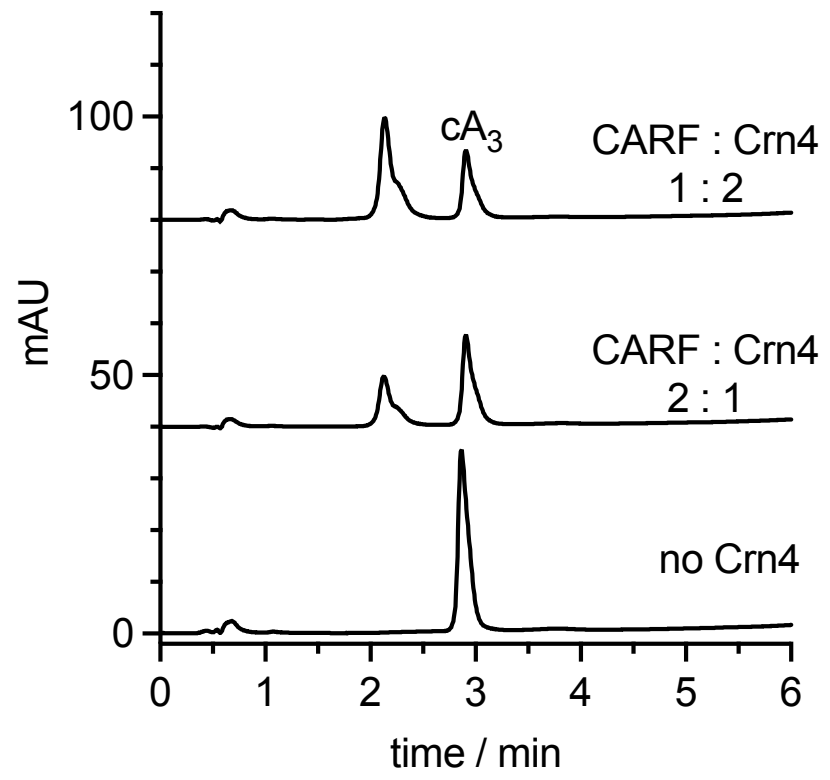


**S6 Fig**

**HPLC analysis of supernatant from heat denatured CARF : Crn4 mixture after 30 min incubation at 37 °C.** The ring nuclease Crn4 can degrade CARF-bound $cA_3$. The molar ratio of CARF to Crn4 is indicated.
