# Supplementary material for: Cyclic tri-adenylate controls a CARF-TM effector in type II Panoptes anti-phage systems
Source: PLoS Biol. 2026 Sep 15;24(9):e3003934. doi: 10.1371/journal.pbio.3003934 (PMC13577387; doi:10.1371/journal.pbio.3003934)
Supplement: Supplementary file 7 [file pbio.3003934.s007.pdf]

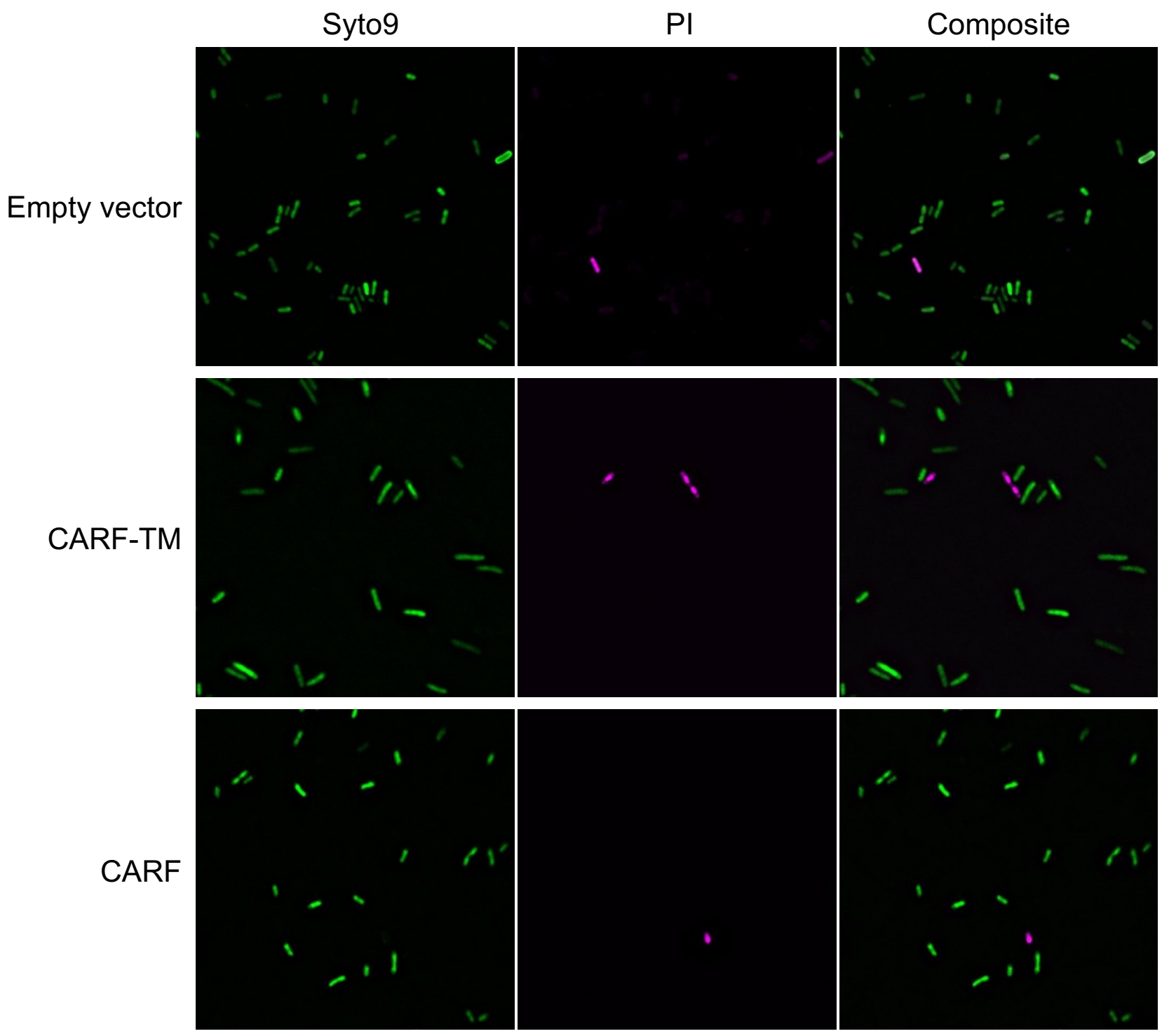

S7 Fig

Fluorescence microscopy of Cst CARF-TM in *E. coli* C43(DE3). Culture samples were taken 2 h after induction of protein expression and stained with the DNA stains Syto9 (membrane permeable) and propidium iodide (PI, membrane impermeable).
