# Supplementary material for: Cyclic tri-adenylate controls a CARF-TM effector in type II Panoptes anti-phage systems
Source: PLoS Biol. 2026 Sep 15;24(9):e3003934. doi: 10.1371/journal.pbio.3003934 (PMC13577387; doi:10.1371/journal.pbio.3003934)
Supplement: Supplementary file 8 [file pbio.3003934.s008.pdf]

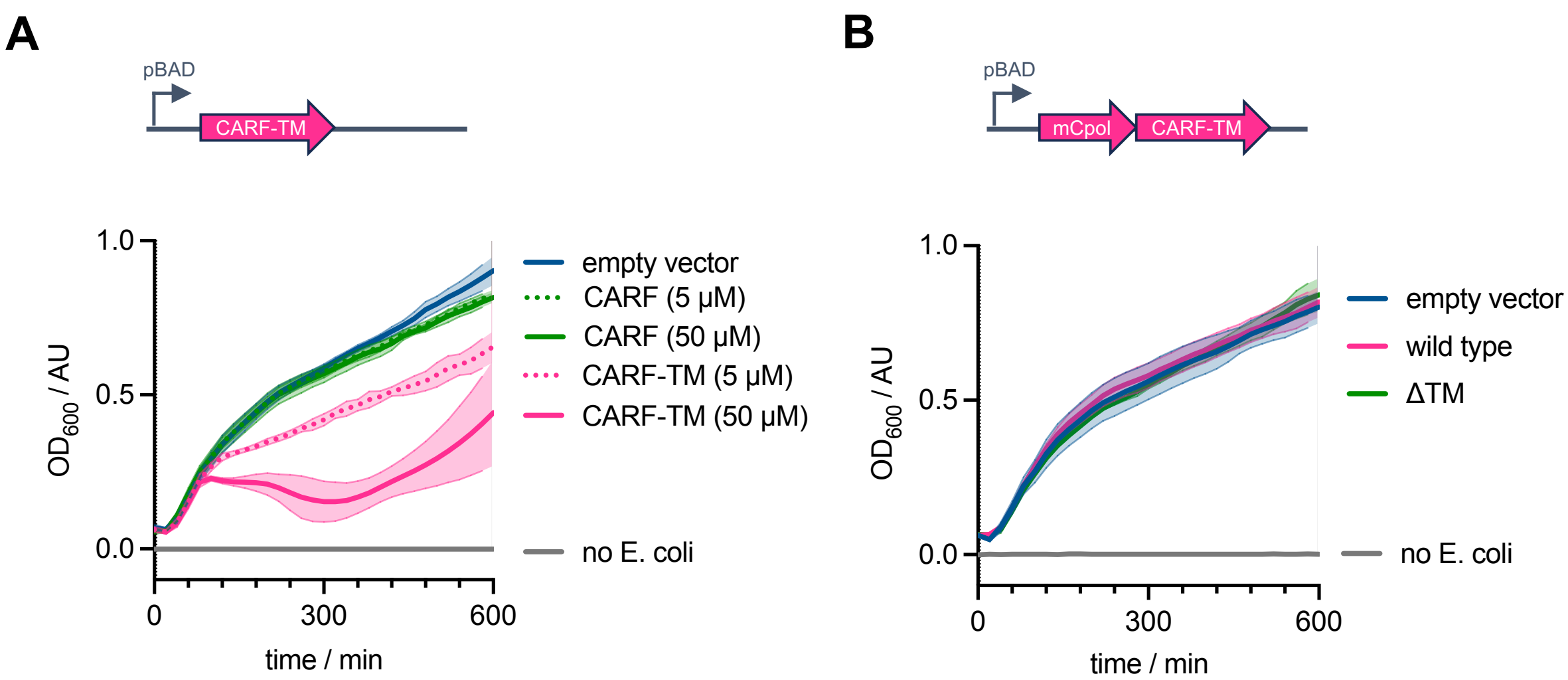

S8 Fig

Growth curves of Aaf CARF-TM and the full Aaf Panoptes system in *E. coli* MG1655. A. Upon induction of the Aaf CARF-TM effector under control of the arabinose promoter, a dose-dependent growth inhibition was observed (5 µM or 50 µM arabinose concentration) that was absent when the transmembrane domain was removed (CARF). **B.** When CARF-TM was expressed in the presence of Aaf mCpol, no growth defect was observed with 0.5 mM arabinose induction. Reflecting the situation in the Aaf genome, the *carf-tm* gene does not have its own ribosome binding site in this construct, which ensures translational coupling. ΔTM: mCpol-CARF. The mean and standard deviation from two biological replicates with three technical replicates each is shown.
