# Supplementary material for: Cyclic tri-adenylate controls a CARF-TM effector in type II Panoptes anti-phage systems
Source: PLoS Biol. 2026 Sep 15;24(9):e3003934. doi: 10.1371/journal.pbio.3003934 (PMC13577387; doi:10.1371/journal.pbio.3003934)
Supplement: Supplementary file 9 [file pbio.3003934.s009.pdf]

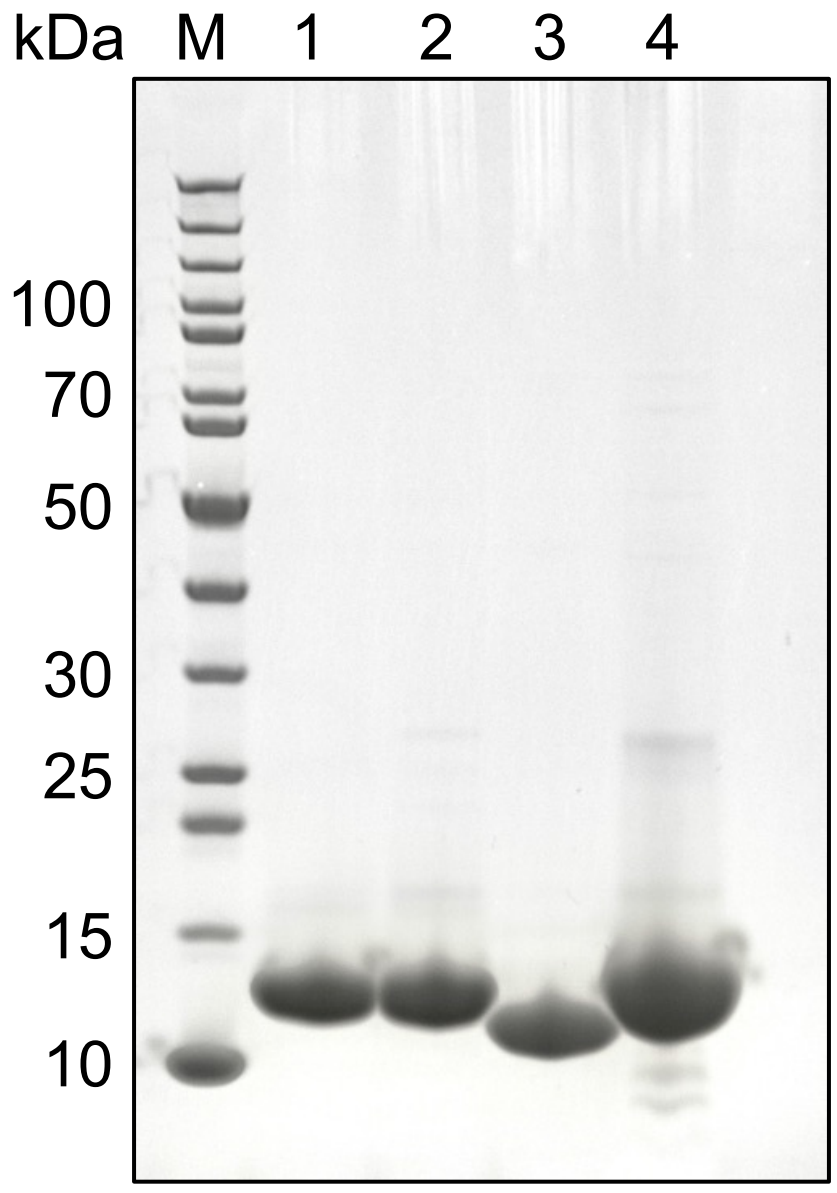

S9 Fig

SDS-PAGE analysis of mCpols. M: PageRuler unstained (Thermo Scientific); 1: Cst mCpol wild type; 2: Cst mCpol[D63N,D64N]; 3: Sph mCpol wild type; 4: Aaf mCpol wild type.
