# Supplementary material for: Cyclic tri-adenylate controls a CARF-TM effector in type II Panoptes anti-phage systems
Source: PLoS Biol. 2026 Sep 15;24(9):e3003934. doi: 10.1371/journal.pbio.3003934 (PMC13577387; doi:10.1371/journal.pbio.3003934)
Supplement: Supplementary file 10 [file pbio.3003934.s012.pdf]

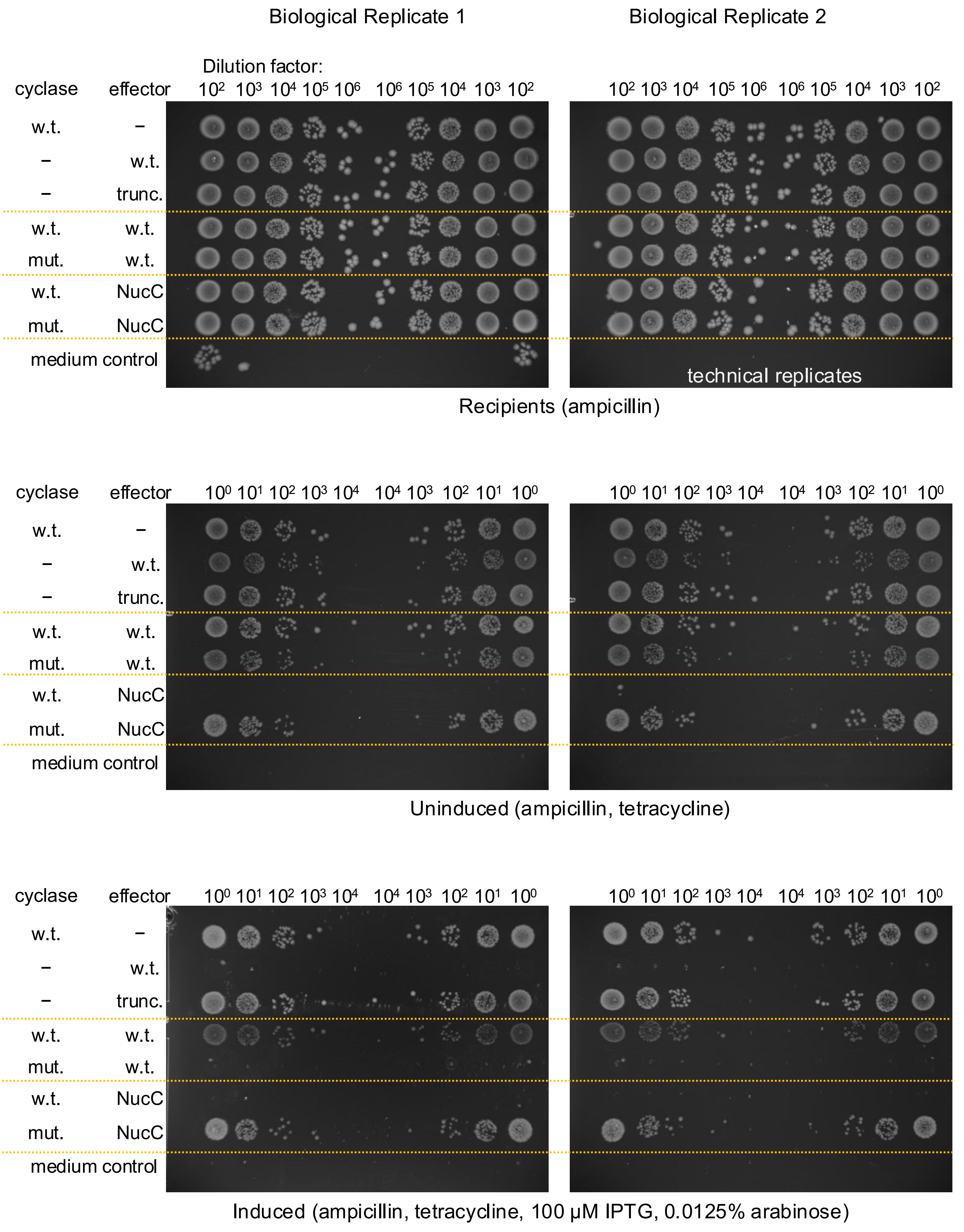

Figure 3A: Panoptes type II signals via cA_3 to deactivate the CARF-TM effector. Uncropped photos, control conditions and replicates of plasmid challenge assays. Cyclase w.t.: Cst mCpol wild type; cyclase mut.: Cst mCpol[D63N, D64N]; effector w.t.: Cst CARF-TM; effector trunc.: Cst CARF; effector NucC: Vme NucC; −: empty vector.

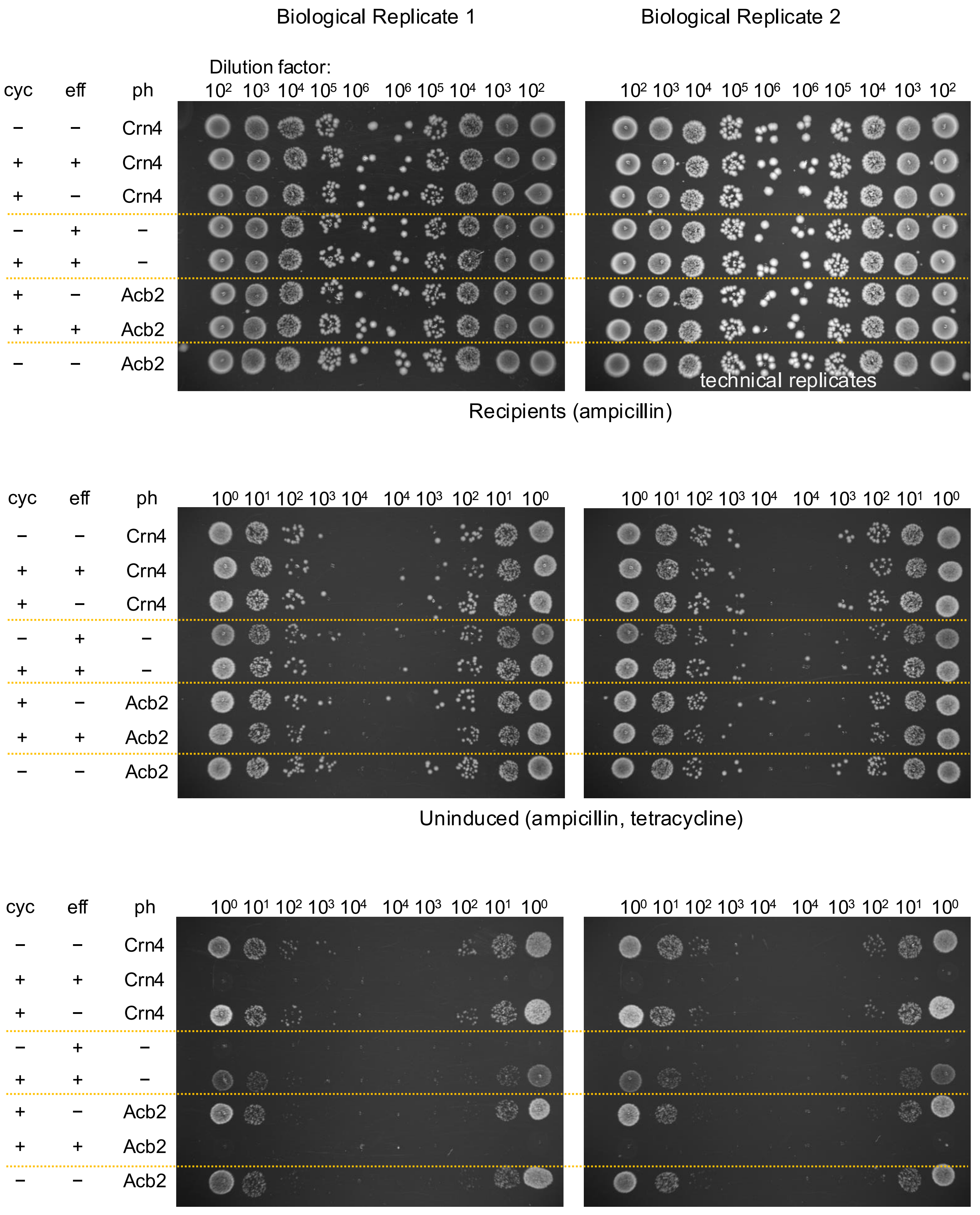

Figure 3B: Panoptes type II signals via cA_3 to deactivate the CARF-TM effector. Uncropped photos, control conditions and replicates of plasmid challenge assays. cyc: cyclase Cst mCpol; eff: effector Cst CARF-TM; ph: phage mimic; Crn4: Apr Crn4a ring nuclease; Acb2: Pae PaMx33 Acb2 cyclic oligonucleotide sponge; −: empty vector.

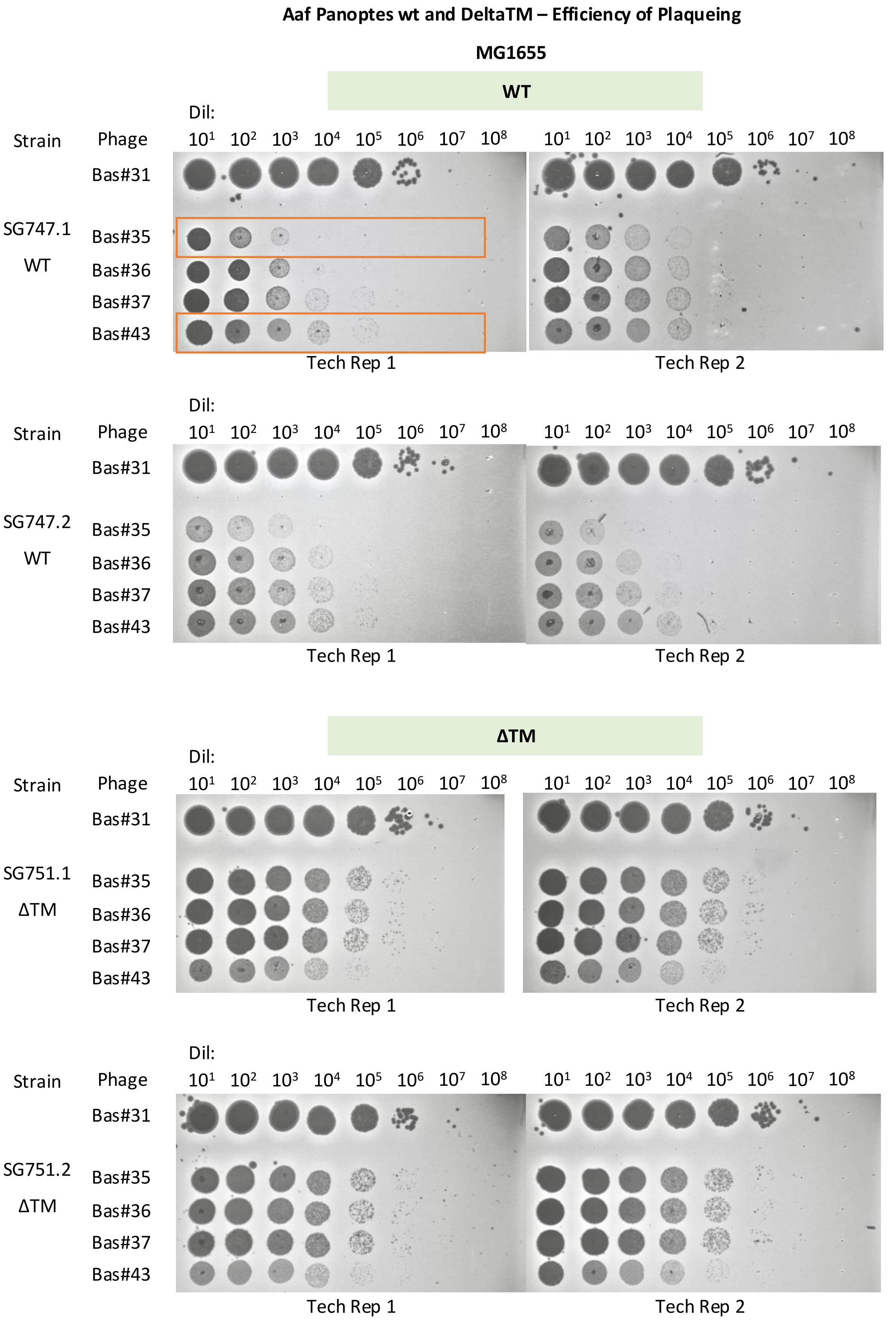

Figure 3C: Panoptes type II is able to reduce the plaquing efficiency of phages containing the ring nuclease Acb1.

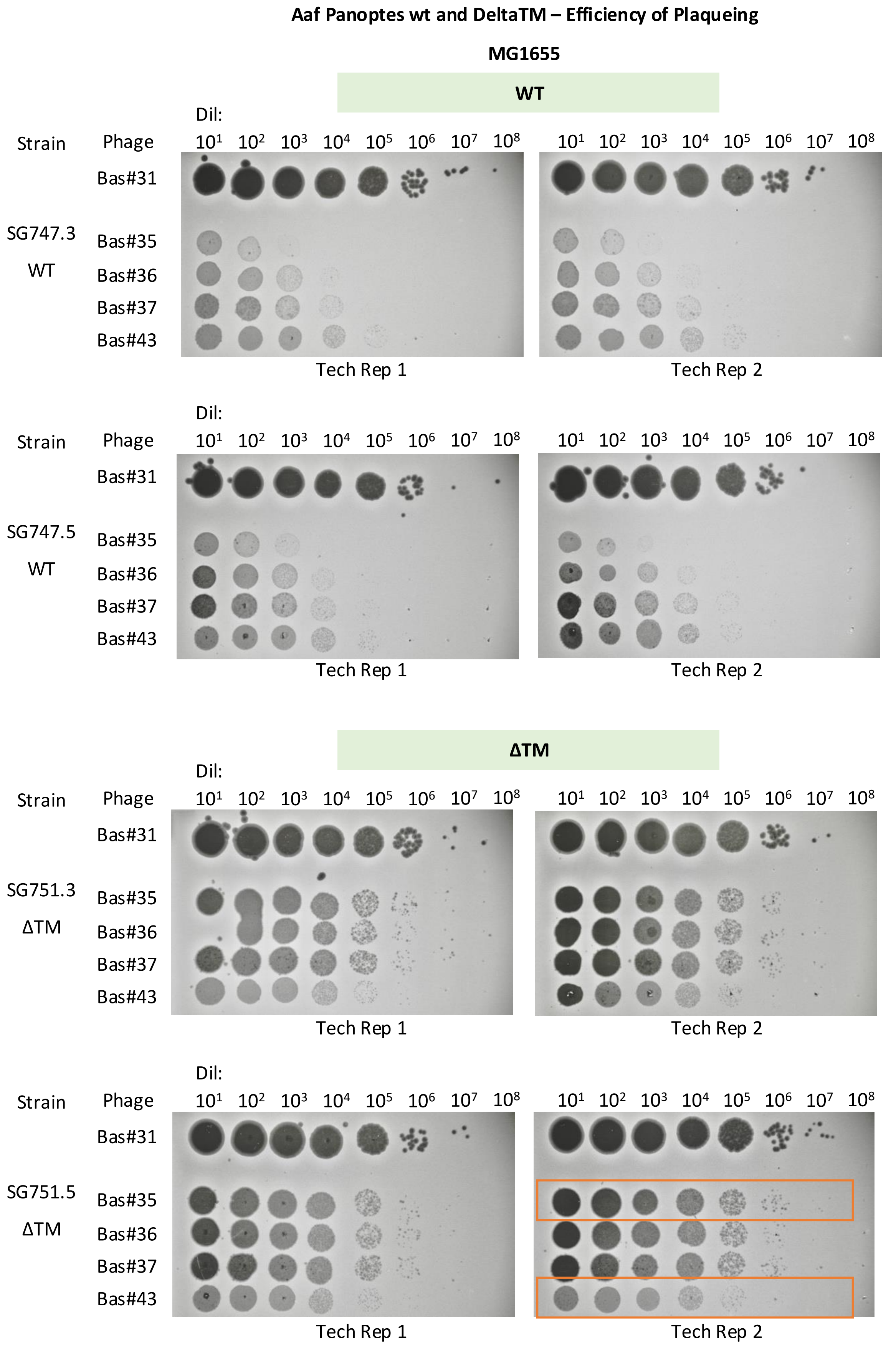

Figure 3C: Panoptes type II is able to reduce the plaquing efficiency of phages containing the ring nuclease Acb1.

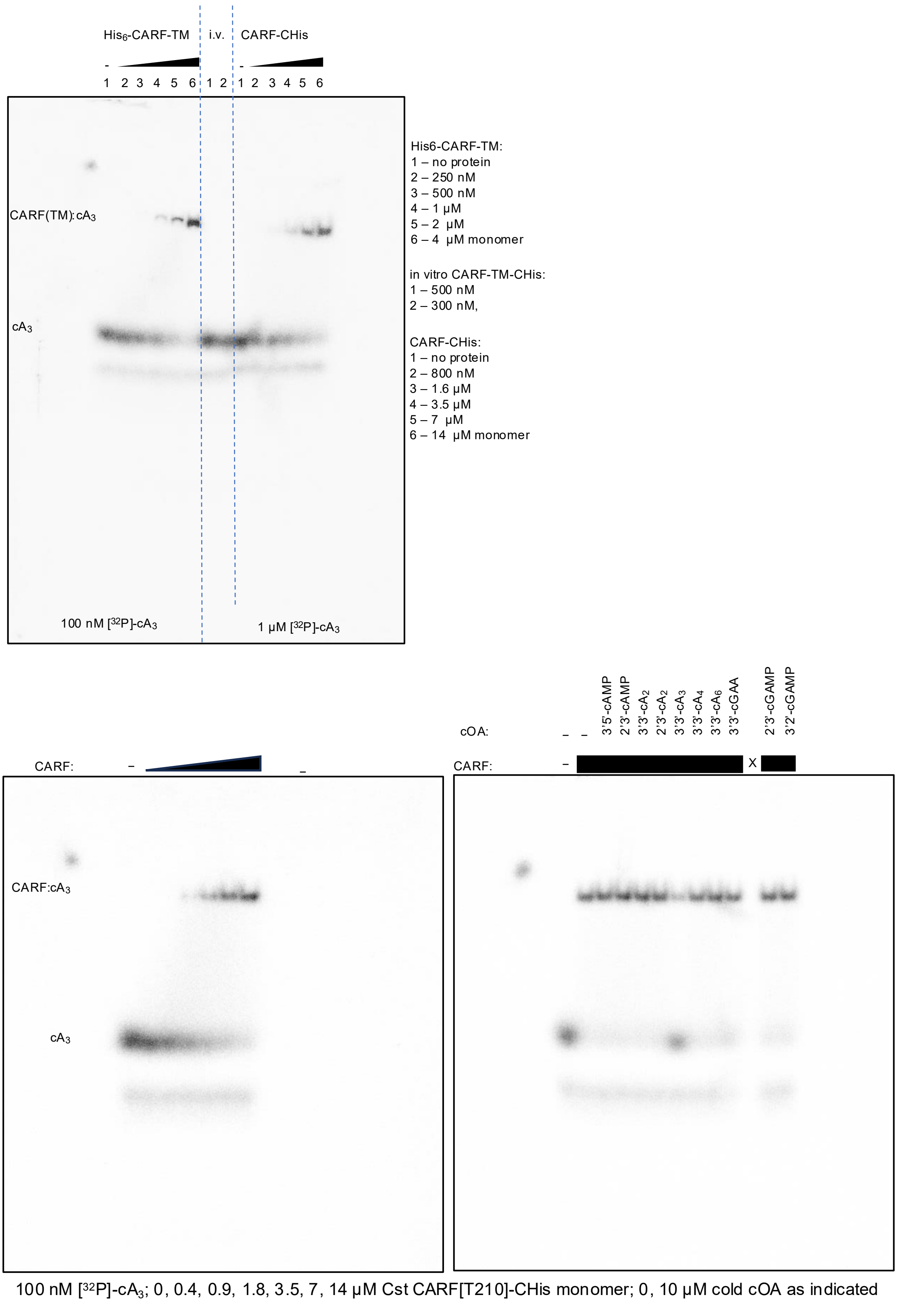

Figure 4 The full length and truncated CARF proteins bind cA_3 specifically. Electrophoretic mobility shift assay with $[^{32}P]$-cA_3 and full length Cst CARF-TM or truncated CARF. Ligand and protein concentrations are indicated in the Figure.

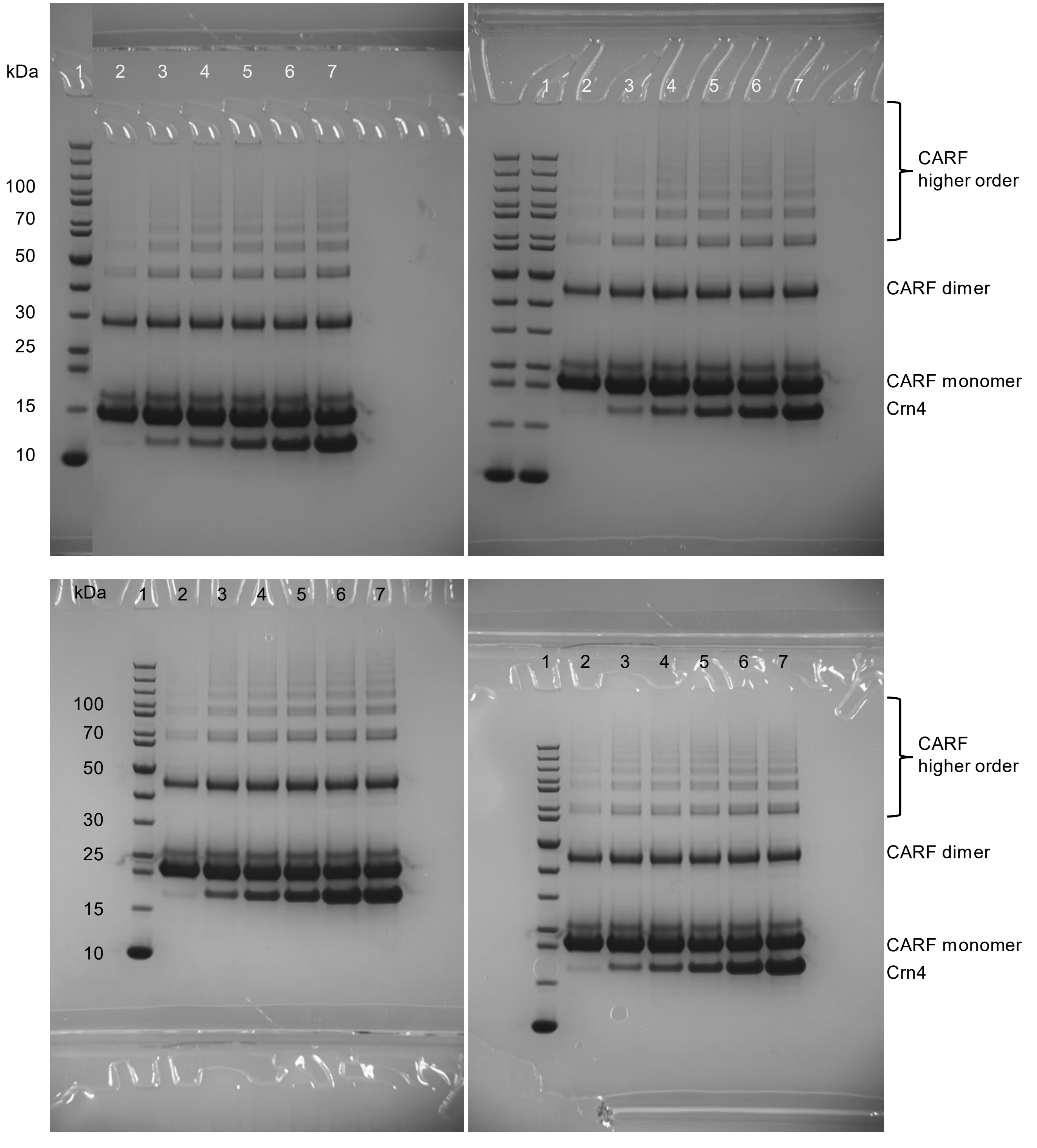

Figure 5E, F: Removal of cA_3 results in oligomerisation of CARF-TM. Cst CARF was chemically cross-linked following incubation with the ring nuclease Crn4. The reaction mixture was analysed on SDS-PAGE and relative abundance determined by densitometry using ImageJ. Lane 1: PageRuler unstained; lanes 2 – 7: 20 µM CARF dimer; lanes 3 – 7: 2.5, 5, 10, 20, 40 µM Crn4, respectively.

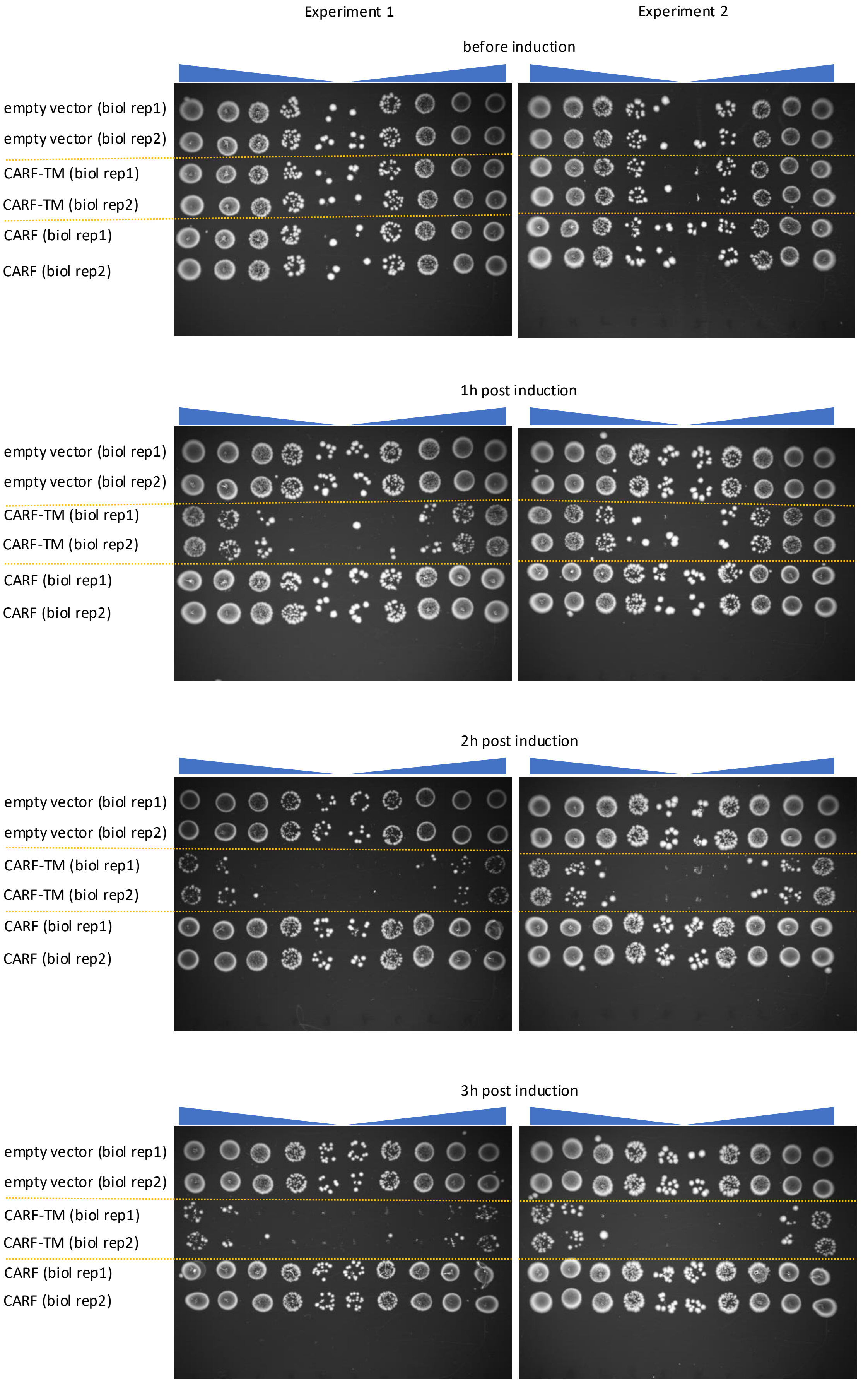

Figure 6C, D: The CARF-TM effector causes growth arrest in the absence of cA_3. Cells expressing CARF-TM lost their ability to proliferate as determined by microdilution assay. 2.5 µl diluted ($10^2 – 10^6$-fold) culture was applied per spot in technical duplicates (across) with 2 biological replicates per independent experiment.

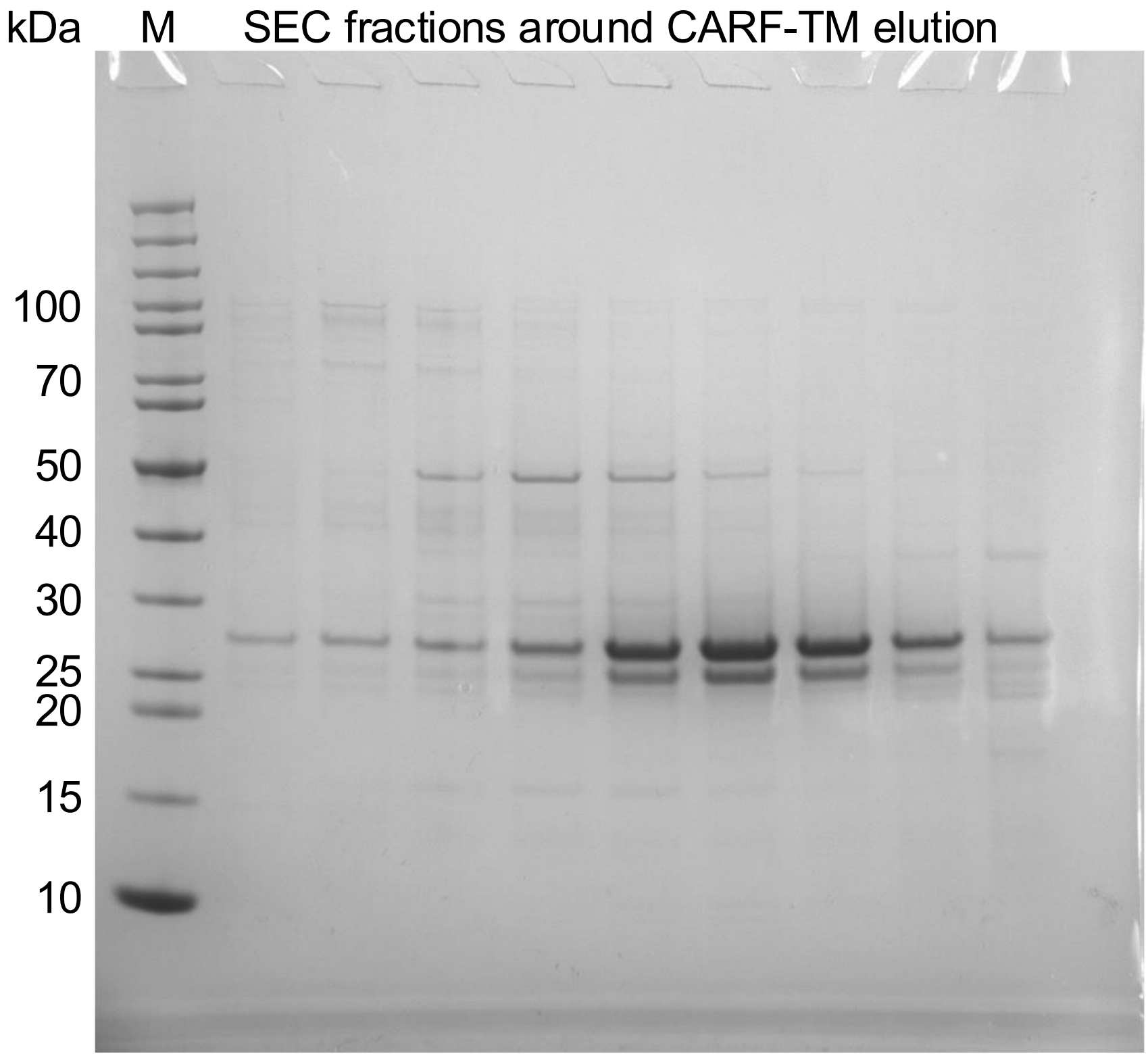

Fig. S4B. Purification of Cst CARF-TM protein. SDS-PAGE analysis of selected fractions from size exclusion chromatography of Cst CARF-TM. M: PageRuler Unstained (Thermo Scientific) protein marker.

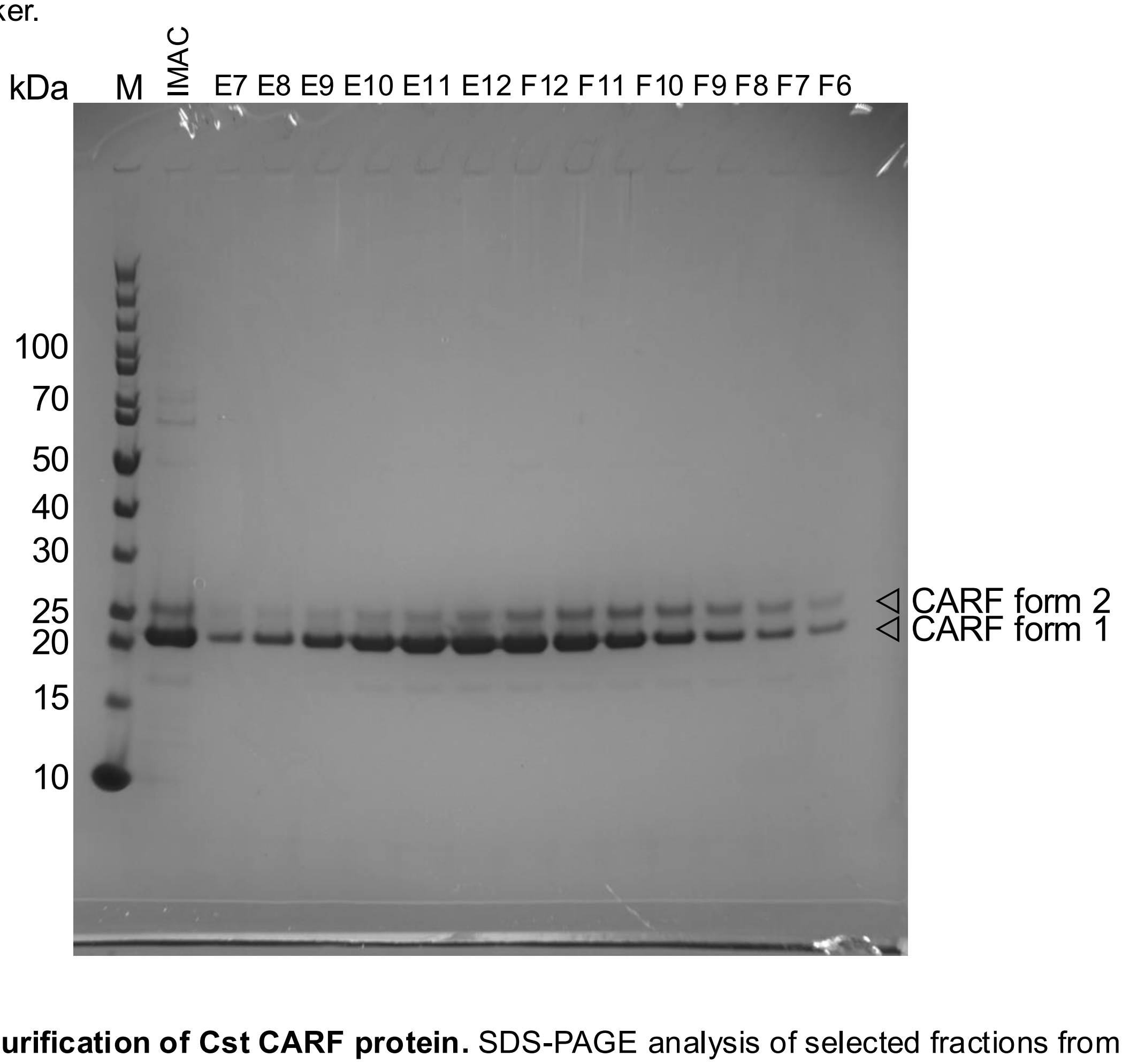

Fig. S4D. Purification of Cst CARF protein. SDS-PAGE analysis of selected fractions from size exclusion chromatography of Cst CARF. M: PageRuler Unstained (Thermo Scientific) protein marker. IMAC: pooled fractions from Ni^{2+}-affinity chromatography.

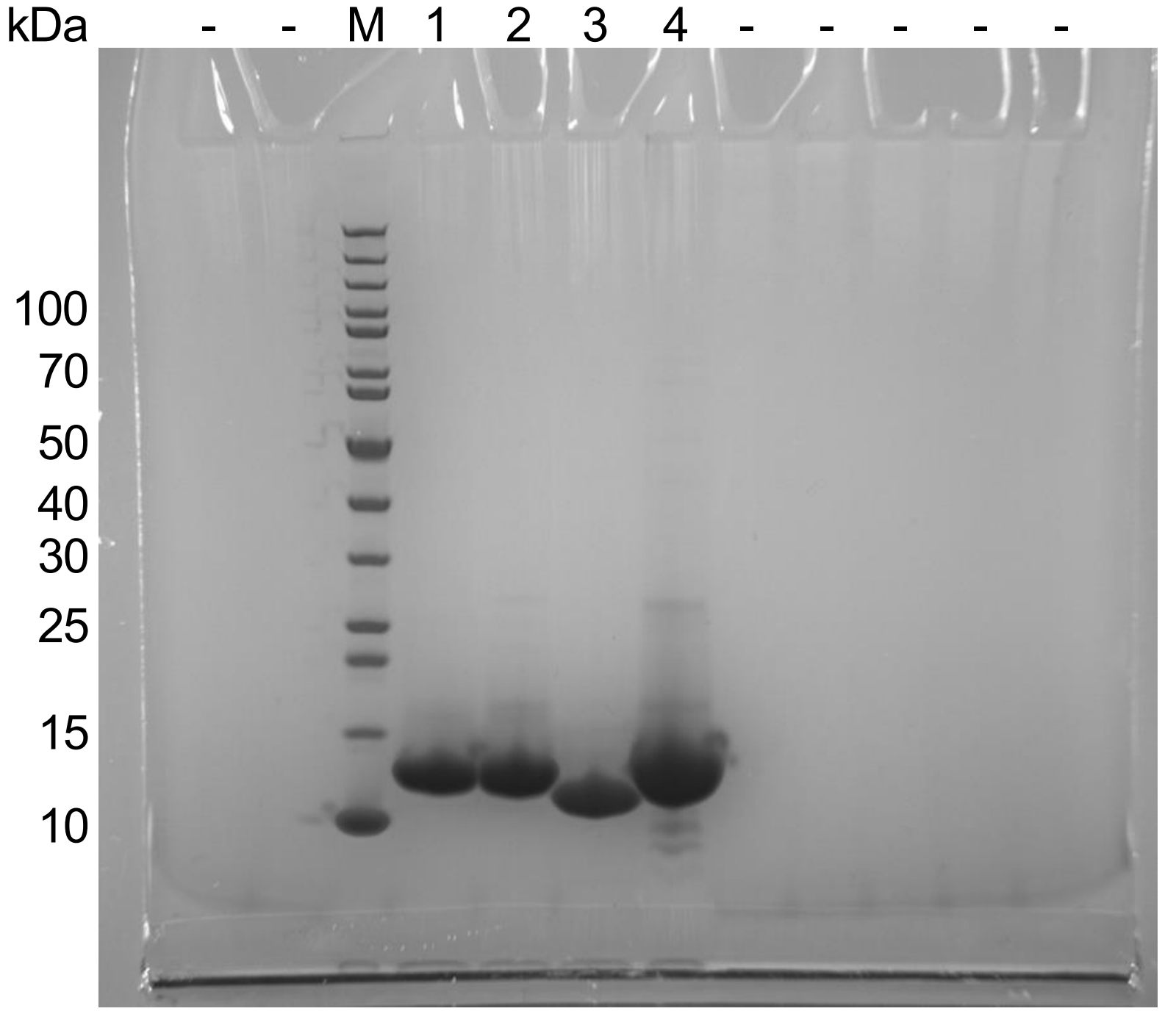

Fig. S9. SDS-PAGE analysis of mCpols. M: PageRuler unstained (Thermo Scientific); 1: Cst mCpol wild type; 2: Cst mCpol[D63N,D64N]; 3: Sph mCpol wild type; 4: Aaf mCpol wild type. -: empty well / nothing loaded.
