# Supplementary material for: Cyclic tri-adenylate controls a CARF-TM effector in type II Panoptes anti-phage systems
Source: PLoS Biol. 2026 Sep 15;24(9):e3003934. doi: 10.1371/journal.pbio.3003934 (PMC13577387; doi:10.1371/journal.pbio.3003934)
Supplement: Supplementary file 11 [file pbio.3003934.s015.pdf]


RESPONSE TO REVIEWERS' COMMENTS:

Thanks to all three reviewers for their expert and considered comments. We respond to all comments individually below. The headlines for the revision are:

1. We have added significant new data demonstrating that Panoptes type II can provide protection in vivo against phage encoding an Acb1 phosphodiesterase that degrades cA_3. A closely related phage with a disrupted *acb1* gene escapes the defence (figure 3C-E below). Furthermore, we have isolated escapers that carry mutations in their *acb1* gene (Figure 3F)

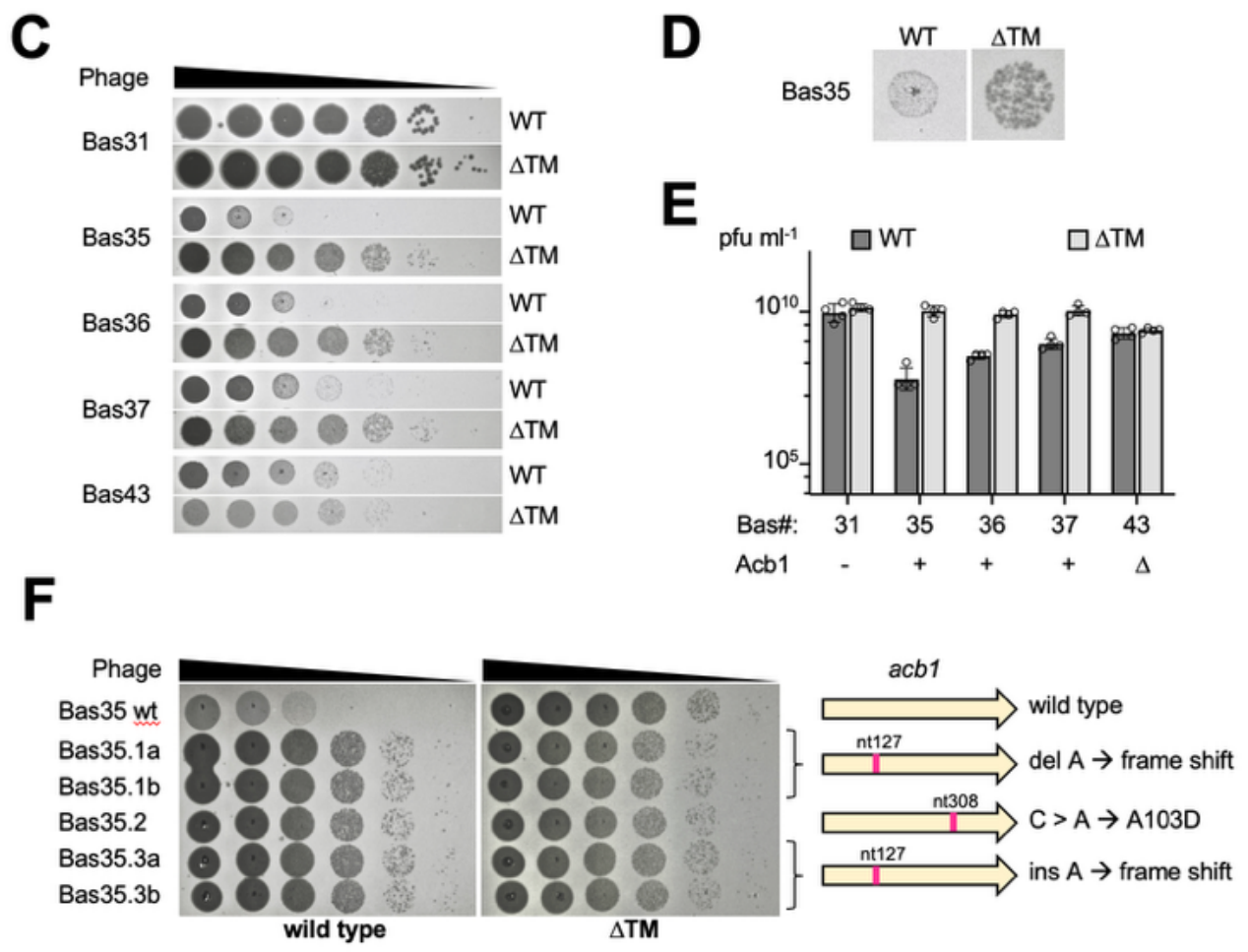

2. We provide new data on a third type II Panoptes system, from the Gram negative proteobacterial species *Ahniella affigens* (new data in Figs 2, 3, S2, S8, S9). This addition addresses several of the queries raised by reviewers.

Reviewer #1:

In this study, Grüshow et al. investigate the mechanism of a second type of Panoptes defence system, previously hypothesized by Sullivan et al. (2025). The system, referred to here as Panoptes type II, is a two-gene operon composed of a minimal CRISPR-related polymerase (mCpol) and a CRISPR-associated Rossmann fold transmembrane effector (CARF-TM). The authors propose that mCpol produces cyclic tri-adenylate (cA3), which maintains the CARF-TM effector in an inactive state. They show cA3 production by mCpol, cA3 binding to CARF-TM, and conformational changes and membrane-associated phenotypes when cA3 is depleted or sequestered using a phage sponge protein or a ring nuclease. Together, the work provides an interesting mechanistic framework for how this system may function. However, while the biochemical and mechanistic aspects are compelling, the study currently stops short of directly demonstrating anti-phage activity in a native infection context, which is important given the framing and title of the manuscript.
Overall, this is a carefully executed study demonstrating a very interesting mechanism. The biochemical data supporting cA3 production and its effect on the CARF-TM effector are convincing. However, given that the manuscript is framed around an anti-phage guard system, a more direct demonstration of in vivo relevance during phage infection would substantially strengthen the work.

Major comments

1. In vivo relevance in the native phage context. As such, the manuscript shows in vivo toxicity

of the system when cA3 is made unavailable for the CARF-TM effector and in vitro solid evidence of the cA3 production and effect on the effector. As the central claim is the proof of a second type of Panoptes, it would be of major interest to find a native infection context where the system is used to study it in vivo to link the in vivo and in vitro results presented here. Heterologous expression of said system into a strain and testing a large variety of phage could support more strongly the claim. Furthermore, the mutants designed into these studies could also be applied in an in vivo set up to correlate the results discussed here such as the cA3 synthesis.

Thank you for this suggestion, which was also made by referee3. In response, we have added significant new data (Figure 3C, D, E), demonstrating that Panoptes type II can provide defence against phage that encode the cA_3 degrading enzyme Acb1. Notably, one of the phages from the BASEL collection, Bas43, contains a nonsense mutation in the acb1 gene, rendering the phage insensitive to Panoptes type II. We also isolated escapers of phage Bas35 that render it insensitive to Panoptes defence. Sequencing reveals that the acb1 gene is mutated in these escapers (Fig. 3F) We hope that, together with the plasmid challenge experiments, this provide a firm foundation for the suggested function of Panoptes type II.

As we use the type II Panoptes from a third species, Ahniella affigens, for the phage assays, a number of changes were required to the manuscript. In Materials and Methods, cloning of the Aaf system is added and the experimental data for the phage assays is provided. We also included the characterisation of the Aaf mCpol that was performed analogous to Cst and Sph mCpols, and demonstrates that it also makes cA_3 with a slow rate. Figure 2 was updated to include the Aaf mCpol HPLC trace. Figure 3 has been extended to include phage assay results for Aaf panoptes.

2. The origin and framing of the Panoptes type II concept should be clarified
The manuscript cites previous work on Panoptes systems, but the distinction between the originally characterized mCpol/Cap15 systems and the mCpol/CARF-TM architecture studied here is not fully clear. Since this work builds on the hypothesis proposed by Sullivan et al. (2025), it would be helpful to explain this more explicitly in the introduction. In particular, the manuscript would benefit from a clearer presentation of how Panoptes type II was first identified, what genetic-context or co-occurrence analyses supported its prediction, and how its operon organization compares to previously described Panoptes subtypes. This would help readers better appreciate the novelty and rationale of the study.

Apologies that this issue lacked clarity. We have revised the introduction to make these issues clearer:
"*Although biochemical studies have focussed on Panoptes with a 2TMβ effector, about 40% of systems (hereafter Panoptes type II) are associated with an alternative CARF-family effector with a transmembrane domain (CARF-TM) [24]. Here, we demonstrate that the type II Panoptes mCpol generates a cyclic trinucleotide (cA_3) product which is bound by the associated CARF-TM effector. The system provides immunity against plasmids and phage expressing antidefence proteins that bind or degrade cA_3.*"

3. Cell permeability induced through CARF-TM apo from expression. The PI staining was presented as inconclusive after an induction of 1 or 2 hours but in the cell density and culturability assay they show a growth arrest at 2 hours. Maybe more time-points could be relevant in the PI staining then to observe clearer difference in cell permeability.

Thanks for this comment. We would/did not say that the PI staining was inconclusive – it seems quite conclusive in fact. The 1 – 2 h time period is relevant for biological activity, and we can see a very clear reduction in the ability of CARF-TM expressing cells to form colonies. The PI staining data demonstrates that these cells are not dead. We have amended the manuscript to clarify this:
"*This suggested that CARF-TM is not a fast-acting bactericidal agent when expressed in E. coli, but rather induces a growth arrest phenotype*."

The follow up experiments, including new data on the Aaf Panoptes system, expand on this point.

4. An additional control in the growth arrest experiments would be helpful. In the growth arrest experiments, only the CARF-TM protein was expressed in the cells and not the mCpol allowing for activity of CARF-TM. It would be interesting to have a control with the two proteins co-expressed to prove that mCpol save the growth arrest phenotype, even though it is already demonstrated in the transformation efficiency experiment.

Thanks for the suggestion. We have carried out a range of new experiments with the Panoptes system from *Ahniella affigens* (Aaf). We now present growth arrest experiments for the Aaf system in the presence and absence of the Aaf *mCpol* gene (Fig. S8). This demonstrates clearly that expression of mCpol prevents the toxicity observed with CARF-TM alone.

Minor comments
1. Figure quality should be improved:
a. Add legend including Mg+ in figure 2
done
b. Figure 3a: no condition with NucC alone as a control
NucC has been characterised in detail previously (REF 26,27) and on balance we feel that the phenotype of NucC in the presence of inactive mCpol is sufficient.
c. Figure 5: legend claims that only cA3 was able to displace the radiolabelled, but it was only the 3'3' and not the 2'3' cA3 isoform. No comment was made on why it is only one of the isoforms allowing this and not the other.
3'3'3' cA_3 is the only isoform generated by CRISPR systems and also by type II Panoptes. 2'3'3' cA_3 can be generated by some CBASS systems. We cannot rule out the possibility that the CARF-TM effector also binds 2'3'3' cA_3, but it is not obvious that this would be relevant to the biology. We have added a sentence and a reference to the results to clarify this:
"The alternative isomer 2'3'3'-cA_3, generated by some CBASS systems [32] was not tested."

d. Fig 6: In the title it is stated "in oligomerisation of CARF-TM" but the experiments were performed on the truncated version.

Apologies, this is now corrected.

Reviewer #2 (Naama Aviram):

Summary
This study characterizes the type II Panoptes anti-phage guard system, establishing cA3 as its

signaling molecule and demonstrating that the CARF-TM effector is held inactive as a dimer when cA3 is bound. Upon cA3 depletion, mimicking phage-encoded sponge or ring nuclease activity, the effector oligomerizes and causes outer membrane permeabilization and growth arrest. The biochemistry is clean and the overall logic of the guard mechanism is well supported. The work is a meaningful extension of the recently described type I Panoptes system and expands our understanding of how bacteria deploy decoy cyclic nucleotides as part of their immune repertoire. This is an important contribution to the field of bacterial anti-phage immunity, and I have several suggestions and concerns that I believe should be addressed to make the paper clearer and stronger.

Major concerns

1. Throughout the study, the heterologous E. coli expression system introduces an important confound that is never acknowledged. The slow mCpol turnover rate, the moderation of CARF-TM toxicity by co-expressed mCpol, and the apparent fitness cost of the system could all reflect suboptimal activity in a non-native host rather than intrinsic properties of the system. In native genomic context, production rates and toxicity may be quite different. This caveat should be explicitly discussed, particularly since the authors themselves note at the end of the Discussion that "further investigation will be required...ideally using a cognate host and genomic expression of the Panoptes system from native promoters". This acknowledgment should be moved forward and given more prominence.

Thanks for this important point. We have partly addressed these comments by adding data for a third Panoptes system, from Ahniella affigens, a proteobacterium somewhat closer to E. coli. The additional experiments (growth curves, new Figure S8) address the concerns about the moderation of CARFTM activity by mCpol, or rather the lack thereof when the two enzymes were expressed from two separate plasmids as in the plasmid challenge assay. This has been solved by expressing the Panoptes system as a single transcript as is shown in Figure S8. The problem was therefore not the heterologous host but the choice of expression plasmids. Tightly coupled co-expression of mCpol with CARF-TM does not seem to exhibit any fitness cost in terms of growth.
The in vitro and in vivo data for the Aaf system do correspond to our earlier analysis of the Cst system. Nevertheless, the point regarding heterologous systems is well made. We have added the following short paragraph to the end of the results section to reflect this:

"While these data all point to growth arrest as an outcome of type II Panoptes activation, a definitive conclusion will only be possible when the system is studied in its cognate host and native genomic context."

The turnover rate was measured in vitro with purified enzymes. Even though heterologous production of enzymes, their purification and use in a very artificial environment (ie outside a cell) can produce artefacts, it is a cornerstone of biochemical research.

We address this point along with related point 2 below.

2. The statement that slow mCpol turnover is "likely a common feature of CARF-TM-associated systems" is overstated given that only two enzymes were tested. The claim should be tempered accordingly. Additionally, the functional implications of a slow turnover rate cut both ways and deserve discussion: a low steady-state cA3 pool lowers the threshold for phage-mediated activation, but it also imposes a fitness cost that would likely be negatively selected over evolutionary time. These trade-offs should be addressed.

Thanks for these thoughtful comments. In response, we have characterised a third type II mCpol, from *Ahniella affigens*, a proteobacterium. The Aaf mCpol also synthesises cA_3 and has a low kinetic rate in vitro. We have modified and expanded the accompanying text as follows:

"The turnover number for all three mCpol enzymes was approximately 1 h^{-1} in vitro (Fig. S2), making mCpol significantly slower than Cas10 from Type III CRISPR systems [25]. Although in vitro reaction rates should be treated with caution, these data suggest mCpol may generate relatively low concentrations of cA_3 in cells. This could be consistent with a function as a decoy molecule for phage sponges and phosphodiesterases, providing sensitivity to changes in cA_3 concentration in vivo."

We have also expanded discussion of this aspect of the work in the discussion section:

"To function as a guard system, Panoptes must be sensitive to changes in the concentration of the cyclic nucleotide, in this case cA3, which keeps the effector inactive. The low turnover rate shared by three type II mCpols, at around 1 h-1 in vitro, suggests a low cellular concentration of cyclic nucleotide in vivo. Dissociation constants for cyclic nucleotide ligands by CARF family proteins are typically in the nanomolar range [43-45], and consistent with this we observe co-purification of cA3 with the CARF-TM protein. Nonetheless, bound cA3 exists in equilibrium with the free species, allowing competition with bulk cA3 in vitro and with sequestering/degrading proteins in vitro and in vivo. In such a scenario, rather few molecules of cA3 in the cell could be sufficient to keep the Panoptes effector in an inactive state, while providing a sensitive readout of phage interference."

3. In the section "The soluble CARF variant multimerises when cA3 is not bound": the statement that Crn4 "captures and partially degrades CARF-bound cA3" is not fully supported without rate measurements. If the rate of cA3 degradation by Crn4 is comparable to the intrinsic dissociation rate of cA3 from CARF, then Crn4 may simply be acting on the free released form rather than accessing the bound ligand. This distinction has mechanistic implications and should be discussed, or the claim should be softened.

We apologise for the imprecise language in this section. We envisage that cA_3 exists in equilibrium between CARF-TM bound and free states, consistent with the EMSA data. This would allow Crn4 to compete with CARF-TM for binding of free cA_3. We have clarified the wording of this section to make this clear:

"the ring nuclease can compete with the CARF-TM effector for cA_3 ligands in vitro (Fig. S6). This is consistent with the competition observed by free cA_3 in the EMSA experiments and with the in vivo data that demonstrate Panoptes activation by Crn4 (Fig. 3)."

4. Figure 7 is the most difficult to interpret in its current form and I think the conclusions drawn from it are overstated. Specific issues:

- The growth curves in B are presented on a log scale, which is unusual for this type of experiment - what does the data look like on a linear scale?

(NB: This is now figure 6.) A semi-log plot is not that unusual for growth curves (some would say it should be the standard format). It was chosen to allow a more direct comparison to plot 6C which is also in semi-log scale, as both measurements come from the same samples. Below is

a side-by-side comparison of the same plot as semi-log as in the manuscript and in linear y-scale. We don't propose to change this plot, but provide the raw data for readers.

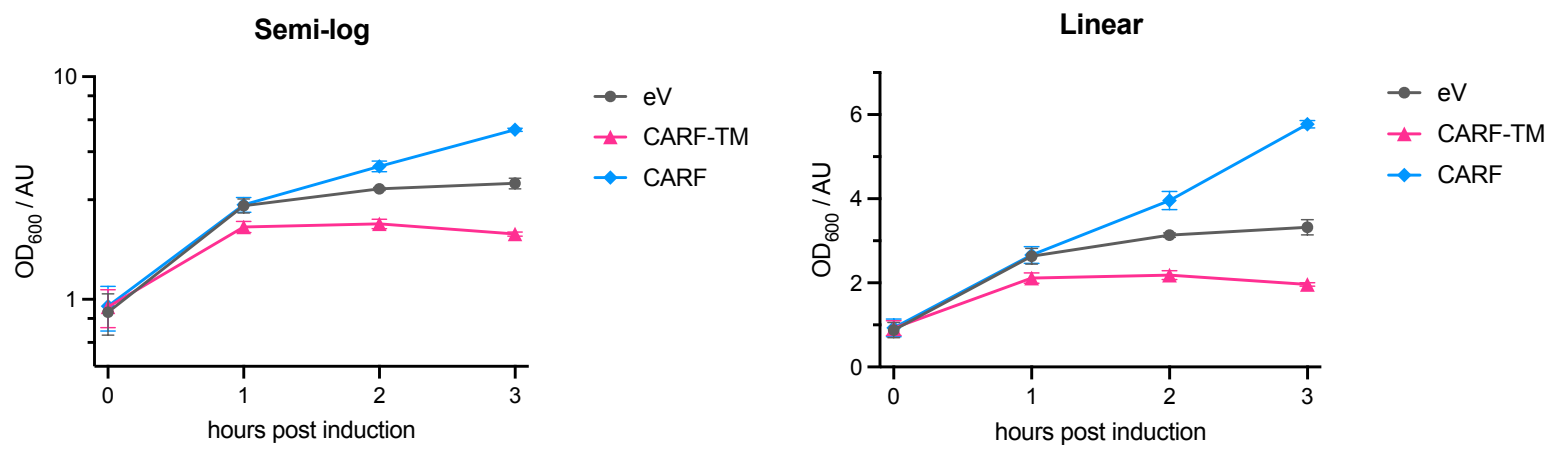

- Why does the empty vector condition plateau in panel B?

In our hands, most if not all (DE3) strains tend to plateau /reach stationary phase, at a relatively low cell density with IPTG induction. This is not a new observation and we provide some context and a relevant reference in the revision:

"The growth of cells with the empty vector control slowed significantly on IPTG addition – a phenomenon that was consistently observed and which may relate to the burden IPTG places on cells in a context-dependent manner [35]. We therefore investigated the equivalent growth curves using the Aaf Panoptes system under the control of an arabinose promoter, confirming that Aaf CARF-TM, but not truncated CARF, induced growth arrest in the absence of mCpol, with no growth defect in the presence of mCpol (Fig. S8)."

- Why does CARF plateau in panel C if OD is still increasing in B?

We don't have a definitive answer to this, but the overexpression of the CARF protein in the absence of cA_3 appears to result in large amounts of inclusion bodies in the cell and a change in cell morphology. Cell pellets have an altered appearance, appearing much "whiter" than normal cell pellets. It is possible that each cell therefore scatters light a little more effectively, leading to an increased optical density. The cfu count in figure 6C is therefore more informative.

- CARF-TM cells plateau in OD but drop in CFU, the authors should clarify why this is interpreted as evidence for a VBNC-like state and discuss it more explicitly rather than leaving it implicit. It could be helpful to explain why CFU counts drop.

Thanks for this comment. To clarify, we have removed reference to the VBNC state, which is not particularly helpful, and reworded the section as follows. Together with the new text at the end of the results highlighting the importance of not over-interpreting heterologous systems, we hope this acceptable.

"Microdilution and colony counting assays for the Cst effector demonstrated that whilst the strains with empty vector or expressing the CARF domain yielded a similar number of colony-forming units (cfu), those for the CARF-TM-expressing strain dropped by 2 – 3 orders of magnitude (Fig. 6C, D). Combined with the data from PI staining, this suggests cells experience growth arrest when the CARF-TM effector is activated. While the cells don't appear to lyse, as observed for type I Panoptes [31], they may still be functionally "dead" to all intents and purposes."

- The experiment appears to begin at a relatively late logarithmic stage, which compresses the dynamic range of the assay. Starting from a lower OD would give a clearer picture of the growth arrest phenotype.

We have added new data (Fig S8), for the Aaf Panoptes system, which start at a lower initial cell density. The overall picture is hopefully clear, particularly in combination with the colony counting data.

- Details of this experiment are also missing from the Materials and Methods.

Apologies for this oversight. We have added the details of the experiment in Materials and Methods. At this point we noticed that an older version of the resazurin experiment (not used in final manuscript) was described. This has now been corrected.

Minor concerns

1. Figure 1 is missing legends for panels C and D.

Apologies, an out-of-date version of figure 1 was included with the first version of the paper. This has now been corrected and these panels no longer exist.

2. In the section "Type II Panoptes is active in vivo and responds to cA3 levels in cells": NucC is introduced as a control without sufficient context for readers unfamiliar with its role in other defenses. A more detailed explanation of why it serves as a useful control here would help. The order in which conditions are described in the text should also be aligned with the order in which they appear in Figure 3.

Text has been altered to:
"We used Cst mCpol protein as the cyclase with either its cognate CARF-TM effector or NucC, a previously characterised dsDNA-endonuclease associated with CBASS and type III CRISPR defence [26, 27]. NucC is activated by cA_3 and was included as a control to detect cA_3 production."

3. On the same lines - The statement "as expected, expression of NucC alone resulted in efficient transformation" is confusing: there is no NucC-alone condition in the figure. Do the authors mean the catalytically inactive mCpol mutant? This should be clarified.

We apologise for the imprecise text here, which has been corrected to ("*expression of NucC with the catalytically inactive mCpol resulted in efficient transformation*").

4. The conclusion that cA3 binding is tight but not irreversible raises a question worth addressing in the Discussion: how would cA3 turnover occur in the cell? Is there a dedicated ring nuclease in the native host, or does the effector exchange cA3 through some other mechanism?

Thanks for this thoughtful comment. We have expanded the discussion to cover this:
"In order to function as a guard system, Panoptes must be sensitive to changes in the concentration of the cyclic nucleotide, in this case cA_3, which keeps the effector inactive. The low turnover rate shared by three divergent type II mCpols, at around 1 h^{-1} in vitro, suggests a low cellular concentration of cyclic nucleotide in vivo. Dissociation constants for cyclic nucleotide ligands by CARF family proteins are typically in the nanomolar range [44-46], and consistent with this we observe co-purification of cA_3 with the CARF-TM protein. Nonetheless, bound cA_3 exists in equilibrium with the free species, allowing competition with bulk cA_3 in vitro and with sequestering/degrading proteins in vitro and in vivo. In such a scenario, rather few molecules of

cA_3 in the cell could be sufficient to keep the Panoptes effector in an inactive state, while providing a sensitive readout of phage interference.
"

5. The Sypro Orange data (Figure 6C): the decrease in fluorescence upon Crn4 treatment is interpreted as burial of hydrophobic patches due to aggregation. Are there alternative structural explanations? For example, can the protein unfold and refold without aggregating? This should be briefly considered. More broadly, the connection between the fluorescence data in Figure 6C-D and the proposed structural changes would be strengthened by showing temporal resolution of the signal rather than endpoint measurements alone.

We agree there are other possible explanations. We have removed "due to protein aggregation" from the text. We are reluctant to speculate at this point if we observe aggregation / unfolding or a structural rearrangement as the experiments were conducted with the CARF domain. We included the data to demonstrate that structural changes do happen upon cA3 removal. Regarding the temporal resolution of the signal, we have experimented with a variety of ways to show these data in an accessible way and have come to the conclusion that showing the change in fluorescence at one time point is most effective. However, we have included an Excel spreadsheet with raw data for all data sets, including in vitro fluorescence data for readers whom would like to investigate the data in more detail.

6. Figure 7A requires representative microscopy images illustrating the PI staining phenotype, even if placed in supplementary material.

New Figure S7 now shows representative images for Syto9/PI staining

7. In the section "Expression of apo CARF-TM changes cell permeability in E. coli": the link between outer membrane permeabilization and the observed increase in metabolic rate should be made more explicitly. The logic connecting resazurin reduction kinetics to OM permeability is not immediately clear to the reader.

We have rewritten this section to clarify our logic here:

"By 1 h after induction, CARF-TM containing cells showed a faster rate of resorufin production compared to control cells (Fig. 7A). This could indicate an increase in NAD(P)H levels. However, we observed that the addition of the antibiotic polymyxin B, which permeabilises the outer membrane (OM) of Gram-negative cells [36, 37], yielded very similar results, though with a faster timescale (Fig. 7B). Indeed, there was no additive effect for CARF-TM expression and polymyxin B exposure. These data may indicate that the observed increase in resazurin reduction on both CARF-TM expression and polymyxin B treatment may be a consequence of increases in cell permeability, rather than a simple increase in intracellular NAD(P)H levels."

8. The Discussion would benefit from a deeper comparison of the type I and type II Panoptes phenotypes (e.g. inner membrane collapse and cell death versus outer membrane permeabilization and growth arrest) and from connecting the NucC-mediated response in classical CBASS/type III contexts to the new CARF-TM phenotype described here.

The Discussion has been expanded significantly to cover these and other points.

Reviewer #3:

The manuscript by Grüschow and colleagues describes a type II Panoptes anti-phage defence system in which mCpol synthesises cyclic tri-adenylate (cA3) and the associated CARF-TM effector is kept inactive in the presence of the ligand. The authors show that depletion or sequestration of cA3 activates the effector, causing an increase in membrane permeability and growth arrest. Overall, I found the study interesting, timely, and conceptually important for broadening our understanding of bacterial guard systems.
My main concern is that some of the mechanistic conclusions are stronger than the data currently support, as detailed in my specific comments below. Overall, the authors should make sure they better align the mechanistic claims with the current evidence and, when feasible, strengthen the physiological validation of the model proposed.

Major comments

1. A key conclusion is that cA3 keeps CARF-TM in an inactive dimeric state and that cA3 removal promotes oligomerisation and membrane-associated toxicity. However, the strongest structural and biochemical evidence for this transition comes from the truncated soluble CARF construct rather than the native full-length CARF-TM protein. Since the full-length protein was difficult to purify and tagged versions were non-functional in vivo, the manuscript currently relies in part on inference. I think the authors should either provide stronger data for the full-length effector or soften the wording throughout the manuscript so that it is clear which conclusions are shown directly and which are inferred from the soluble truncation.

This point is well made. We recognise the uncertainties here and have softened the language, including this key final paragraph for this section:

"Together, these observations suggested that cA_3 removal results in structural perturbations of the CARF dimer, leading to unfolding or aggregation in vitro. The absence of the TM domain in these studies is a significant caveat, however. Given the difficulty in studying the full length CARF-TM protein in vitro, we turned to in vivo analyses."

2. The study is built largely on heterologous expression in E. coli and plasmid challenge assays. These experiments are useful and consistent, but they do not directly demonstrate antiviral activity in a native or near-native context. The manuscript would be stronger with phage infection data, or at minimum with more explicit acknowledgment that the biological role of the system is inferred rather than directly demonstrated here.

Thanks for this suggestion, which was also made by referee 1. An EOP Phage assay and isolation of escape mutants has been included in Figure 3C-F, see response to Reviewer 1, point 1. We feel that this new data significantly strengthens the manuscript.

3. The data support the conclusion that apo CARF-TM affects cell physiology and membrane properties, but the claim of outer membrane permeabilization seems somewhat indirect. The Nile Red, resazurin, polymyxin B comparison, and plating assays are suggestive, but they do not fully distinguish outer membrane effects from broader envelope stress or indirect downstream consequences of inner membrane perturbation. I suggest moderating the language here unless additional direct membrane assays are included.

These comments are well made. We have moderated the language regarding permeabilization throughout the manuscript and expanded the final paragraph as follows:

"These data support a model where expression of apo CARF-TM in the inner membrane of E. coli results in permeabilization of the OM and growth arrest, but not rapid cell death. It should be noted however that effects observed on membrane permeabilization are indirect, with the significant caveat that the system is being studied in a heterologous host."

4. In figure 3, quantification of transformation efficiency across biological replicates would make the conclusions more rigorous and easier to assess.

We have now added significant new data on phage infection and defence to figure 3, including quantification of EOP data. Coupled with the qualitative plasmid challenge data, we feel this gives the reader a reasonable view of the in vivo phenotypes.

Minor comments

1. The manuscript should be careful not to overstate linkage assignment if the exact cA3 isomer was not established directly in this study.

We have now performed HPLC co-injection with commercially available 3'3'3'-cA_3 and do not observe peak splitting or broadening, consistent with 3'3'3' being the correct isomer (new Figure S1). Furthermore, 2'3'3'-cA_3 and 3'3'3'-cA_3 show different mobility on C18 (reversed phase) columns in HPLC as shown by Lowey et al (2020) Cell. Even though we do not use identical conditions, we see a clear separation of peaks for e.g. 2'3'- vs 3'3'-cA_2 indicating that our conditions are suited to separate regioisomers. To further shore up our assignment, we have included another piece of indirect evidence for 3'3'3'-cA_3 in Fig S3 (new). Cst mCpol is shown to activate Vme NucC which is a bona fide endonuclease that is activated by 3'3'3'-cA_3; and Cst CARF-TM is deactivated by a type III B CRISPR-Cas complex synthesising 3'3'3'-cA_3.

2. The Discussion is thoughtful, but several statements about the localisation and orientation of CARF-TM in the membrane seem more confident than the data justify.

In response to comments for all referees, we have expanded the discussion, pointed out areas of uncertainty and caveated the conclusions.

3. The data availability statement should be finalised before publication, as it currently indicates that the DOI is still pending.

We have now included most of the underlying data in Supplementary Data 1 and 2. Uncropped gel / blot images are also supplied. The only data covered by the institutional repository are the raw microscopy images. The DOI will be assigned when the paper is accepted, according to our library's common practise.
