# Supplementary material for: Cyclic tri-adenylate controls a CARF-TM effector in type II Panoptes anti-phage systems
Source: PLoS Biol. 2026 Sep 15;24(9):e3003934. doi: 10.1371/journal.pbio.3003934 (PMC13577387; doi:10.1371/journal.pbio.3003934)
Supplement: Supplementary file 12 [file pbio.3003934.s016.docx]

**The Editors**

**PloS Biology**

13 July 2026

Cyclic tri-adenylate controls a CARF-TM effector in type II Panoptes anti-phage systems

Dear Melissa, we resubmit our revised manuscript, which has dealt with all the editorial and technical requests for minor modifications, as detailed below.

One reference has been updated from Biorxiv to Nature Microbiology.

I’m afraid we don’t have a tracked changes version of the manuscript, just a clean one, as it was not saved before changes were accepted. I hope that, given the limited nature of the final edits, this is acceptable.

Malcolm White

**Specific requests:**

1) We routinely suggest changes to titles to ensure maximum accessibility for a broad, non-specialist readership, and to ensure they reflect the contents of the paper. In this case, we would suggest a some edits to the title, as follows. Please ensure you change both the manuscript file and the online submission system, as they need to match for final acceptance:

Changed in manuscript according to editor’s suggestion

2) Please add the weblink of the funding agencies in the Financial Disclosure statement in the manuscript details.

These have now been added.

3) Thank you for providing already the raw data from some figures. However, we are missing the numerical values for the following figures:
Figure 3E, 7E, S1AB, S4AB, S6, S8AB
NOTE: the numerical data provided should include all replicates AND the way in which the plotted mean and errors were derived (it should not present only the mean/average values).

*Additionally, it is not clear to which panel the numbers from Figure 5 and 6 belong to. Could you please specify?
done

Numerical values are now provided for:

Figures 2, 3E, 4, 5C, 5D, 5F, 5G, 6A – C, 7A – E, S1A, S1B, S2A – E, S4A, S4C, S6m S8A, S8B

4) Please cite the location of the data clearly in all relevant main and supplementary Figure legends, e.g. “The data underlying this Figure can be found in S1 Data” or “The data underlying this Figure can be found in …

This has been done, legends now include reference to excel S1 data and to Pure deposit for microscopy

5) Please ensure that you are using best practice for statistical reporting and data presentation.

Added statistical info to Figure 5F legend; only other place where statistical analysis was reported is Fig 6A, and statistical method is mentioned and p value indicated.

6) If you are reporting experiments where n ≤ 5, please plot each individual data point.

Changed Fig 6B and C, Fig 7A and B to show individual values, legend text has been amending accordingly

7) We require the original, uncropped and minimally adjusted images supporting all blot and gel results reported in the Figures S4CD, S9

Now provided as requested.

8) Please add a scale bar in all microscopy pictures

This has been added.

9) Please ensure that your Data Statement in the submission system accurately describes where your data can be found and is in final format, as it will be published as written there

Checked.

10) Per journal policy, if you have generated any custom code during the course of this investigation, please make it available without restrictions. Please ensure that the code is sufficiently well documented and reusable, and that your Data Statement in the Editorial Manager submission system accurately describes where your code can be found.

we don’t have any code

As you address these items, please take this last chance to review your reference list to ensure that it is complete and correct. If you have cited papers that have been retracted, please include the rationale for doing so in the manuscript text, or remove these references and replace them with relevant current references. Any changes to the reference list should be mentioned in the cover letter that accompanies your revised manuscript.

One reference changed to reflect final publication in Nature Micrbiology.

In addition to these revisions, you may need to complete some formatting changes, which you will receive in a follow up email. A member of our team will be in touch with a set of requests shortly. If you do not receive a separate email within a few days, please assume that checks have been completed, and no additional changes are required.

We expect to receive your revised manuscript within two weeks.

Journal Technical Requirements:

1) Please ensure that the funders and grant numbers match between the Financial Disclosure field and the Funding Information tab in your submission form. Note that the funders must be provided in the same order in both places as well. Currently, order of grants are different in both places.

This has been corrected.

2) Please refer to the following article for PLOS Biology's best-practice recommendations for data presentation found here: .

We report all p-values in the manuscript as they appear and have added statistical methods. We believe we are now fully compliant.
