# Supplementary material for: Cyclic tri-adenylate controls a CARF-TM effector in type II Panoptes anti-phage systems
Source: PLoS Biol. 2026 Sep 15;24(9):e3003934. doi: 10.1371/journal.pbio.3003934 (PMC13577387; doi:10.1371/journal.pbio.3003934)
Supplement: Supplementary file 13 [file pbio.3003934.s011.zip › Phage Sequencing/Acb1 Alignment.docx]

```
CLUSTAL O(1.2.4) multiple sequence alignment


YP_004009768.1      -MKFSEVDKGTYVSAKFTEDTLDRIEKIQKYLGLLNPVSRADLHTTICFSRVNVPFTASN	59
YP_010095090.1      MNNFKEFSTGLYVAAKFSEYTLDALENLQRSLKVPNPVPRDKLHTTIVYSRINVPYKVAS	60
7T26_A              --------SGLYVAAKFSESTLDALEELQRSLKLPNPVPRDKLHTTIVYSRVNVPYKVAS	52
YP_010089745.1      MLSFDEAAEGLYVAAKYSELTLDAIEMLQRELKVPNPVPREKIHSTICYSRVMVPYSVAS	60
QPB12449.1          MNKLDAYKTGLYVAAKYSNLTLDKIEKLQRDLRVPNPVPREKIHSTICYSRVNIPYVTSA	60
XNS48712.1          -MKFKNFTTGLYVAAKFSESTLDEIENLQRELKIPNPVPRHKIHSTICYSRVDVPYVCST	59
YP_007501173.1      MMKFQDFSSGLYVAAKFSDSTLDEIENLQRELKVPNPVPRHKIHSTICYSRVNVPYVVST	60
UVD32539.1          MINFQDLGSGLYVAAKFSDLTLDAIENLQRELKVPNPVPRHKIHSTVCYSRVNVPYTCSS	60
WP_016032569.1      -MTLNEVSEGLYVAAKFSELTLDALENLQRSLKVPNPVPREKFHSTICYSRVNIPYVVAS	59
YP_003734284.1      MMKTEDFAQGLYVAAKFSELTLDALENLQRTLKVPNPVPREKFHSTICYSRVNVPYTVAS	60
NP_861847.1         -MKFEDFSQGLYVAAKFSELTLDALEDLQRKLRIPNPVPREKLHSTICYSRVNIPYTTSS	59
QXV86094.1          MMEFKDFSTGLYVAAKFSELTLDALEELQRSLRVPNPVPREKIHSTICYSRVNVPYVPSS	60
QXV83283.1          MMEFKDFSTGLYVAAKFSELTLDALEELQRSLRVPNPVPREKIHSTICYSRVNVPYVPSS	60
QXV82233.1          MMEFKDFSTGLYVAAKFSELTLDALEELQRSLRVPNPVPREKIHSTICYSRVNVPYVPSS	60
NP_049750.1         MMEFKDFSTGLYVAAKFSELTLDALEELQRSLRVPNPVPREKIHSTICYSRVNVPYVPSS	60
bas35.3             MMEFKDFSTGLYVAAKFSELTLDALEELQRSLRVPNPVPREKIHSTICYSRVNVPYVPSS	60
QXV84986.1          -MEFKDFSTGLYVAAKFSELTLDALEELQRSLRVPNPVPREKIHSTICYSRVNVPYVPSS	59
QXV84985.1          ------------------------------------------------------------	0
                             * **:**::: *** :* :*: * : *** * .:*:*: :**: :*:  :

YP_004009768.1      ASLEVSNKNHLEVWQTNNGP-ALILCLESEYLVERHKYSRILGATYDYPDYKPHITLSYN	118
YP_010095090.1      GSFEVAKEGSLTIFETQDGNRALVLELESDYLSARHNYAKALGATYDFDDYRPHITLSYD	120
7T26_A              GSFEIADKGKLTVFETQSGNRALVLEMDSDYLSARHSYAKALGASYDYPDYRPHITLSYN	112
YP_010089745.1      GSFEVATSGHLEVWDHG-SP-VLVLVLDSDYLRCRHQYARAIGATHDYPDYTPHITLSYN	118
QPB12449.1          GSFEVSDGGKLEIWDTQDGK-TLVLVLDSEYLISRHNYARVLGATHDFDEYIPHITLSYN	119
XNS48712.1          GSFEVATSGELETWDTKDGK-VLVLKLDSDYLKFRHNYARALGATHDFPEYTPHITLSYN	118
YP_007501173.1      GSFEVANSGELQVWDTQDGR-TLVLVLDSDYLKFRHNYARALGATHDFDDYSPHITLSYN	119
UVD32539.1          GSFEVARKGHLEVWDHGESP-VLVLVLDSEYLKHRHRYARALGATHDFPDYTPHITLSYN	119
WP_016032569.1      GSFEVANSGHLEVWKTDDGS-TLVLVLDSDYLSCRHMYARAIGATHDFPDYTPHITLSYN	118
YP_003734284.1      GSFEVASTGHLEVWKHGDSP-VLVLVLNSEYLKCRHQYARTLGATHDFDDYTPHITLSYN	119
NP_861847.1         GSYSVAESGHLEVWKTDDGA-VLVLVLDSEYLRCRHQYARALGATHDFDDYTPHITLSYN	118
```

QXV86094.1	GSFEVASSGHLEVWKTQDGS-TLVLVLDSEYLRCRHMYARALGATHDFDDYTPHITLSYN	119

```
QXV83283.1          GSFEVASSGHLEVWKTQDGS-TLVLVLDSEYLRCRHMYARALGATHDFDDYTPHITLSYN	119
QXV82233.1          GSFEVASSGHLEVWKTQDGS-TLVLVLDSEYLRCRHMYARALGATHDFDDYTPHITLSYN	119
NP_049750.1         GSFEVASSGHLEVWKTQDGS-TLVLVLDSEYLRCRHMYARALGATHDFDDYTPHITLSYN	119
```

bas35.3	GSFEVASSGHLEVWKTQDGS-TLVLVLDSEYLRCRHMYARALGDTHDFDDYTPHITLSYN	119

QXV84986.1	------------------------------------------------------------	59

```
QXV84985.1          -------------------------MLDSEYLRCRHMYARALGATHDFDDYTPHITLSYN	35
                    .* .::  . *  :.   .  .*:* ::*:**  ** *:: :* ::*: :* *******:

YP_004009768.1      LGAQAVDLTIADVNMPIVVSHEVVEDLDLDWTKDKI	154
YP_010095090.1      VGPLQY--AG-KYAVPVILDREYSEELNLSWKDTLK	153
7T26_A              IGVLNF--SG-EYKVPVVLDREYSEELDLEWSDKD-	144
YP_010089745.1      VGVLKY--PKEKYPIRVVLDREYKEPLKLDWADDLK	152
QPB12449.1          VGELKF--SG-EYKLPIVLDYEYKEPLKLDWT----	148
XNS48712.1          IGPAHY--EG-EVKCHVVLDREYKEPLQLDWADDLK	151
YP_007501173.1      VGPAQF--SG-IVQVPVILDREYKEPLKINWTEDLK	152
UVD32539.1          VGPLSF--KG-EVQIPVVLDREYKEPLKLDWAEDLK	152
WP_016032569.1      VGPLTY--KG-EVQIPVVLDREYKEPLKLNWSADLK	151
YP_003734284.1      VGPLSF--SG-DVQIPVVLDREYKEPLKLDWAEDLK	152
NP_861847.1         VGQLSF--SG-DVAIPVILDREYKEPLKLDWAEDLK	151
QXV86094.1          VGPLSF--SG-DVQIPVVLDREYKEPLKLDWADDLK	152
QXV83283.1          VGPLSF--SG-DVQIPVVLDREYKEPLKLDWADDLK	152
QXV82233.1          VGPLSF--SG-DVQIPVVLDREYKEPLKLDWADDLK	152
NP_049750.1         VGPLSF--SG-DVQIPVVLDREYKEPLKLDWADDLK	152
bas35.3             VGPLSF--SG-DVQIPVVLDREYKEPLKLDWADDLK	152
QXV84986.1          ------------------------------------	59
QXV84985.1          VGPLSF--SG-DVQIPVVLDREYKEPLKLDWADDLK	68
                    :*              :::. *  * *.:.*
```
