# Supplementary material for: Cyclic tri-adenylate controls a CARF-TM effector in type II Panoptes anti-phage systems
Source: PLoS Biol. 2026 Sep 15;24(9):e3003934. doi: 10.1371/journal.pbio.3003934 (PMC13577387; doi:10.1371/journal.pbio.3003934)
Supplement: Peer review history

# Editor report: Decision Letter 0

**DOI:** 10.1371/journal.pbio.3003934.r001

**Authors:** Melissa Vazquez Hernandez

26 Feb 2026

Dear Dr White,

Thank you for submitting your manuscript entitled "Cyclic tri-adenylate signalling by a Panoptes anti-phage guard system with a CARF-TM effector" for consideration as a Research Article by PLOS Biology.

Your manuscript has now been evaluated by the PLOS Biology editorial staff as well as by an academic editor with relevant expertise and I am writing to let you know that we would like to send your submission out for external peer review.

However, before we can send your manuscript to reviewers, we need you to complete your submission by providing the metadata that is required for full assessment. To this end, please login to Editorial Manager where you will find the paper in the 'Submissions Needing Revisions' folder on your homepage. Please click 'Revise Submission' from the Action Links and complete all additional questions in the submission questionnaire.

Once your full submission is complete, your paper will undergo a series of checks in preparation for peer review. After your manuscript has passed the checks it will be sent out for review. To provide the metadata for your submission, please Login to Editorial Manager (https://www.editorialmanager.com/pbiology) within two working days, i.e. by Feb 28 2026 11:59PM.

If your manuscript has been previously peer-reviewed at another journal, PLOS Biology is willing to work with those reviews in order to avoid re-starting the process. Submission of the previous reviews is entirely optional and our ability to use them effectively will depend on the willingness of the previous journal to confirm the content of the reports and share the reviewer identities. Please note that we reserve the right to invite additional reviewers if we consider that additional/independent reviewers are needed, although we aim to avoid this as far as possible. In our experience, working with previous reviews does save time.

If you would like us to consider previous reviewer reports, please edit your cover letter to let us know and include the name of the journal where the work was previously considered and the manuscript ID it was given. In addition, please upload a response to the reviews as a 'Prior Peer Review' file type, which should include the reports in full and a point-by-point reply detailing how you have or plan to address the reviewers' concerns.

During the process of completing your manuscript submission, you will be invited to opt-in to posting your pre-review manuscript as a bioRxiv preprint. Visit http://journals.plos.org/plosbiology/s/preprints for full details. If you consent to posting your current manuscript as a preprint, please upload a single Preprint PDF.

Feel free to email us at plosbiology@plos.org if you have any queries relating to your submission.

Kind regards,

Melissa

Melissa Vazquez Hernandez, Ph.D.

Associate Editor

PLOS Biology

mvazquezhernandez@plos.org

# Peer review: Decision Letter 1

**DOI:** 10.1371/journal.pbio.3003934.r002

**Authors:** Melissa Vazquez Hernandez

15 Apr 2026

Dear Dr White,

Thank you for your patience while your manuscript "Cyclic tri-adenylate signalling by a Panoptes anti-phage guard system with a CARF-TM effector" was peer-reviewed at PLOS Biology. It has now been evaluated by the PLOS Biology editors, an Academic Editor with relevant expertise, and by several independent reviewers.

In light of the reviews, which you will find at the end of this email, we would like to invite you to revise your manuscript to thoroughly address the points raised. As you will see, all three reviewers find the study interesting and the biochemical and mechanistic framework compelling, but raise several concerns that need to be addressed before the work can be considered further. A central point raised by all reviewers is the lack of physiological validation of the proposed anti-phage function, particularly in a native or near-native infection context. In this regard, Reviewer 1 and Reviewer 3 suggest that demonstrating activity during phage infection, or in a more relevant host system, would substantially strengthen the manuscript. Reviewer 2 highlights important caveats associated with the heterologous E. coli system and requests that these limitations be more clearly acknowledged and discussed. All reviewers note that several mechanistic conclusions, particularly regarding CARF-TM oligomerisation, membrane permeabilization, and the role of cA3, are in part inferred from truncated constructs or indirect assays, and should therefore either be further supported experimentally or more carefully aligned with the data in the text. Additional requests include clarifying the conceptual framing and origin of “Panoptes type II,” improving the analysis and presentation of growth arrest and membrane phenotypes, and strengthening controls and quantification in key experiments.

IMPORTANT: after discussion with the Academic Editor, we recognize that addressing both physiological validation and additional mechanistic work would represent a substantial experimental effort. We therefore leave it to your discretion to decide how best to strengthen the manuscript, but we would expect that the revised version includes additional experimental work that addresses either (i) the in vivo/physiological relevance of the system, or (ii) a deeper and more direct mechanistic characterization, alongside a careful revision of the text to ensure that conclusions are fully supported by the data and that limitations are clearly acknowledged. We believe that addressing these points would significantly strengthen the manuscript and clarify its contribution. In line with this, if you would like to, we would be open to discuss a revision plan outlining how you intend to address these points, which we can discuss with the Academic Editor prior to revision, to avoid unnecessary or unfocused experimental effort.

Given the extent of revision needed, we cannot make a decision about publication until we have seen the revised manuscript and your response to the reviewers' comments. Your revised manuscript is likely to be sent for further evaluation by all or a subset of the reviewers.

In addition to these revisions, you will need to complete some formatting changes, which you will receive in a follow up email. A member of our team will be in touch with a set of requests shortly.

We expect to receive your revised manuscript within 3 months. Please email us (plosbiology@plos.org) if you have any questions or concerns, or would like to request an extension.

At this stage, your manuscript remains formally under active consideration at our journal; please notify us by email if you do not intend to submit a revision so that we may withdraw it.

**IMPORTANT - SUBMITTING YOUR REVISION**

Your revisions should address the specific points made by each reviewer. Please submit the following files along with your revised manuscript:

1. A 'Response to Reviewers' file - this should detail your responses to the editorial requests, present a point-by-point response to all of the reviewers' comments, and indicate the changes made to the manuscript.

*NOTE: In your point-by-point response to the reviewers, please provide the full context of each review. Do not selectively quote paragraphs or sentences to reply to. The entire set of reviewer comments should be present in full and each specific point should be responded to individually, point by point.

You should also cite any additional relevant literature that has been published since the original submission and mention any additional citations in your response.

2. In addition to a clean copy of the manuscript, please also upload a 'track-changes' version of your manuscript that specifies the edits made. This should be uploaded as a "Revised Article with Changes Highlighted" file type.

*Re-submission Checklist*

When you are ready to resubmit your revised manuscript, please refer to this re-submission checklist: https://plos.io/Biology_Checklist

To submit a revised version of your manuscript, please go to https://www.editorialmanager.com/pbiology/ and log in as an Author. Click the link labelled 'Submissions Needing Revision' where you will find your submission record.

Please make sure to read the following important policies and guidelines while preparing your revision:

*Published Peer Review*

Please note while forming your response, if your article is accepted, you may have the opportunity to make the peer review history publicly available. The record will include editor decision letters (with reviews) and your responses to reviewer comments. If eligible, we will contact you to opt in or out. Please see here for more details:

https://blogs.plos.org/plos/2019/05/plos-journals-now-open-for-published-peer-review/

*PLOS Data Policy*

Please note that as a condition of publication PLOS' data policy (http://journals.plos.org/plosbiology/s/data-availability) requires that you make available all data used to draw the conclusions arrived at in your manuscript. If you have not already done so, you must include any data used in your manuscript either in appropriate repositories, within the body of the manuscript, or as supporting information (N.B. this includes any numerical values that were used to generate graphs, histograms etc.). For an example see here: http://www.plosbiology.org/article/info%3Adoi%2F10.1371%2Fjournal.pbio.1001908#s5

*Blot and Gel Data Policy*

We require the original, uncropped and minimally adjusted images supporting all blot and gel results reported in an article's figures or Supporting Information files. We will require these files before a manuscript can be accepted so please prepare them now, if you have not already uploaded them. Please carefully read our guidelines for how to prepare and upload this data: https://journals.plos.org/plosbiology/s/figures#loc-blot-and-gel-reporting-requirements

*Protocols deposition*

To enhance the reproducibility of your results, we recommend that if applicable you deposit your laboratory protocols in protocols.io, where a protocol can be assigned its own identifier (DOI) such that it can be cited independently in the future. Additionally, PLOS ONE offers an option for publishing peer-reviewed Lab Protocol articles, which describe protocols hosted on protocols.io. Read more information on sharing protocols at https://plos.org/protocols?utm_medium=editorial-email&utm_source=authorletters&utm_campaign=protocols

Thank you again for your submission to our journal. We hope that our editorial process has been constructive thus far, and we welcome your feedback at any time. Please don't hesitate to contact us if you have any questions or comments.

Sincerely,

Melissa

Melissa Vazquez Hernandez, Ph.D.

Associate Editor

PLOS Biology

mvazquezhernandez@plos.org

------------------------------------

REVIEWERS' COMMENTS:

------------------------------------

Reviewer #1:

In this study, Grüshow et al. investigate the mechanism of a second type of Panoptes defence system, previously hypothesized by Sullivan et al. (2025). The system, referred to here as Panoptes type II, is a two-gene operon composed of a minimal CRISPR-related polymerase (mCpol) and a CRISPR-associated Rossmann fold transmembrane effector (CARF-TM). The authors propose that mCpol produces cyclic tri-adenylate (cA3), which maintains the CARF-TM effector in an inactive state. They show cA3 production by mCpol, cA3 binding to CARF-TM, and conformational changes and membrane-associated phenotypes when cA3 is depleted or sequestered using a phage sponge protein or a ring nuclease. Together, the work provides an interesting mechanistic framework for how this system may function. However, while the biochemical and mechanistic aspects are compelling, the study currently stops short of directly demonstrating anti-phage activity in a native infection context, which is important given the framing and title of the manuscript.

Overall, this is a carefully executed study demonstrating a very interesting mechanism. The biochemical data supporting cA3 production and its effect on the CARF-TM effector are convincing. However, given that the manuscript is framed around an anti-phage guard system, a more direct demonstration of in vivo relevance during phage infection would substantially strengthen the work.

Major comments

1.In vivo relevance in the native phage context. As such, the manuscript shows in vivo toxicity of the system when cA3 is made unavailable for the CARF-TM effector and in vitro solid evidence of the cA3 production and effect on the effector. As the central claim is the proof of a second type of Panoptes, it would be of major interest to find a native infection context where the system is used to study it in vivo to link the in vivo and in vitro results presented here. Heterologous expression of said system into a strain and testing a large variety of phage could support more strongly the claim. Furthermore, the mutants designed into these studies could also be applied in an in vivo set up to correlate the results discussed here such as the cA3 synthesis.

2.The origin and framing of the Panoptes type II concept should be clarified

The manuscript cites previous work on Panoptes systems, but the distinction between the originally characterized mCpol/Cap15 systems and the mCpol/CARF-TM architecture studied here is not fully clear. Since this work builds on the hypothesis proposed by Sullivan et al. (2025), it would be helpful to explain this more explicitly in the introduction. In particular, the manuscript would benefit from a clearer presentation of how Panoptes type II was first identified, what genetic-context or co-occurrence analyses supported its prediction, and how its operon organization compares to previously described Panoptes subtypes. This would help readers better appreciate the novelty and rationale of the study.

3.Cell permeability induced through CARF-TM apo from expression. The PI staining was presented as inconclusive after an induction of 1 or 2 hours but in the cell density and culturability assay they show a growth arrest at 2 hours. Maybe more time-points could be relevant in the PI staining then to observe clearer difference in cell permeability.

4.An additional control in the growth arrest experiments would be helpful. In the growth arrest experiments, only the CARF-TM protein was expressed in the cells and not the mCpol allowing for activity of CARF-TM. It would be interesting to have a control with the two proteins co-expressed to prove that mCpol save the growth arrest phenotype, even though it is already demonstrated in the transformation efficiency experiment.

Minor comments

1.Figure quality should be improved:

a.Add legend including Mg+ in figure 2

b.Figure 3a: no condition with NucC alone as a control

c.Figure 5: legend claims that only cA3 was able to displace the radiolabelled, but it was only the 3'3' and not the 2'3' cA3 isoform. No comment was made on why it is only one of the isoforms allowing this and not the other.

d.Fig 6: In the title it is stated "in oligomerisation of CARF-TM" but the experiments were performed on the truncated version.

------------------------------------

Reviewer #2 (Naama Aviram):

Summary

This study characterizes the type II Panoptes anti-phage guard system, establishing cA3 as its signaling molecule and demonstrating that the CARF-TM effector is held inactive as a dimer when cA3 is bound. Upon cA3 depletion, mimicking phage-encoded sponge or ring nuclease activity, the effector oligomerizes and causes outer membrane permeabilization and growth arrest. The biochemistry is clean and the overall logic of the guard mechanism is well supported. The work is a meaningful extension of the recently described type I Panoptes system and expands our understanding of how bacteria deploy decoy cyclic nucleotides as part of their immune repertoire. This is an important contribution to the field of bacterial anti-phage immunity, and I have several suggestions and concerns that I believe should be addressed to make the paper clearer and stronger.

Major concerns

1.Throughout the study, the heterologous E. coli expression system introduces an important confound that is never acknowledged. The slow mCpol turnover rate, the moderation of CARF-TM toxicity by co-expressed mCpol, and the apparent fitness cost of the system could all reflect suboptimal activity in a non-native host rather than intrinsic properties of the system. In native genomic context, production rates and toxicity may be quite different. This caveat should be explicitly discussed, particularly since the authors themselves note at the end of the Discussion that "further investigation will be required...ideally using a cognate host and genomic expression of the Panoptes system from native promoters". This acknowledgment should be moved forward and given more prominence.

2.The statement that slow mCpol turnover is "likely a common feature of CARF-TM-associated systems" is overstated given that only two enzymes were tested. The claim should be tempered accordingly. Additionally, the functional implications of a slow turnover rate cut both ways and deserve discussion: a low steady-state cA3 pool lowers the threshold for phage-mediated activation, but it also imposes a fitness cost that would likely be negatively selected over evolutionary time. These trade-offs should be addressed.

3.In the section "The soluble CARF variant multimerises when cA3 is not bound": the statement that Crn4 "captures and partially degrades CARF-bound cA3" is not fully supported without rate measurements. If the rate of cA3 degradation by Crn4 is comparable to the intrinsic dissociation rate of cA3 from CARF, then Crn4 may simply be acting on the free released form rather than accessing the bound ligand. This distinction has mechanistic implications and should be discussed, or the claim should be softened.

4.Figure 7 is the most difficult to interpret in its current form and I think the conclusions drawn from it are overstated. Specific issues:

-The growth curves in B are presented on a log scale, which is unusual for this type of experiment - what does the data look like on a linear scale?

- Why does the empty vector condition plateau in panel B? Why does CARF plateau in panel C if OD is still increasing in B?

-CARF-TM cells plateau in OD but drop in CFU, the authors should clarify why this is interpreted as evidence for a VBNC-like state and discuss it more explicitly rather than leaving it implicit. It could be helpful to explain why CFU counts drop.

-The experiment appears to begin at a relatively late logarithmic stage, which compresses the dynamic range of the assay. Starting from a lower OD would give a clearer picture of the growth arrest phenotype.

-Details of this experiment are also missing from the Materials and Methods.

Minor concerns

1.Figure 1 is missing legends for panels C and D.

2.In the section "Type II Panoptes is active in vivo and responds to cA3 levels in cells": NucC is introduced as a control without sufficient context for readers unfamiliar with its role in other defenses. A more detailed explanation of why it serves as a useful control here would help. The order in which conditions are described in the text should also be aligned with the order in which they appear in Figure 3.

3.On the same lines - The statement "as expected, expression of NucC alone resulted in efficient transformation" is confusing: there is no NucC-alone condition in the figure. Do the authors mean the catalytically inactive mCpol mutant? This should be clarified.

4.The conclusion that cA3 binding is tight but not irreversible raises a question worth addressing in the Discussion: how would cA3 turnover occur in the cell? Is there a dedicated ring nuclease in the native host, or does the effector exchange cA3 through some other mechanism?

5.The Sypro Orange data (Figure 6C): the decrease in fluorescence upon Crn4 treatment is interpreted as burial of hydrophobic patches due to aggregation. Are there alternative structural explanations? For example, can the protein unfold and refold without aggregating? This should be briefly considered. More broadly, the connection between the fluorescence data in Figure 6C-D and the proposed structural changes would be strengthened by showing temporal resolution of the signal rather than endpoint measurements alone.

6.Figure 7A requires representative microscopy images illustrating the PI staining phenotype, even if placed in supplementary material.

7.In the section "Expression of apo CARF-TM changes cell permeability in E. coli": the link between outer membrane permeabilization and the observed increase in metabolic rate should be made more explicitly. The logic connecting resazurin reduction kinetics to OM permeability is not immediately clear to the reader.

8.The Discussion would benefit from a deeper comparison of the type I and type II Panoptes phenotypes (e.g. inner membrane collapse and cell death versus outer membrane permeabilization and growth arrest) and from connecting the NucC-mediated response in classical CBASS/type III contexts to the new CARF-TM phenotype described here.

------------------------------------

Reviewer #3:

The manuscript by Grüschow and colleagues describes a type II Panoptes anti-phage defence system in which mCpol synthesises cyclic tri-adenylate (cA3) and the associated CARF-TM effector is kept inactive in the presence of the ligand. The authors show that depletion or sequestration of cA3 activates the effector, causing an increase in membrane permeability and growth arrest. Overall, I found the study interesting, timely, and conceptually important for broadening our understanding of bacterial guard systems.

My main concern is that some of the mechanistic conclusions are stronger than the data currently support, as detailed in my specific comments below. Overall, the authors should make sure they better align the mechanistic claims with the current evidence and, when feasible, strengthen the physiological validation of the model proposed.

Major comments

1.A key conclusion is that cA3 keeps CARF-TM in an inactive dimeric state and that cA3 removal promotes oligomerisation and membrane-associated toxicity. However, the strongest structural and biochemical evidence for this transition comes from the truncated soluble CARF construct rather than the native full-length CARF-TM protein. Since the full-length protein was difficult to purify and tagged versions were non-functional in vivo, the manuscript currently relies in part on inference. I think the authors should either provide stronger data for the full-length effector or soften the wording throughout the manuscript so that it is clear which conclusions are shown directly and which are inferred from the soluble truncation.

2.The study is built largely on heterologous expression in E. coli and plasmid challenge assays. These experiments are useful and consistent, but they do not directly demonstrate antiviral activity in a native or near-native context. The manuscript would be stronger with phage infection data, or at minimum with more explicit acknowledgment that the biological role of the system is inferred rather than directly demonstrated here.

3.The data support the conclusion that apo CARF-TM affects cell physiology and membrane properties, but the claim of outer membrane permeabilization seems somewhat indirect. The Nile Red, resazurin, polymyxin B comparison, and plating assays are suggestive, but they do not fully distinguish outer membrane effects from broader envelope stress or indirect downstream consequences of inner membrane perturbation. I suggest moderating the language here unless additional direct membrane assays are included.

4.In figure 3, quantification of transformation efficiency across biological replicates would make the conclusions more rigorous and easier to assess.

Minor comments

1.The manuscript should be careful not to overstate linkage assignment if the exact cA3 isomer was not established directly in this study.

2.The Discussion is thoughtful, but several statements about the localisation and orientation of CARF-TM in the membrane seem more confident than the data justify.

3.The data availability statement should be finalised before publication, as it currently indicates that the DOI is still pending.

# Author comment: Author response to Decision Letter 2

**DOI:** 10.1371/journal.pbio.3003934.r003

3 Jun 2026

Attachment Submitted filename: Response to Reviewers.pdf

# Peer review: Decision Letter 2

**DOI:** 10.1371/journal.pbio.3003934.r004

**Authors:** Melissa Vazquez Hernandez

1 Jul 2026

Dear Michael,

Thank you for your patience while we considered your revised manuscript "Cyclic tri-adenylate signalling by a Panoptes anti-phage guard system with a CARF-TM effector" for publication as a Research Article at PLOS Biology. This revised version of your manuscript has been evaluated by the PLOS Biology editors, the Academic Editor and the original reviewers. Please accept my apologies for the delay.

Based on the reviews, we are likely to accept this manuscript for publication, provided you satisfactorily address the following data and other policy-related requests.

1) We routinely suggest changes to titles to ensure maximum accessibility for a broad, non-specialist readership, and to ensure they reflect the contents of the paper. In this case, we would suggest a some edits to the title, as follows. Please ensure you change both the manuscript file and the online submission system, as they need to match for final acceptance:

"Cyclic tri-adenylate controls a CARF-TM effector in type II Panoptes anti-phage systems"

2) Please add the weblink of the funding agencies in the Financial Disclosure statement in the manuscript details.

3) You may be aware of the PLOS Data Policy, which requires that all data be made available without restriction: http://journals.plos.org/plosbiology/s/data-availability. For more information, please also see this editorial: http://dx.doi.org/10.1371/journal.pbio.1001797

Thank you for providing already the raw data from some figures. However, we are missing the numerical values for the following figures:

Figure 3E, 7E, S1AB, S4AB, S6, S8AB

NOTE: the numerical data provided should include all replicates AND the way in which the plotted mean and errors were derived (it should not present only the mean/average values).

*Additionally, it is not clear to which panel the numbers from Figure 5 and 6 belong to. Could you please specify?

4) Please cite the location of the data clearly in all relevant main and supplementary Figure legends, e.g. “The data underlying this Figure can be found in S1 Data” or “The data underlying this Figure can be found in https://doi.org/10.5281/zenodo.XXXXX”

5) Please ensure that you are using best practice for statistical reporting and data presentation. These are our guidelines https://journals.plos.org/plosbiology/s/best-practices-in-research-reporting#loc-statistical-reporting and a useful resource on data presentation https://journals.plos.org/plosbiology/article?id=10.1371/journal.pbio.1002128

6) If you are reporting experiments where n ≤ 5, please plot each individual data point.

7) We require the original, uncropped and minimally adjusted images supporting all blot and gel results reported in the Figures S4CD, S9

8) Please add a scale bar in all microscopy pictures

9) Please ensure that your Data Statement in the submission system accurately describes where your data can be found and is in final format, as it will be published as written there

10) Per journal policy, if you have generated any custom code during the course of this investigation, please make it available without restrictions. Please ensure that the code is sufficiently well documented and reusable, and that your Data Statement in the Editorial Manager submission system accurately describes where your code can be found. More information on our Code Policy, what and how to share can be found here: https://journals.plos.org/plosbiology/s/code-availability

Please note that we cannot accept sole deposition of code in GitHub, as this could be changed after publication. However, you can archive this version of your publicly available GitHub code to Zenodo. Once you do this, it will generate a DOI number, which you will need to provide in the Data Accessibility Statement (you are welcome to also provide the GitHub access information). See the process for doing this here: https://docs.github.com/en/repositories/archiving-a-github-repository/referencing-and-citing-content

As you address these items, please take this last chance to review your reference list to ensure that it is complete and correct. If you have cited papers that have been retracted, please include the rationale for doing so in the manuscript text, or remove these references and replace them with relevant current references. Any changes to the reference list should be mentioned in the cover letter that accompanies your revised manuscript.

In addition to these revisions, you may need to complete some formatting changes, which you will receive in a follow up email. A member of our team will be in touch with a set of requests shortly. If you do not receive a separate email within a few days, please assume that checks have been completed, and no additional changes are required.

We expect to receive your revised manuscript within two weeks.

To submit your revision, please go to https://www.editorialmanager.com/pbiology/ and log in as an Author. Click the link labelled 'Submissions Needing Revision' to find your submission record. Your revised submission must include the following:

- a cover letter that should detail your responses to any editorial requests, if applicable, and whether changes have been made to the reference list

- a Response to Reviewers file that provides a detailed response to the reviewers' comments (if applicable, if not applicable please do not delete your existing 'Response to Reviewers' file.)

- a track-changes file indicating any changes that you have made to the manuscript.

NOTE: If Supporting Information files are included with your article, note that these are not copyedited and will be published as they are submitted. Please ensure that these files are legible and of high quality (at least 300 dpi) in an easily accessible file format. For this reason, please be aware that any references listed in an SI file will not be indexed. For more information, see our Supporting Information guidelines:

https://journals.plos.org/plosbiology/s/supporting-information

*Published Peer Review History*

Please note that you may have the opportunity to make the peer review history publicly available. The record will include editor decision letters (with reviews) and your responses to reviewer comments. If eligible, we will contact you to opt in or out. Please see here for more details:

https://plos.org/published-peer-review-history/

*Press*

Should you, your institution's press office or the journal office choose to press release your paper, please ensure you have opted out of Early Article Posting on the submission form. We ask that you notify us as soon as possible if you or your institution is planning to press release the article.

*Protocols deposition*

To enhance the reproducibility of your results, we recommend that if applicable you deposit your laboratory protocols in protocols.io, where a protocol can be assigned its own identifier (DOI) such that it can be cited independently in the future. Additionally, PLOS ONE offers an option for publishing peer-reviewed Lab Protocol articles, which describe protocols hosted on protocols.io. Read more information on sharing protocols at https://plos.org/protocols?utm_medium=editorial-email&utm_source=authorletters&utm_campaign=protocols

Please do not hesitate to contact me should you have any questions.

Sincerely,

Melissa

Melissa Vazquez Hernandez, Ph.D.

Senior Editor

mvazquezhernandez@plos.org

PLOS Biology

------------------------------------------------------------------------

REVIEWERS' COMMENTS

------------------------------------------------------------------------

Reviewer #1:

We thank the authors for fully adressing our comments and recomment publications

------------------------------------------------------------------------

Reviewer #2:

The authors have done an excellent job addressing the reviewers' concerns.

The new data significantly strengthens the manuscript, and the expanded discussion effectively contextualizes the work and conveys why it is timely and meaningful.

It was a pleasure to read this revised version. I have no further comments and recommend acceptance.

------------------------------------------------------------------------

Reviewer #3:

The authors have now substantially improved the manuscript and the major concerns raised in the previous round have been satisfactorily addressed. The authors have added new phage infection data showing that the type II Panoptes system can provide defence against phages encoding Acb1, and that escape happens when the acb1 gene is disrupted. These experiments strengthen the physiological relevance of the proposed model. The authors have also moderated several of the mechanistic claims and acknowledge the limitations of the membrane-permeability assays and the heterologous E. coli expression system. Overall, I think the revised manuscript is acceptable for publication in PLOS Biology.

# Author comment: Author response to Decision Letter 3

**DOI:** 10.1371/journal.pbio.3003934.r005

14 Jul 2026

Attachment Submitted filename: Response to reviewers.docx

# Editor report: Decision Letter 3

**DOI:** 10.1371/journal.pbio.3003934.r006

**Authors:** Melissa Vazquez Hernandez

20 Jul 2026

Dear Dr White,

I hope all is well.

Thank you for the submission of your revised Research Article "Cyclic tri-adenylate controls a CARF-TM effector in type II Panoptes anti-phage systems" for publication in PLOS Biology. On behalf of my colleagues and the Academic Editor, Jeremy Barr, I am pleased to say that we can in principle accept your manuscript for publication, provided you address any remaining formatting and reporting issues. These will be detailed in an email you should receive within 2-3 business days from our colleagues in the journal operations team; no action is required from you until then. Please note that we will not be able to formally accept your manuscript and schedule it for publication until you have completed any requested changes.

I also wanted to let you know that we have commissioned a primer to accompany the study and highlight the findings. This might delay the publication process but it helps to bring attention to the study. I'll get back to you once the primer is submitted.

Please take a minute to log into Editorial Manager at http://www.editorialmanager.com/pbiology/, click the "Update My Information" link at the top of the page, and update your user information to ensure an efficient production process.

PRESS

We frequently collaborate with press offices. If your institution or institutions have a press office, please notify them about your upcoming paper at this point, to enable them to help maximise its impact. If the press office is planning to promote your findings, we would be grateful if they could coordinate with biologypress@plos.org. If you have previously opted in to the early version process, we ask that you notify us immediately of any press plans so that we may opt out on your behalf.

We also ask that you take this opportunity to read our Embargo Policy regarding the discussion, promotion and media coverage of work that is yet to be published by PLOS. As your manuscript is not yet published, it is bound by the conditions of our Embargo Policy. Please be aware that this policy is in place both to ensure that any press coverage of your article is fully substantiated and to provide a direct link between such coverage and the published work. For full details of our Embargo Policy, please visit http://www.plos.org/about/media-inquiries/embargo-policy/.

Thank you again for choosing PLOS Biology for publication and supporting Open Access publishing. We look forward to publishing your study.

Sincerely,

Melissa

Melissa Vazquez Hernandez, Ph.D., Ph.D.

Senior Editor

PLOS Biology

mvazquezhernandez@plos.org
